# Analysis of gene expression in rheumatoid arthritis and related conditions offers insights into sex-bias, gene biotypes and co-expression patterns

**DOI:** 10.1371/journal.pone.0219698

**Published:** 2019-07-25

**Authors:** Alexander Platzer, Thomas Nussbaumer, Thomas Karonitsch, Josef S. Smolen, Daniel Aletaha

**Affiliations:** 1 Division of Rheumatology, Department of Medicine III, Medical University of Vienna, Vienna, Austria; 2 Chair and Institute of Environmental Medicine, UNIKA-T, Technical University and Helmholtz Zentrum München, Augsburg, Germany; 3 Institute of Network Biology (INET), Helmholtz Center Munich, Neuherberg, Germany; Universite Paris-Sud, FRANCE

## Abstract

The era of next-generation sequencing has mounted the foundation of many gene expression studies. In rheumatoid arthritis research, this has led to the discovery of important candidate genes which offered novel insights into mechanisms and their possible roles in the cure of the disease. In the last years, data generation has outstripped data analysis and while many studies focused on specific aspects of the disease, a global picture of the disease is not yet accomplished. Here, we analyzed and compared a collection of gene expression information from healthy individuals and from patients suffering under different arthritis conditions from published studies containing the following clinical conditions: early and established rheumatoid arthritis, osteoarthritis and arthralgia. We show comprehensive overviews of this data collection and give new insights specifically on gene expression in the early stage, into sex-dependent gene expression, and we describe general differences in expression of different biotypes of genes. Many genes that are related to cytoskeleton changes (actin filament related genes) are differently expressed in early rheumatoid arthritis in comparison to healthy subjects; interestingly, eight of these genes reverse their expression ratio significantly between men and women compared early rheumatoid arthritis and healthy subjects. There are some slighter changes between men and woman between the conditions early and established rheumatoid arthritis. Another aspect are miRNAs and other gene biotypes which are not only promising candidates for diagnoses but also change their expression grossly in average at rheumatoid arthritis and arthralgia compared to the healthy condition. With a selection of intersecting genes, we were able to generate simple classification models to distinguish between healthy and rheumatoid arthritis as well as between early rheumatoid arthritis to other arthritides based on gene expression.

## Introduction

Rheumatoid arthritis (RA) is a chronic, complex, systemic, multifactorial disease [1, 2] with a prevalence of 0.3–1% in the population worldwide [3], affecting women 2–3 times more often than men. The proven or at least strongly suspected etiopathogenetic factors include genomic variations [4], gene expression changes [5], autoimmunity [6] and environmental factors [7]. No factor is considered as single cause, except for the (currently unknown) cause of the first insult leading to the autoimmune inflammation characteristic of RA. Likely, there is not one single cause of RA, no single path to progression and no single curative approach, as ultimate success rates of single therapies are limited [8, 9]. This has led to the hypothesis that there might be RA subtypes with different RA disease manifestations that are dependent on sex, genotype, gene expression or on the composition of the microbiome, which would make RA an important showcase for personalized medicine.

A lot of effort has been undertaken in finding or assessing specific genes and pathways of importance for the progression of RA [10–13]. Less effort has been spent so far to obtain a more complete view of the complete genome and expression data. The analysis of whole genome sequencing data of RA is covered in broader GWAS approaches (e.g. [4, 14], where several associated SNPs were reported), while gene expression data from RNA-seq is more left open for the broader view and was generated for particular research questions [15, 16]. Some broader views of gene expression are published based on microarray data [17–19], as well as some narrower views based on quantitative polymerase chain reactions (PCRs) of several genes [20–22]. The prevalence of RA is lower in men than in women [23–26], but it is unclear whether this is also related to gene expression; reported relations with sex hormones such as estrogen and androgen would be supportive of this hypotheses [27]. If such major differences of gene expression between men and women exist specifically in RA compared to healthy subjects, these genes might be targets for further investigation as there might be sex-specific issues beside the prevalence.

Some miRNAs have been also reported as related to RA, with the main motivation to use them as diagnostic markers for RA [28, 29]. Despite the different alternatives for the initial starting points of RA and potential diagnostic markers, there is a consensus regarding the center of amplification and perpetuation of joint inflammation: the synovial tissue [30, 31]. Uncontrolled persistent inflammation of the synovial membrane leads to progressive joint damage and disability [2]. For this reason, we focus the present analyses on gene expression (RNA-seq) data from synovial tissue. We use published studies with large amounts of RNA-seq data in populations of early and established RA, as well as in patients with related diagnoses [15, 16].

## Results

### Clustering

#### Clustering of subjects and conditions

**Fig 1:**
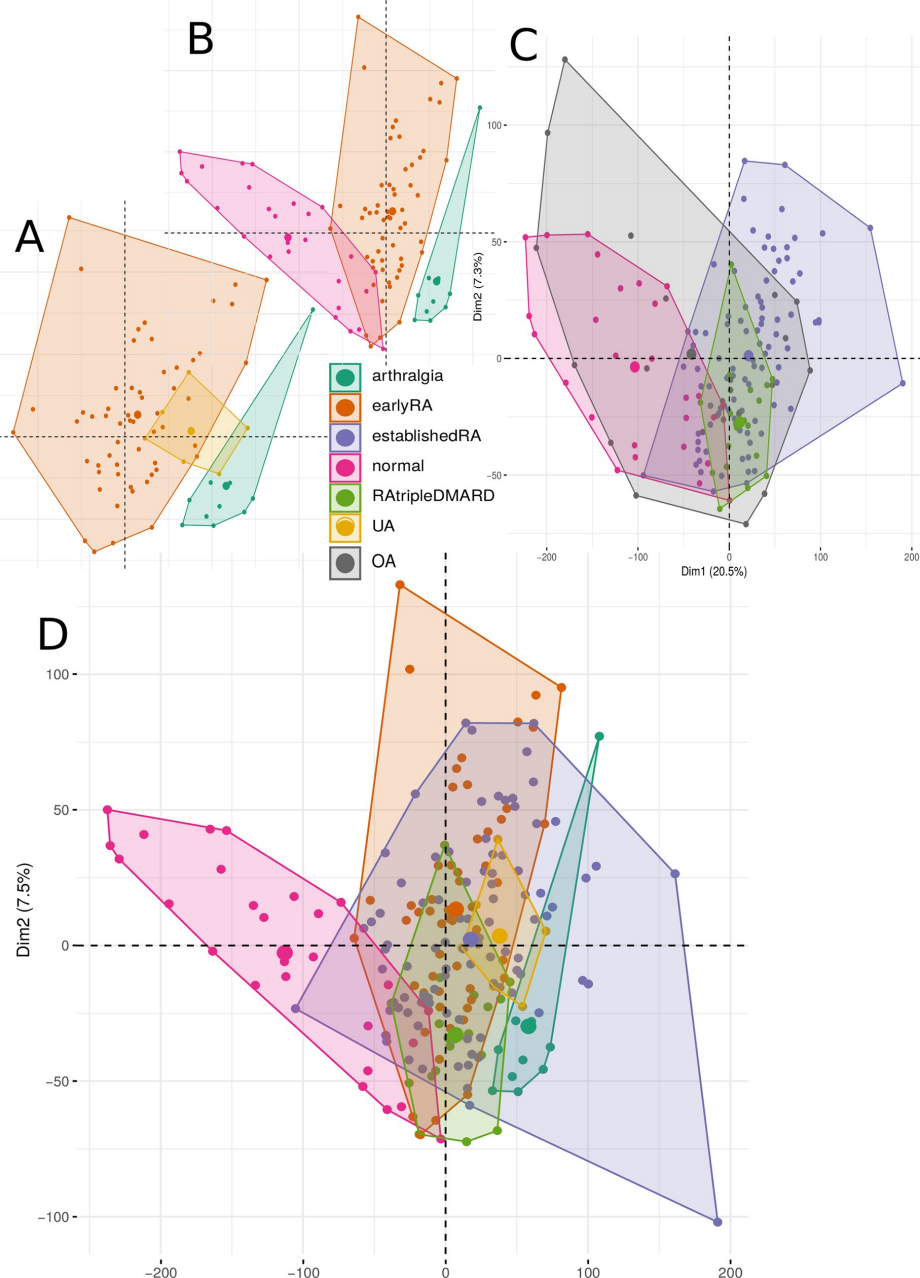
The first two principal components of the PCA based on the RPKMs of the coding genes. The areas are the convex hulls of the conditions. The largest point of one color depicts the center of a hull. A, B, and D are the same PCA analysis with the same coordinates, where in D all conditions except OA are visible, in A and B only three of them for a better overview. C is a PCA with OA, where four conditions are shown to depict the variability of OA. Number of samples: 10 arthralgia, 57 earlyRA, 95 establishedRA, 27 normal, 22 OA, 19 RAtripleDMARD and 6 UA.

**Fig 2:**
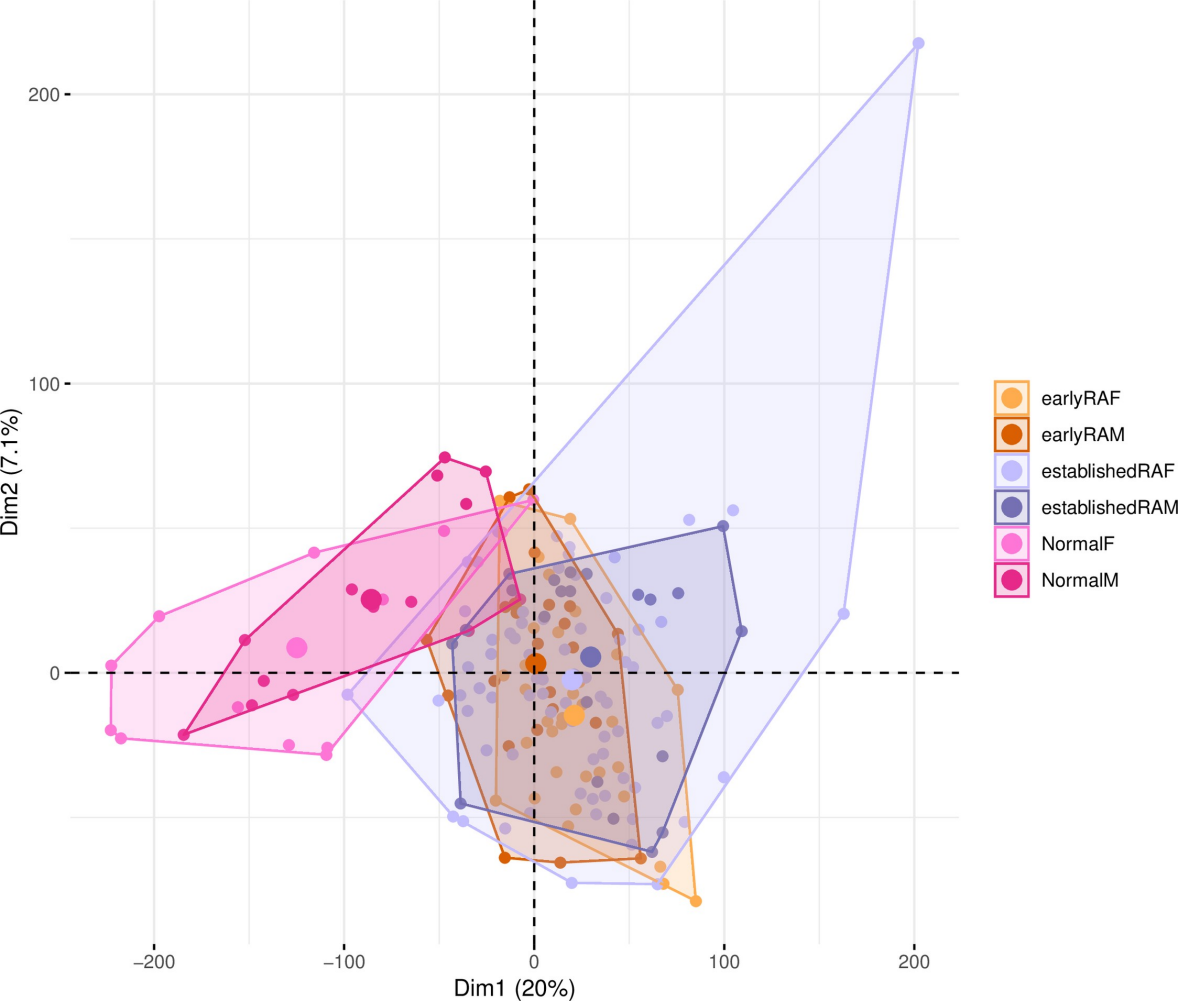
The first two principal components of the PCA considering RPKMs of the coding genes. The areas are the convex hull of the condition. The largest point of one color depicts the centers of the hull. Only those conditions are shown where more than ten samples were available for male and female individuals. Number of samples: 33 earlyRAF, 24 earlyRAM, 73 establishedRAF, 22 establishedRAM, 13 NormalF, 14 NormalM.

We applied different clustering and dimension reduction methods to obtain a comprehensive view of the transcriptome data from the 236 RNA-seq synovial biopsy samples. A PCA is shown in Fig 1, divided in panels, where only some conditions are shown (because of large overlaps; all conditions together are shown in S1 Fig). On a high-level view, gene expression of healthy subjects is quite different compared to the non-healthy conditions (Fig 1D; classification model accuracy of 95%, p-value of separation between healthy and non-healthy (excluding OA) is 4.4*10^−18^ when the coordinates of the first two components of the PCA and the labels for the samples were taken and treated as a classification problem for the tree learner learner C4.5 [32]). Established RA is quite broad and overlaps with all other groups. Arthralgia is clearly different from early RA as the convex hull is not overlapping at all (Fig 1). Different clustering approaches do not entail more insights than PCA (Conformal Eigenmaps [33], multidimensional scaling [34] and Sammon mapping [35], see S2–S4 Figs). Fig 2 shows the same overview for the conditions when split by sex and by only using the three conditions where more than ten samples for men and women exist (conditions early RA, established RA and healthy; for an overview of the first 10 principal components see S5 Fig). There is not much visible difference between men and women, as all conditions are highly overlapping in the first two principal components whether samples originate from male or female individuals.

Looking into clustering within one diagnosis reveals potential subtypes (PCA and hierarchical clustering in Supplementary Archive FiguresClusteringWithinClasses/). For example, there is a kind of female cluster within early RA (Supplementary Fig 'earlyRA_hclust'). When filtering for genes, which are significantly differently expressed in at least one comparison to normal, the similarity of conditions based on the fold-change can be assessed (S6 Fig). OA is there located next to samples from healthy individuals, a strong treatment (RAtripleDMARD) constitute the next neighbor and early/established RA conditions group apart. When restricting to miRNAs, the picture is similar: only the closer conditions are a little bit rearranged (S7 Fig). Hierarchical clustering instead of neighbor joining and PCA are shown in S8 Fig for coding genes and in S9 Fig for miRNAs.

### Gene enrichment analysis

**Fig 3:**
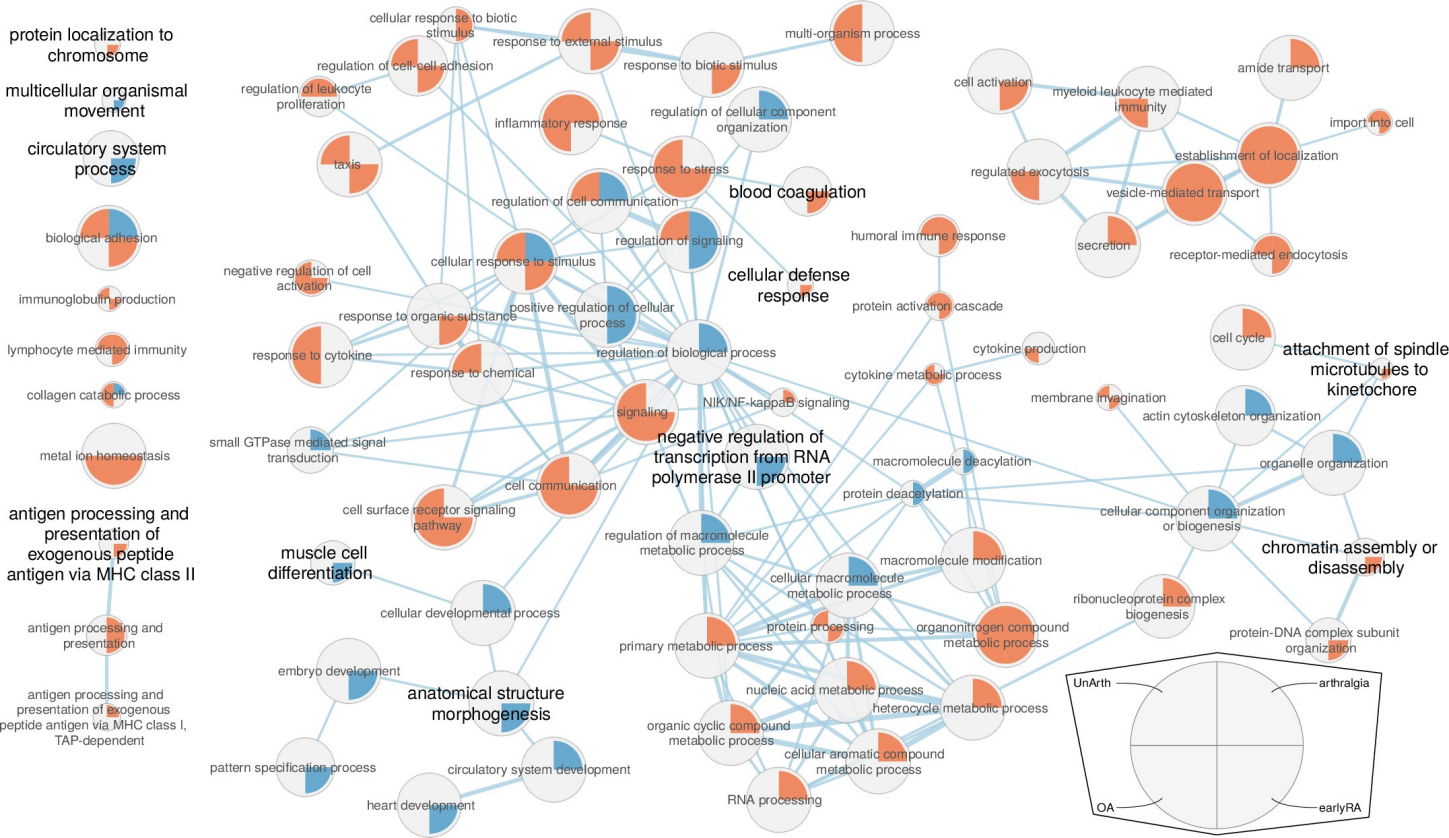
GO (BP) term enrichments of DEGs in earlyRA, arthralgia, OA and undifferentiated arthritis. Base state is normal, each term has the DEG enrichment of the four conditions in the circle's quadrants according to the legend bottom right. Red indicates there is an enrichment in the upregulated DEGs, blue indicates an enrichment in the down-regulated DEGs and gray indicates no enrichment. The node size represents the number of genes in the annotation for that term. The edge thickness represents the degree of overlap between the gene-sets of two terms. The terms in a larger font are a selection for meaningful terms specifically for earlyRA (somewhat arbitrary). See methods and main text for filtering and discussion. Number of samples: 10 arthralgia, 57 earlyRA, 22 OA and 6 UA.

In order to better understand the differences in gene functions between the different conditions, we performed a gene enrichment analysis. We looked for GO [36, 37], KEGG [38] and REACTOME [39] enriched terms in the significantly differentially expressed genes between the clinical conditions and various derived gene lists. This includes all gene lists used and generated in this article (see in the Supplementary Archive tables/ and geneSets/ for all enrichments and gene lists). An overview of GO (BP) term enrichments of the comparisons of normal to earlyRA, arthralgia, OA and undifferentiated arthritis is shown in Fig 3. More details about tools and strict filtering settings needed for a diagram fitting onto a single page are in the method section. The GO terms in a larger font therein were selected for their specifity for earlyRA and meaningfulness. For example, in the upper right cluster, the term 'vesicle-mediated transport' might be interesting, but is enriched in the up-regulated genes of all four conditions. The term 'cell activation' is specifically enriched in up-regulated genes in earlyRA, but the term itself is rather nonspecific. Taken the GO terms of DEGs in earlyRA together, there is specifically more expression for chromatin (lower right in Fig 3), coagulation factors (as also reported in several articles [40–42]), less activity of polymerase II (as can also be seen in section 'Different gene expression at different gene biotypes'), less muscle cell activity (see section 'Different RA gene expression in men and women' for a more detailed different view on that) and more antigen presentation (left side in Fig 3). Other patterns in this view are also interesting, like enrichment specifically for earlyRA and undifferentiated arthritis as these conditions are clinically quite close. For example, the Gene Ontology terms 'biological adhesion', 'regulation of cell-cell adhesion' and 'immunoglobulin production' are enriched in earlyRA and UA, but not in OA and arthralgia (left in Fig 3). Unfortunately, there are only few samples for undifferentiated arthritis, which weakens the hints from these patterns. The complete enrichment lists in the Supplement give a more detailed view, in the main text and in the next sections we focus on single effects on the top-level.

### Different RA gene expression in men and women

**Fig 4:**
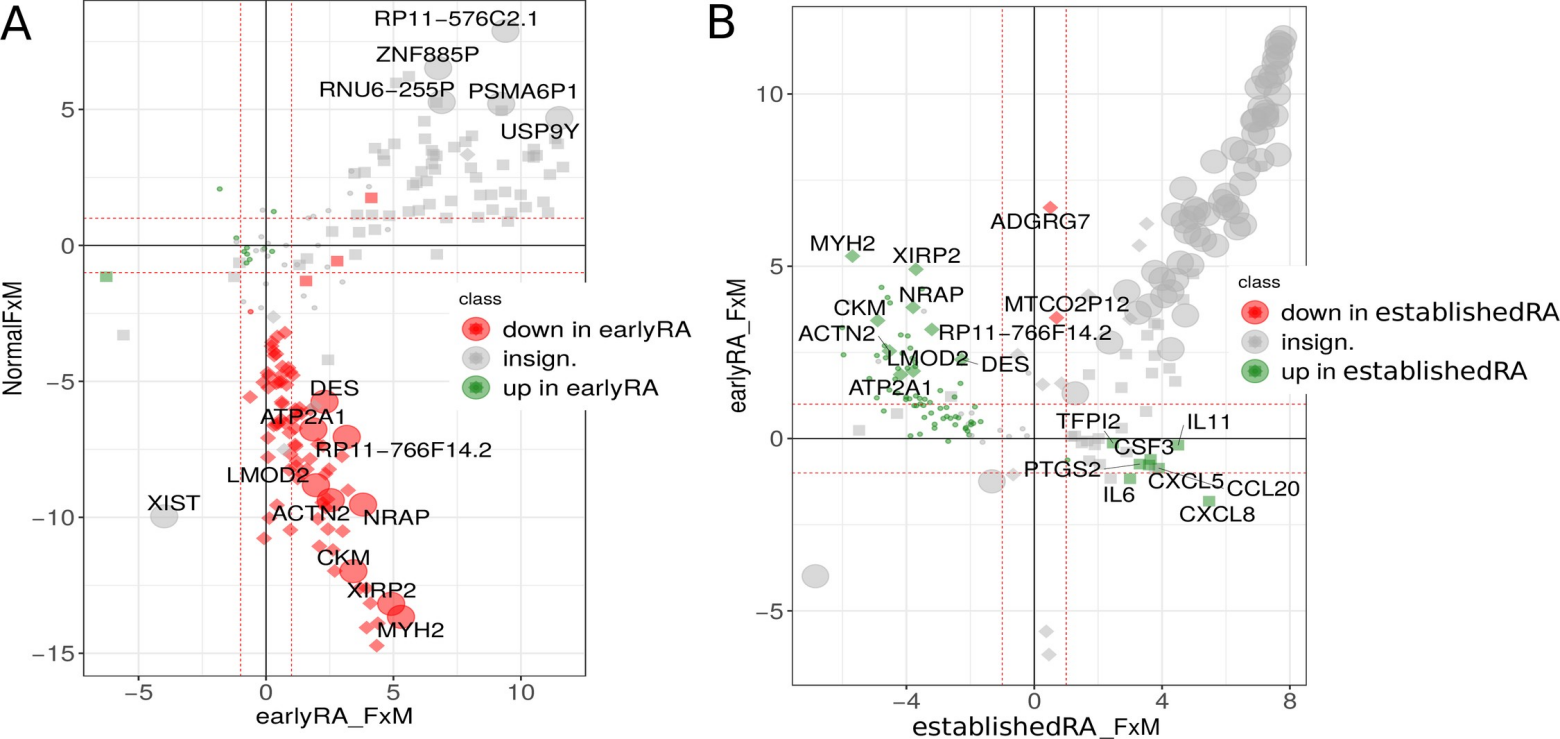
Log_2_ fold-changes of gene expression between men and women in early RA, established RA and normal condition. Only genes are shown which are significantly differentially expressed in men and women. (A) The sex-ratio of gene expression in early RA and normal condition. The size and shape shows the significance in differences of men and women: the large circles are genes significantly differentially expressed between the sexes in early RA and normal condition, these genes are also labelled. Small squares mean a significant difference between men and woman only in early RA, small diamonds mean a significant difference only between healthy men and woman. The color represents the significance of the difference in expression between normal and early RA. (B) The sex-ratio of gene expression in established RA and early RA. There are no gene significantly differentially expressed in men and women in established RA and early RA and differentially expressed between established RA and early RA. Genes are named if one of the gene expression sex-ratios is significant: squares mean a significant difference between men and woman only in established RA, small diamonds mean a significant difference only between men and woman in early RA.

Within each clinical condition there are 85 to 101 genes differentially expressed when comparing men and women within the 236 RNA-seq synovial biopsy samples. Some of these genes are also differentially expressed in early RA compared to normal condition and some of these genes reverse their expression sex-ratio between normal and early RA. This is shown in Fig 4A, where all genes exhibiting a threefold change can be found in the lower right quadrant. This means that there exist not only genes which are significantly differentially expressed when comparing the normal condition with early RA, but of which some are also significantly lower in healthy males and significantly higher in males with early RA. These genes are ATP2A1, LMOD2, ACTN2, DES, CKM, NRAP, MYH2, XIRP2 and RP11−766F14.2 where all but the last are related to muscles according to the GeneCards database [43] (muscle filament, sarcomere and actin filament). A Gene Ontology term enrichment shows the same, all terms assigned to more than one of the nine genes are related to muscles and actin organization (see Supplementary Archive tables/ for enrichment, where this set is named 'earlyRAdown_NormalFM_down_earlyRAFMup_ageFilter'). As no muscle cells should be present in synovial biopsies, the substantial GO-term in this context refers to cytoskeleton changes (change of expression of actin filament related genes). For RP11-766F14.2 only little is known—maybe because of its just recent aliases [44], where its role in obliterative portal venopathy is described.

The same comparison between early and established RA (Fig 4B) has no threefold significantly differentially expressed genes, but shows two things: the aforementioned genes for cytoskeleton changes are different between earlyRA and establishedRA, this special difference in men and women is only present at earlyRA (see also S10 Fig for the same comparison between establishedRA and normal condition), and secondly, that some cytokines and the two genes PTGS2 and TFPI2 are stronger expressed in men with established RA. Beside the cytokines, also the latter two genes have been also investigated for RA (PTGS2 is more often referred to as COX-2) [45–47].

The comparison between OA and normal condition is unremarkable (S11 Fig).

### Different gene expression at different gene biotypes

**Fig 5:**
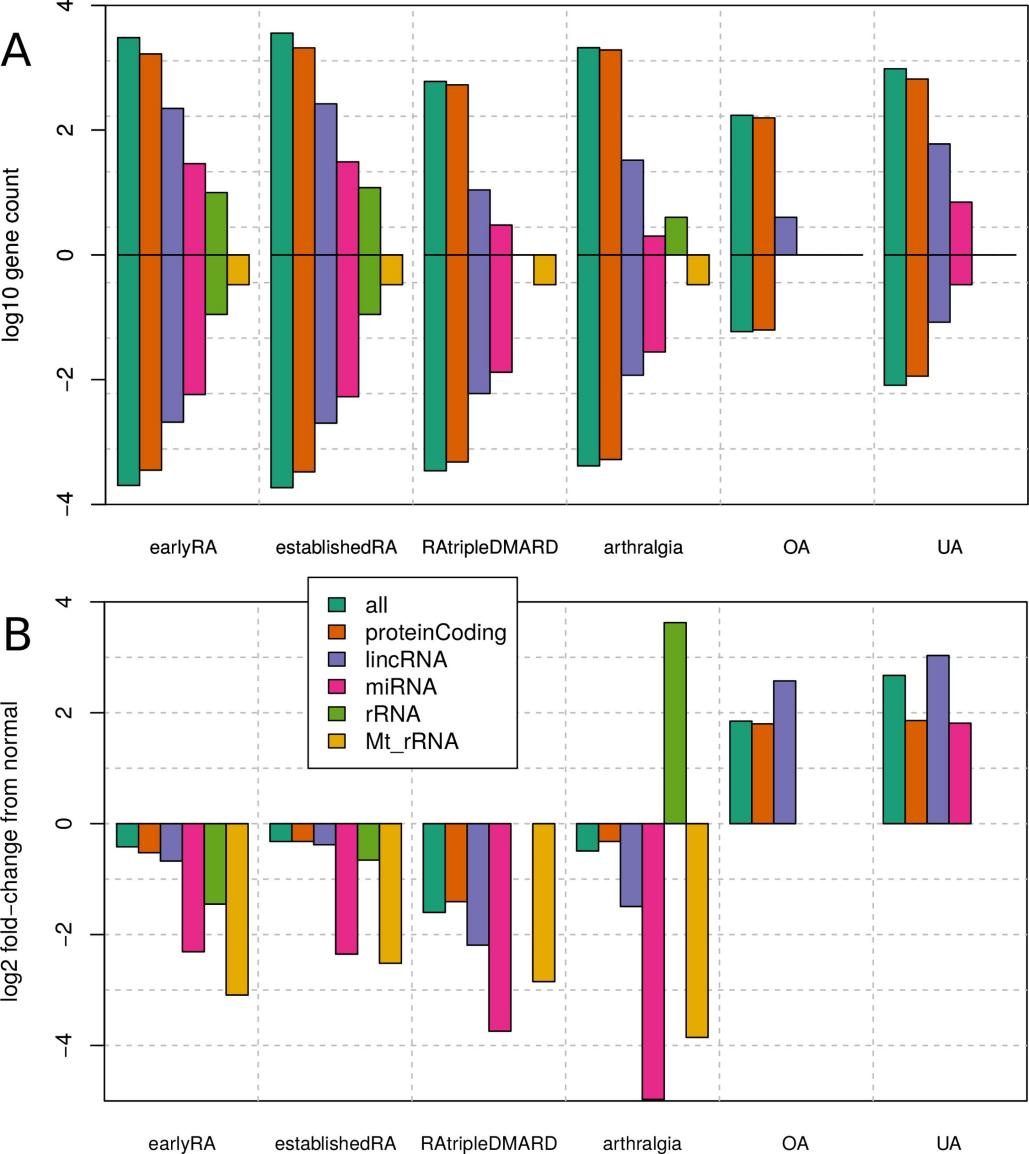
Average fold-changes and counts of different biotypes of genes. The labels on the x-axis mean the change of this condition relative to normal. Only significant changes are regarded. The labels for the biotypes of genes are defined by Ensembl (the biotypes of genes). Missing bars mean that there was no significant change in any gene of this biotype. (A) The log_10_ count of the significantly differentially expressed gene by biotype. Each column consists of two bars: from 0 to the positive side are the (log_10_) numbers of significantly higher expressed genes, from 0 to the negative side are the numbers of significantly lower expressed genes. (B) The average log_2_ fold-change of the significantly differentially expressed genes. It is to see that the average log_2_ fold-change roughly corresponds in the difference of the counts of the significantly higher and lower expressed genes. Number of samples: 10 arthralgia, 57 earlyRA, 95 establishedRA, 27 normal, 22 OA, 19 RAtripleDMARD and 6 UA.

We then assessed the average expression change of different gene biotypes (as defined as biotypes by Ensembl [48]) in the 236 RNA-seq synovial biopsy samples (Fig 5 and S2 Table). The base state is defined there as the normal condition. The positive and negative average fold-changes in Fig 5B are roughly corresponding to the count of significantly differently expressed genes (corresponds to the difference in counts; Fig 5A). rRNAs of mitochondria are less expressed in RA conditions and arthralgia, while the normal rRNA is much higher expressed in arthralgia and miRNAs are less expressed in RA conditions and arthralgia. Generally, there is a pattern of lower gene expression in RA and arthralgia. It seems unexpected that in this sense undifferentiated arthritis is not similar to the RA conditions, as undifferentiated arthritis has clinical signs of synovitis, but 'just' failing to meet the 2010 American College of Rheumatology criteria [49] for RA. When taking a closer look, it does not look contradictory; in both conditions many genes related to the immune system are highly up-regulated, but in RA even more genes are down-regulated (more than up-regulated and many more than down-regulated in undifferentiated arthritis). The down-regulated genes in early RA seem to have very different functions compared with the up-regulated genes, except genes related to immune system activation; the most prominently enriched GO-terms are related to cytoskeleton changes (see Supplementary Archive tables/ for the various term enrichments of these genes).

### Classification models

**Fig 6:**
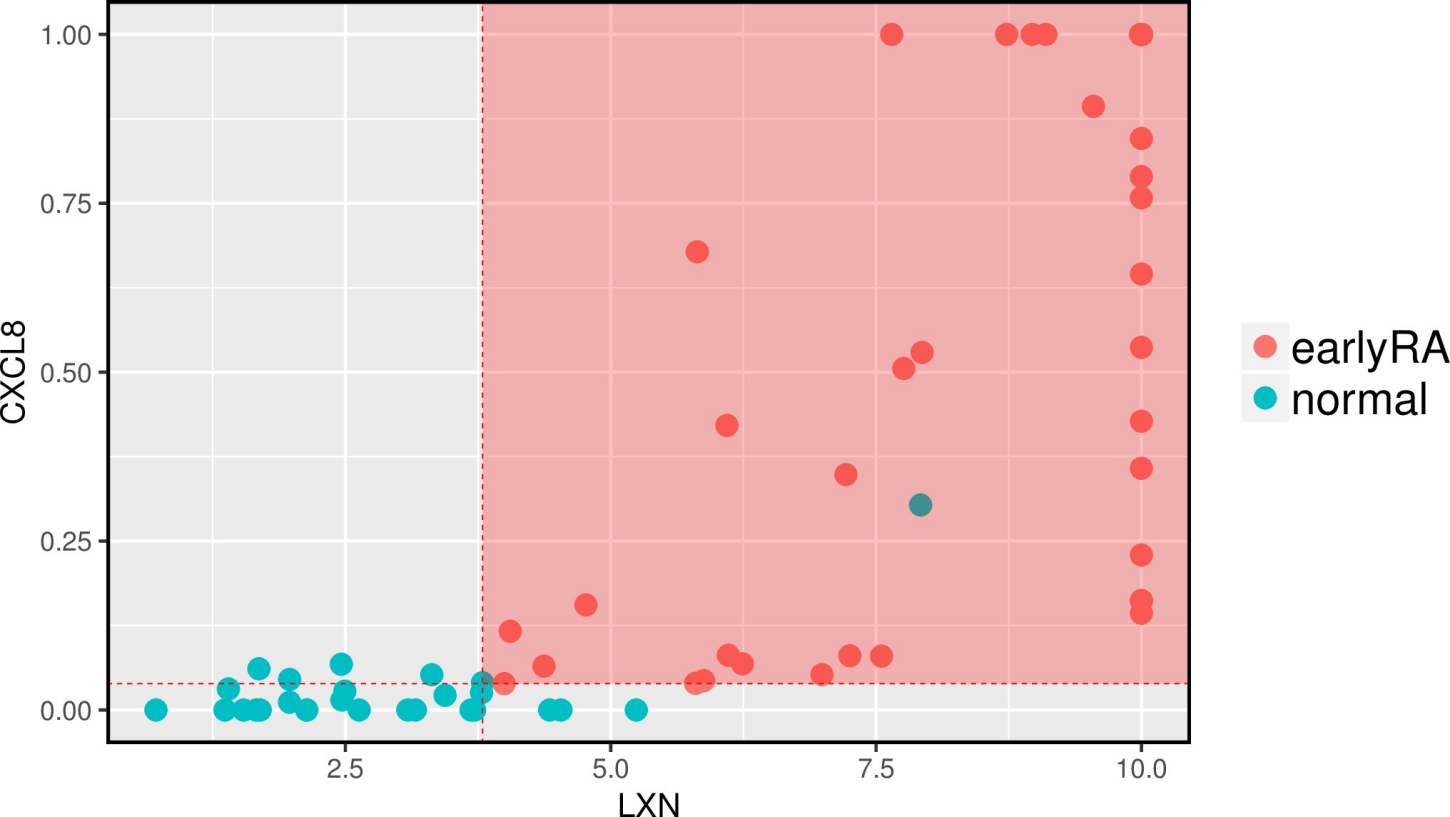
Classification model for distinguish between early RA and normal based on RPKMs. The model consists only of the single rule LXN > 3.8 AND CXCL8 > 0.04 -> early RA. It corresponds to an accuracy of 92% at the 10-fold cross-validation (p-value 2.02*10^−13^). Variables for model-generation were pre-selected upon the intersection of single-variable comparisons. This pre-selection weakens the cross-validation as it is no part of it. This model is only intended for a distinction between early RA and normal as simple as possible based on gene expression. In total there are 84 samples. The RPKM values are cut at 10 and at 1, respectively.

**Fig 7:**
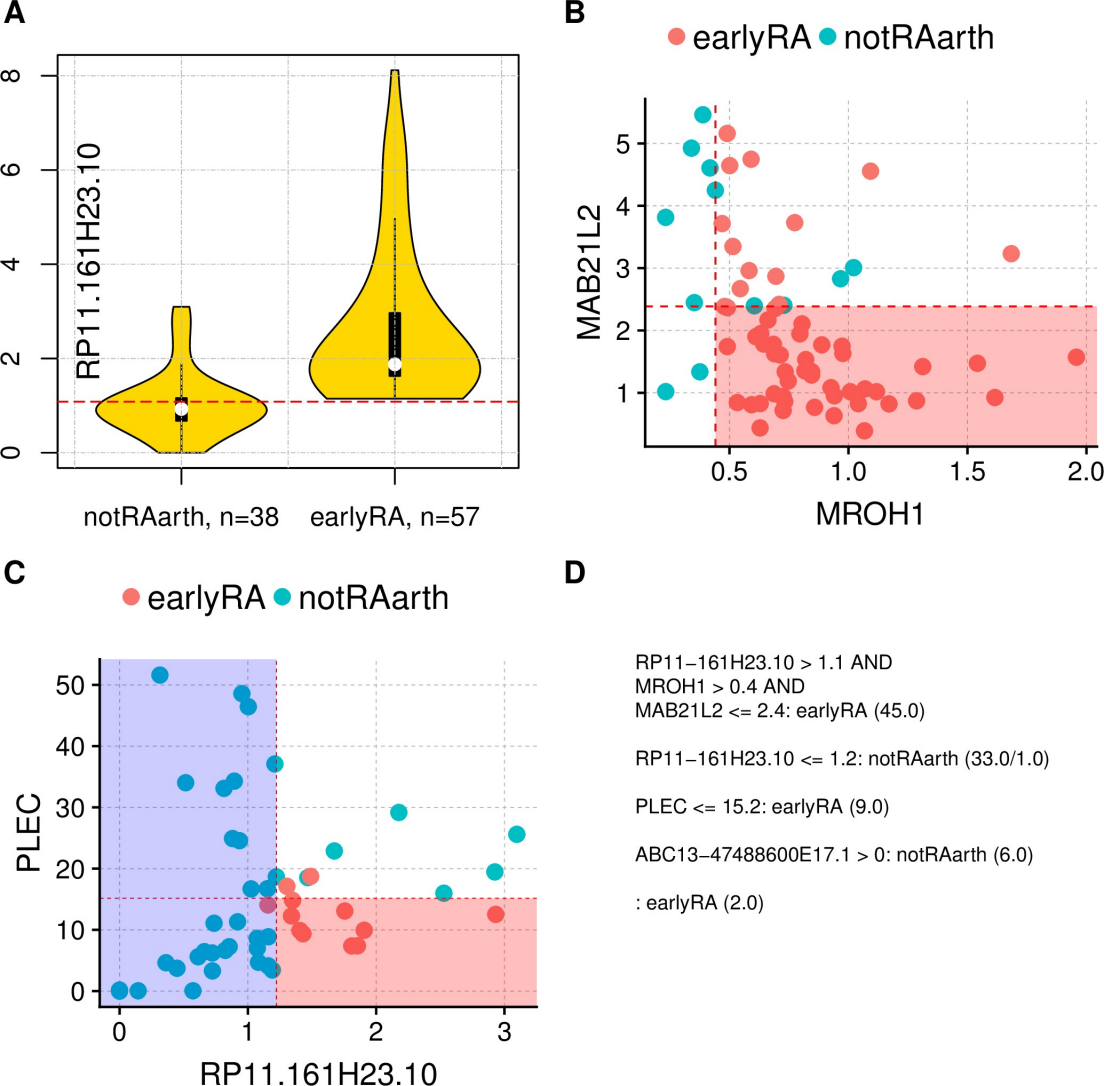
Classification model for distinguish between early RA and not healthy but also not RA based on RPKMs. 'not RA' as present in the data, which is OA, arthralgia and undifferentiated arthritis, shortcut 'notRAarth'. The model consists of four rules, where three of them are shown here graphically. Panels A and B depict the first rule, where in B are only cases left which are higher than the threshold in A. The red shaded area in B shows the cases hit by the first rule, which are all earlyRA. Panel C shows the second and third rule, where the model output for the red shaded area is earlyRA and for the blue shaded area is notRAarth. The complete model as text is shown in D. The threshold values are rounded to one decimal place. The model corresponds to an accuracy of 86% at the 10-fold cross-validation (p-value 1.7*10^−12^). Variables for model-generation were pre-selected upon the intersection of single-variable comparisons. This pre-selection weakens the cross-validation as it is no part of it. This model is only intended for a distinction between early RA and other conditions as simple as possible based on gene expression. In total there were 95 samples as input data.

Based on the RNA-seq samples and certain gene sets, we were able to generate classification models with significance in an internal cross-fold validation to distinguish early RA from the normal condition and early RA from other diagnoses (Fig 6 and Fig 7). For the comparison of early RA with normal condition, we selected the intersection of genes differentially expressed in the single-variable comparisons normal vs early RA, normal vs OA, normal vs. undifferentiated arthritis and normal vs. arthralgia. This resulted in 45 differentially expressed genes. RPKMs of these genes are the input for generating models. One of the best and also quite the simplest model is a PART [50] model with only one rule, which is visualized in Fig 6. For the comparison of early RA to other arthritides (undifferentiated arthritis, OA, arthralgia) we selected the intersection of genes differentially expressed in the single-variable comparisons of early RA vs normal, early RA vs arthralgia and early RA vs OA (it would be no gene left when including early RA vs undifferentiated arthritis in the intersection). This resulted in 94 differentially expressed genes. Also for this classification, a PART model is one of the best. It consists of four rules, which are shown in Fig 7. An overview of other model performances is shown in S3 Table. For an overview of single variable importance measured with information gain [51] and reliefF [52] see S4 Table. The selection of genes based on single-variable comparisons as input for generation classification models weakens the validity of the internal cross-fold validation, but is nevertheless more solid than the direct group wise comparison (e.g. just normal vs early RA) without any validation. The genes which were selected for the models are not unknown: LXN is known to be upregulated in early OA [53] and not only as part of the inflammatory response but also influencing the perception of pain [54]; several CXCL genes (chemokines) have been shown to be upregulated at RA, also CXCL8 [11, 12]; MAB21L2 is upregulated especially in OA [55]; about the RP11-* genes less is known as these labels are still their original clone ID [56].

## Discussion

In this analysis, we have added several new views, hints and insights about gene expression in RA. Some of these approaches had been tried on former, limited microarray data in the one or other similar way [18, 19, 57–60]. Compared with these results, it makes sense to to repeat the same or similar types of analyses with recent RNA-seq data. With this recent data, we got several new conclusions. First, the clustering and dimension reduction gives a highly informative and high-level view of RA and related diagnoses; it shows the major difference of the healthy and the unhealthy conditions. Such high-level views are presented in several papers (as in the microarray papers mentioned before); here it is shown with more sensitive RNA-seq data.

### Clustering

The clustering and dimension reduction shows in brief certain high-level differences between the conditions (Fig 1), no differences between men and woman on the highest level (Fig 2), no differences between men and woman at gene cluster level (S12 Fig) and expected vicinity of the conditions by their fold-changes (S6 Fig). OA and establishedRA covers the largest area in the PCA, which might be related to their loose definition, both are samples any time after their first diagnosis (establishedRA at least 12 months after treatment start).

### Overlaps in gene lists

S13 and S14 Figs give a summary of all overlaps between three and four conditions. The amount of linking arcs might be overwhelming at the beginning, but every link can be located with one close look. Venn diagrams are the straightforward choice to visualize overlaps, but they are unsightly with more than 4 or 5 sets, except maybe the famous six-way banana Venn diagram [61]. In the S13 and S14 Figs we have 9 and 12 sets.

### Men and women

We showed with gene expression data that significant differences between men and women exist at least on a detailed view on RA (Fig 4; Fig 2 for the high-level view). The set of genes being three-fold significant (significantly differentially expressed in normal vs. early RA, in men vs women in normal and early RA, but with reversed sign, see Fig 4A) looks a bit out of place for synovial tissue with their term enrichment for muscles (muscle filament and sarcomere), but these are also enriched for actin filament reorganization. It is known that rearrangements in the cytoskeleton are associated with RA [62, 63]. This seems in this dataset specific for earlyRA. At establishedRA several genes related to the immune system are higher expressed. Some of these genes were already reported as sex-biased genes [64], here we show a particular instance. These 'RA-cytoskeleton-genes' and these sex-biased immune system genes are solid points for further investigation, as they might be targets for therapy, responsible for some side effects different in men and women, the effect of different behavior after progression of RA or just be an artifact unknown to now.

### Gene biotypes

In the high-level view of gene biotypes, we see a clear pattern of generally lower gene expression and more genes significantly down-regulated in RA and arthralgia (Fig 5). This give rise to the hypothesis that the miRNA diagnostic marker(s) for RA—what many are looking for [29, 65, 66]—might be negative ones (that means the lack of certain miRNAs would point to RA), although it might be confused with arthralgia. The rRNA is worth extra attention as it is the major difference between arthralgia and the rest. rRNAs of mitochondria are less expressed in RA conditions and arthralgia, likely because of the hypoxic microenvironment [67, 68], but also possible associated with general exhaustion [69, 70].

### Classification models

For the simplest distinction between RA and other conditions, we provide two classification models. The genes used in the models might not be causative or functionally most related, but are a minimal set of genes to classify the data, which classification in that way is also significant at 10-fold cross-validation. The pre-selection of genes based on intersecting single-variable comparisons is needed for escaping the curse of dimensionality for multivariable classification methods. This pre-selection has some limitations: it has itself no internal validation and the gene sets from the single-variable comparisons are differently solid, as there are conditions with different sample sizes (smallest: 6 samples of UA and 10 of arthralgia). This increases the chance to lose the 'best' (= most likely causative) predictors and getting instead the most correlated (to the 'best' predictors) variables in the model. This pre-selection weakens the validity of the internal cross-fold validation. The used classification method (PART [50], a tree learner based rule generator) is likely over-simplifying RA. For final assessments of the particular—potential causal—functions of the selected predictors, dedicated wet lab experiments are needed. The presented classification models are only intended for a distinction between RA and other conditions as simple as possible based on gene expression.

### Previous microarray studies

Many RNA-seq data is already published for RA, as used in this article; more sample data is still available from microarrays. Single cell sequencing RNA-seq samples are catching up in number and of course depth, but previous microarray data are still a large source to compare with. The comparison of the collected RNAseq data with suitable previous microarray studies shows expected results (S1 Text, especially S15 Fig). The fact that the relative overlap of the up-regulated genes is always higher than of the down-regulated genes could point to a bias of seen importance (= more solid annotation, as this have changed over time) of these up-regulated genes or to a higher biological importance of these genes (as they are more overlapping in independent studies). Overall, it seems important to use RNA-seq data instead of microarray data for the transcriptome, to use the very same gene annotation and to process the data in the very same way. In such collections are likely still plenty of hidden insights.

## Material and methods

### Data collection

We have combined and compared 236 RNA-seq synovial biopsy samples from the papers of Walsh et al. and Guo et al. [15, 16] and microarray data from the papers of Liu et al., Teixeira et al., Niu et al. and Yoshida et al. [18, 58–60] in this study. This RNA-seq sample collection was chosen, because it is consistent, large and there are open questions for which insights or at least hints are in this data. The main data are the 236 RNA-seq samples, the microarray data are only used for showing the overlaps with the main data.

The samples of Teixeira et al. and Niu et al. are from peripheral blood (Peripheral Blood Mononuclear Cells—PBMCs), whereas all other samples are from synovial tissue. RNA-seq data were downloaded from the Sequence Read Archive (SRA) [71] (see S1 Table for accession identifiers and number of samples per diagnosis), microarray and clinical data were taken as presented in the original papers. The raw RNA-seq data were processed as described in the next section, the clinical information was taken as provided in the source papers [15, 16], where ‘normal’ refers to healthy patients, ‘arthralgia’ refers to a population based on this symptom, rather than a specific diagnosis, ‘earlyRA’ means treatment naïve RA within 12 months of first diagnosis, ‘establishedRA' means treatment experienced RA of >12 months disease duration, ‘RAtripleDMARD' means RA about 6 months after treatment initiation with methotrexate, sulfasalazine and hydroxychloroquine, finally ‘OA’ means osteoarthritis and ‘UA’ means undifferentiated arthritis. 'establishedRA' and ‘RAtripleDMARD' are both patients under ongoing treatment, where the latter was a specific treatment and the sample after a certain time (6 months after treatment start) and the former are patients at/after any treatment after a longer period of time (>12 months after treatment start) [16]. Undifferentiated arthritis is defined as clinical signs of synovitis, but failing to meet the 2010 American College of Rheumatology criteria for RA [16]. An ‘F’ or an ‘M’ appended to a label means the subset of female and male patients.

### RNA-seq—primary data processing

Reads were mapped onto the human reference genome release hg38 (GRCh38) [72] with Ensembl transcript annotation version 87 [48] using Tophat version 2.1.1 [73] with Bowtie version 2.2.9 [74]. Reads were counted with featureCounts [75] and gene expression values (reads per kilobase exon per million mapped reads (RPKM)) were calculated with Cufflinks version 2.2 [76]. The differential expression between two sample groups was calculated with edgeR [77]. The filtering for differentially expressed genes is for p-value of 0.05 (FWER corrected) and minimal fold-change of 2. In the more specific analyses for single genes, for the differences in men and woman and for classification models, (healthy) age-related genes are removed. This is because the sampled healthy subjects are in average quite younger than the subjects with different arthritis conditions and at comparisons between them age-related genes are expected to be significantly different. Age-related genes are taken from Yang et. al. [78]. We performed also comparisons of gene expression between groups adjusted for age. At the most changing adjustment in the comparison between healthy subjects and early RA (an average age of 35.2 vs. 55.9), we realized that many genes well known for RA are filtered (as CCL19 [79], CCL22 [80], CCR6 [81], CD6 [82], CDH11 [83], IFIT1B (as a paralog to IFIT1 [84]), IL26 [85], IL2RB [86], MMP10 [87], MMP12 [88], MMP8 [89] and MMP9 [90]). Similar worrying are the overlaps between unique DEGs in the comparison unadjusted and adjusted by age with the external age-related genes (as used for filtering from Yang et al. [78]), we see even a higher overlap between age-adjusted DEGs (healthy vs early RA) and the external age-related genes. Given that, we used the comparison without adjustment for further analyses. Age-adjusted comparisons are available in the Supplementary Archive.

### Clustering and dimension reduction

The RPKM values per gene were the input for clustering. The standard R [91] functions were used for PCA and hierarchical clustering, as well as the interfaces of the visualization libraries (described in section visualization). For other dimension reduction methods the Matlab Toolbox for Dimensionality Reduction was used [92].

### Co-expression analysis

For clustering genes into modules based on their expression profiles over all conditions, we used the expression of the transcript with the highest expression per gene. We grouped samples into conditions by choosing the median expression per gene and used this information as input for the Weighted Gene Co-expression Network Analysis (WGCNA) method [93]. Genes were kept only if the total cumulative RPKM over all samples was more than 10 and when exceeding the standard deviation of 0.5 along all conditions.

### Gene enrichment analyses

For Gene Ontology (GO) [37] enrichment analysis of a gene set, GOstats version 2.46.0 [94] was used with default parameters, except the parameter 'conditional', which was set to TRUE (which removes genes from significant terms deeper in the hierarchy). For the detection of enriched KEGG [38] and REACTOME [39] terms geneSCF version 1.1 [95] was used. All complete lists are available in the Supplementary Archive (tables/). For having a diagram of the GO (BP) terms fitting onto a single page (in Fig 3), we used REVIGO [96] for reducing overlapping terms with an allowed similarity threshold of 0.4 and the Cytoscape [97] plugin EnrichmentMap [98] for visualization with a threshold of 10^−6^ for the raw p-values and an edge similarity threshold of 0.5.

### Visualization

For the visualization of clusters, distributions, overlaps, correlations and ratios we used the following R packages: ape [99], vioplot [100], dplyr [101], ggplot2 [102], ggrepel [103], FactoMineR [104], factoextra [105] and WGCNA [93]. Additionally, we used the tools Cytoscape [97] and Circos [106].

### Machine learning for classification models

For the following classification methods, the reference implementation in WEKA [107] was used: C4.5 [32] (implemented as J48), PART [50], Alternating Decision Trees [108], naive Bayes [109], SMO [110]. The importance of variables was measured with their information gain [51] and reliefF [52] as implemented in WEKA.

## Supporting information

S1 Fig The first two principal components of the PCA based on the RPKMs of the coding genes. The areas are the convex hulls of the conditions. The largest point of one color depicts the center of a hull. Number of samples: 22 OA, 10 arthralgia, 57 earlyRA, 95 longRA, 27 normal, 19 RApost and 6 UnArth.
(TIF)

S2 Fig Conformal Eigenmaps (CCA) based on the RPKMs of the coding genes. Number of samples: 22 OA, 10 arthralgia, 57 earlyRA, 95 longRA, 27 normal, 19 RApost and 6 UnArth.
(TIF)

S3 Fig Sammon mapping based on the RPKMs of the coding genes. Number of samples: 22 OA, 10 arthralgia, 57 earlyRA, 95 longRA, 27 normal, 19 RApost and 6 UnArth.
(TIF)

S4 Fig Multidimensional scaling (MDS) based on the RPKMs of the coding genes. Number of samples: 22 OA, 10 arthralgia, 57 earlyRA, 95 longRA, 27 normal, 19 RApost and 6 UnArth.
(TIF)

S5 Fig The first ten principal components of the PCA considering RPKMs of the coding genes. The areas are the convex hull of the condition. The largest point of one color depicts the centers of the hull. Only those conditions are shown where more than ten samples were available for male and female individuals. Number of samples: 33 earlyRAF, 24 earlyRAM, 73 establishedRAF, 22 establishedRAM, 13 NormalF, 14 NormalM.
(PDF)

S6 Fig Neighbor joining tree based on the log fold-changes of significantly different genes (significant in any comparison). Origin for the fold-changes is normal/healthy (normal/healthy is the 0-vector). The x-axis is based on the Manhattan distance of significant fold-changes. The distance might be meaningless as an absolute value, but informative as relative distance.
(TIF)

S7 Fig Neighbor joining tree based on the log fold-changes of significantly different miRNA genes (significant in any comparison). Origin for the fold-changes is normal/healthy (normal/healthy is the 0-vector). The x-axis is the distance. The distance might be meaningless as an absolute value, but informative as relative distance.
(TIF)

S8 Fig Hierarchical clustering based on the log fold-changes of significantly different genes (significant in any comparison). Origin for the fold-changes is normal/healthy (normal/healthy is the 0-vector). The x-axis is the distance. The distance might be meaningless as an absolute value, but informative as relative distance.
(TIF)

S9 Fig Hierarchical clustering based on the log fold-changes of significantly different miRNA genes (significant in any comparison). Origin for the fold-changes is normal/healthy (normal/healthy is the 0-vector). The x-axis is the distance. The distance might be meaningless as an absolute value, but informative as relative distance.
(TIF)

S10 Fig Log2 fold-changes of gene expression between men and women in established/long RA and normal condition. Only genes are shown which are significantly differentially expressed in men and women. The size and shape shows the significance in differences of men and women: the large circles are genes significantly differentially expressed between the sexes in established/long RA and normal condition, these genes are also labelled. Small squares mean a significant difference between men and woman only in established/long RA, small diamonds mean a significant difference only between healthy men and woman. The color represents the significance of the difference in expression between normal and established/long RA.
(TIF)

S11 Fig Log2 fold-changes of gene expression between men and women in OA and normal condition. Only genes are shown which are significantly differentially expressed in men and women. The size and shape shows the significance in differences of men and women: the large circles are genes significantly differentially expressed between the sexes in OA and normal condition. Small squares mean a significant difference between men and woman only in OA, small diamonds mean a significant difference only between healthy men and woman. The color represents the significance of the difference in expression between normal and OA.
(TIF)

S12 Fig Gene clusters. Genes were clustered into modules of co-expression and their module eigengenes (ME) normalized expressions are shown here for all conditions. In panel B the patients are split in male/female and diagnosis, in panel A the average value of male and female is shown. The color and the size of the dots depict the first principal component per module using the scaled expression of the respective genes of a module over all conditions. Parts of panel B should be seen with caution as some groups have a very small sample size (arthralgiaM and UAM). Pairwise significant differences of conditions within modules are in S5 Table. These are summarized in panel B as color-code: purple rows and columns are not significantly different to any condition in any module, gray cells are not significantly different to any condition within the particular module. Number of samples: 8 arthralgiaF, 2 arthralgiaM, 13 normalF, 14 normalM, 13 OAF, 9 OAM, 19 RAtripleDMARD, 5 UAF, 1 UAM, 33 earlyRAF, 24 earlyRAM, 73 establishedRAF and 22 establishedRAM.
(TIF)

S13 Fig The amount and the overlaps of up- and down-regulated genes for different RA conditions. The base condition is normal, so the label ‘earlyRA’ means normal compared with early RA. Up-regulated fractions are shown in green, down-regulated fractions are shown in red; gray are fractions of genes which are not significantly differentially expressed. The full set is the union of significantly differentially expression genes in all comparisons. The colors of the arc connections are dependent on what they are connecting. Number of samples: 57 earlyRA, 95 establishedRA, 27 normal and 19 RAtripleDMARD.
(TIF)

S14 Fig The amount and the overlaps of up- and down-regulated genes for early RA, OA, arthralgia and undifferentiated arthritis. The base condition is normal, so the label ‘earlyRA’ means normal compared with early RA. Up-regulated fractions are shown in green, down-regulated fractions in red; in gray are fractions of genes which are not significantly differentially expressed. The full set is the union of significantly differentially expression genes in all comparisons in this Fig. The colors of the arc connections are dependent on what they are connecting. Number of samples: 10 arthralgia, 57 earlyRA, 27 normal, 22 OA and 6 UA.
(TIF)

S15 Fig Comparison of significantly differentially expressed genes. The base state of genes is the normal condition, except for ‘OA->RA’ where it is OA (base state is the first one in ‘condition <one> compared to condition <two>‘). The origins of the different sources or sets (the 'Set1' to 'Set5') are listed in Table B in S1 Text.
(TIF)

S1 Table Accession identifiers (SRA database) and number of samples per diagnosis. (XLS)

S2 Table The average fold-changes and counts of genes in certain comparisons when genes are grouped according to their biotype. (XLS)

S3 Table Performance of different ML-methods for the classification Normal vs. earlyRA; weka is used. (XLS)

S4 Table The importance of variables for the classifications Normal vs. earlyRA and earlyRA vs. notRAarth; information gain and reliefF is used from weka. (XLS)

S5 Table Pairwise significant differences of conditions within modules of the Weighted gene correlation network analysis (WGCNA). The differences were tested with a Wilcoxon test comparing the eigengene values of the samples of two conditions. The 'significant?' column is on the Bonferroni-corrected p-value. Each combination is twice in the table (e.g. NormalF vs. earlyRAF and earlyRAF vs. NormalF are the same).
(XLS)

S1 Text Relocated supporting text. Contains the sections 'Clustering of genes', 'Overlaps in differentially expressed genes between clinical conditions' and 'Comparisons with other studies'.
(DOCX)
