# Supplementary figures and images for: Analysis of gene expression in rheumatoid arthritis and related conditions offers insights into sex-bias, gene biotypes and co-expression patterns
Source: PLoS One. 2019 Jul 25;14(7):e0219698. doi: 10.1371/journal.pone.0219698 (PMC6657850; doi:10.1371/journal.pone.0219698)

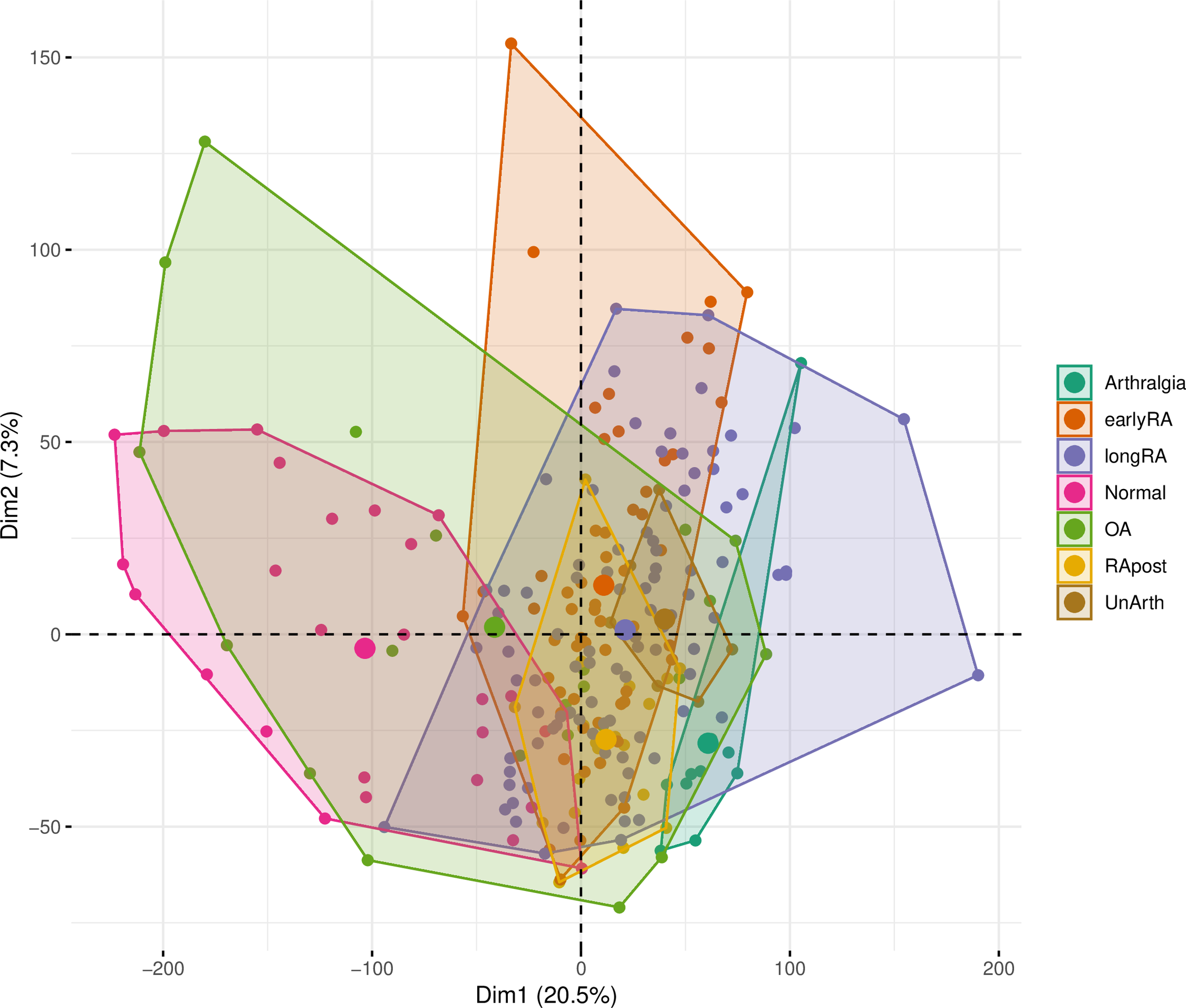

Supplement: S1 Fig — The areas are the convex hulls of the conditions. The largest point of one color depicts the center of a hull. Number of samples: 22 OA, 10 arthralgia, 57 earlyRA, 95 longRA, 27 normal, 19 RApost and 6 UnArth. (TIF) [file pone.0219698.s001.tif]

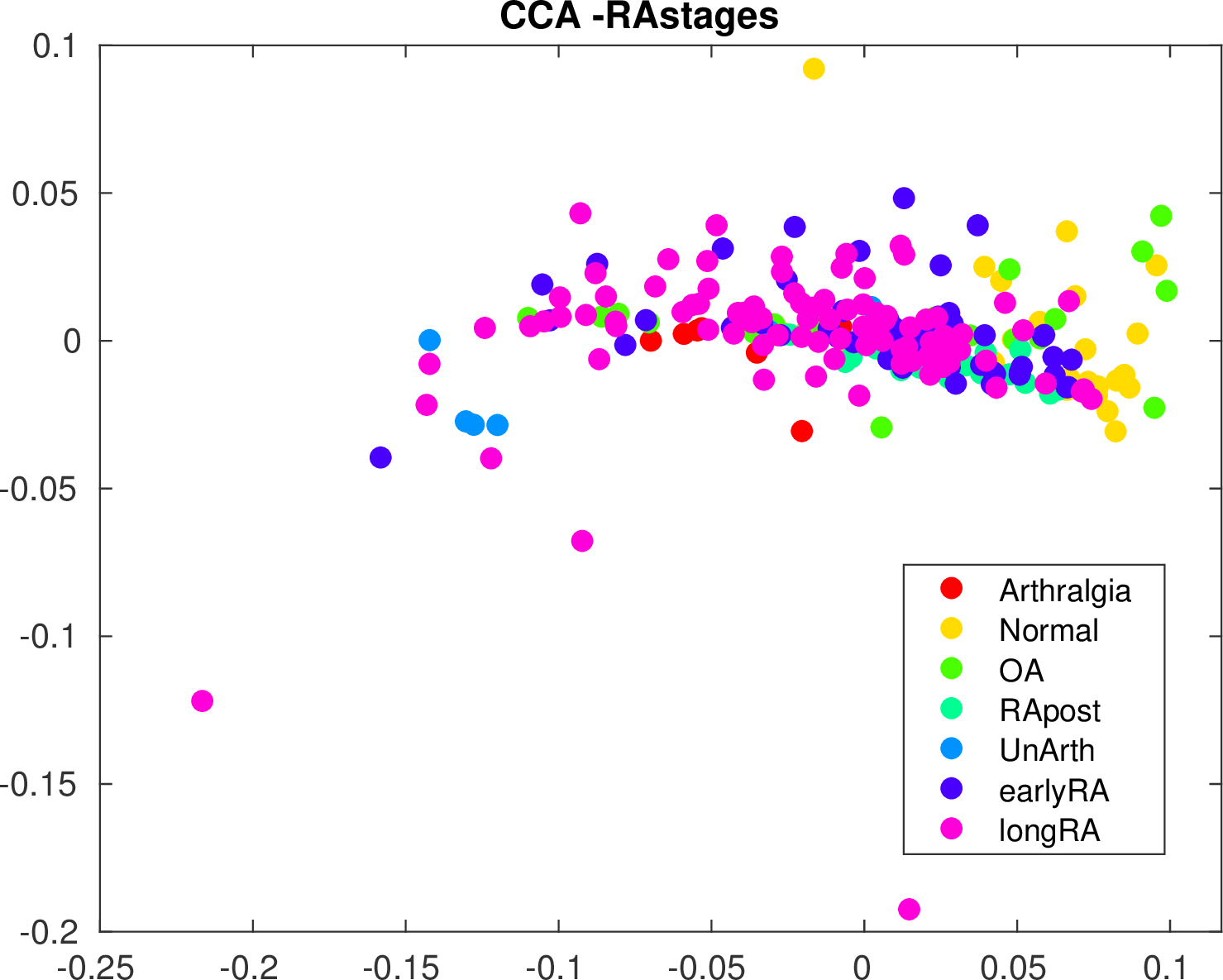

Supplement: S2 Fig — Number of samples: 22 OA, 10 arthralgia, 57 earlyRA, 95 longRA, 27 normal, 19 RApost and 6 UnArth. (TIF) [file pone.0219698.s002.tif]

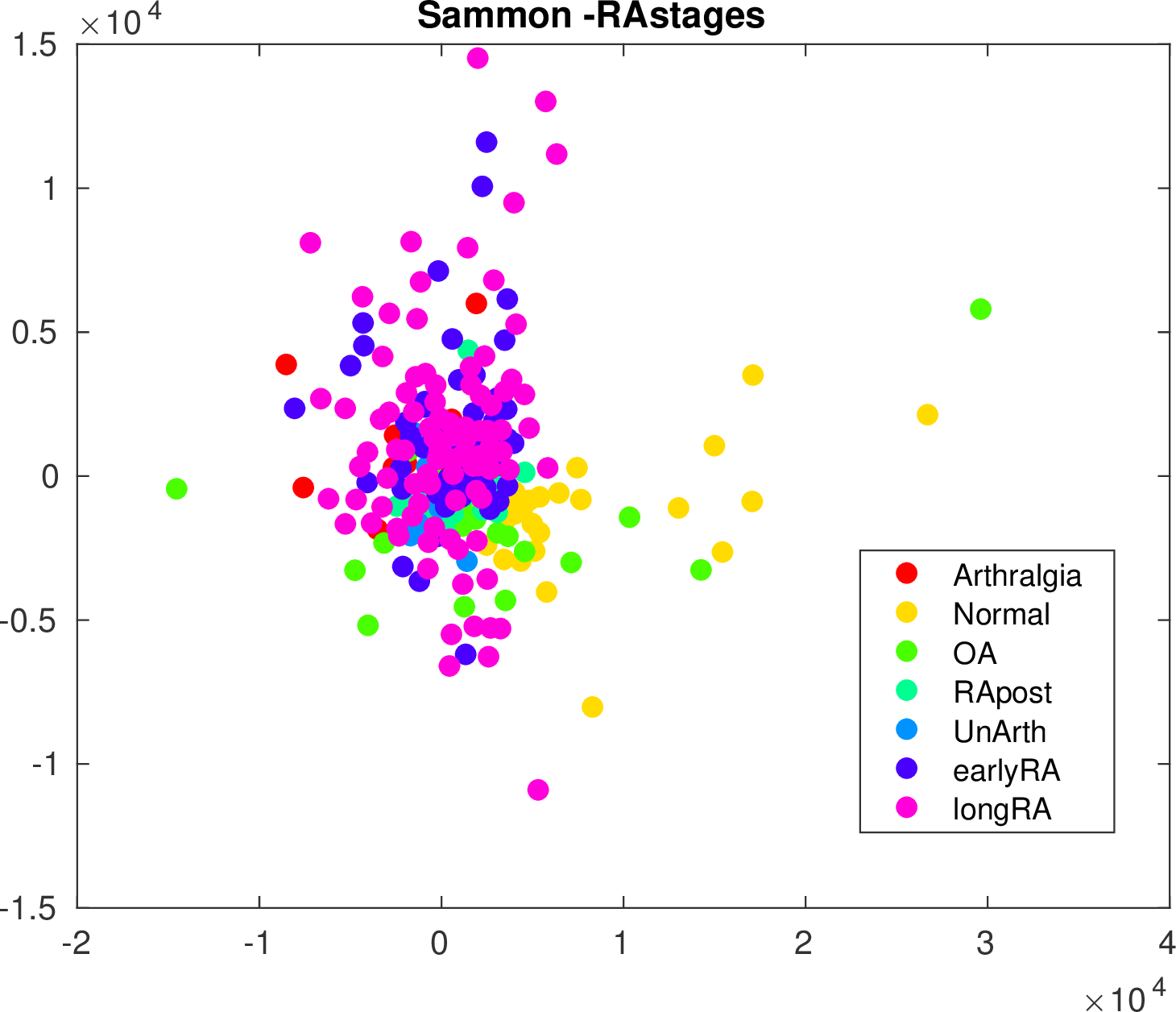

Supplement: S3 Fig — Number of samples: 22 OA, 10 arthralgia, 57 earlyRA, 95 longRA, 27 normal, 19 RApost and 6 UnArth. (TIF) [file pone.0219698.s003.tif]

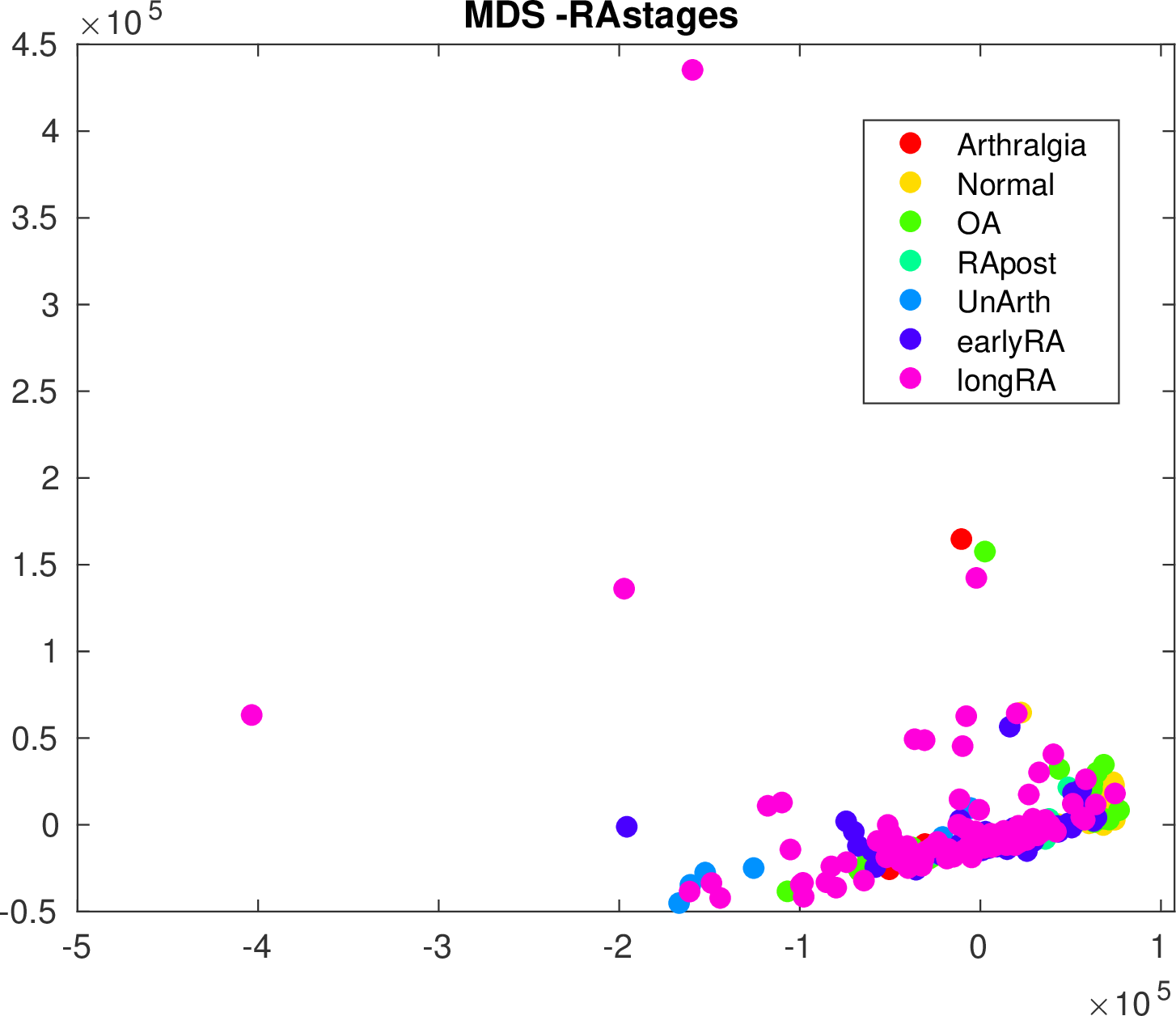

Supplement: S4 Fig — Number of samples: 22 OA, 10 arthralgia, 57 earlyRA, 95 longRA, 27 normal, 19 RApost and 6 UnArth. (TIF) [file pone.0219698.s004.tif]

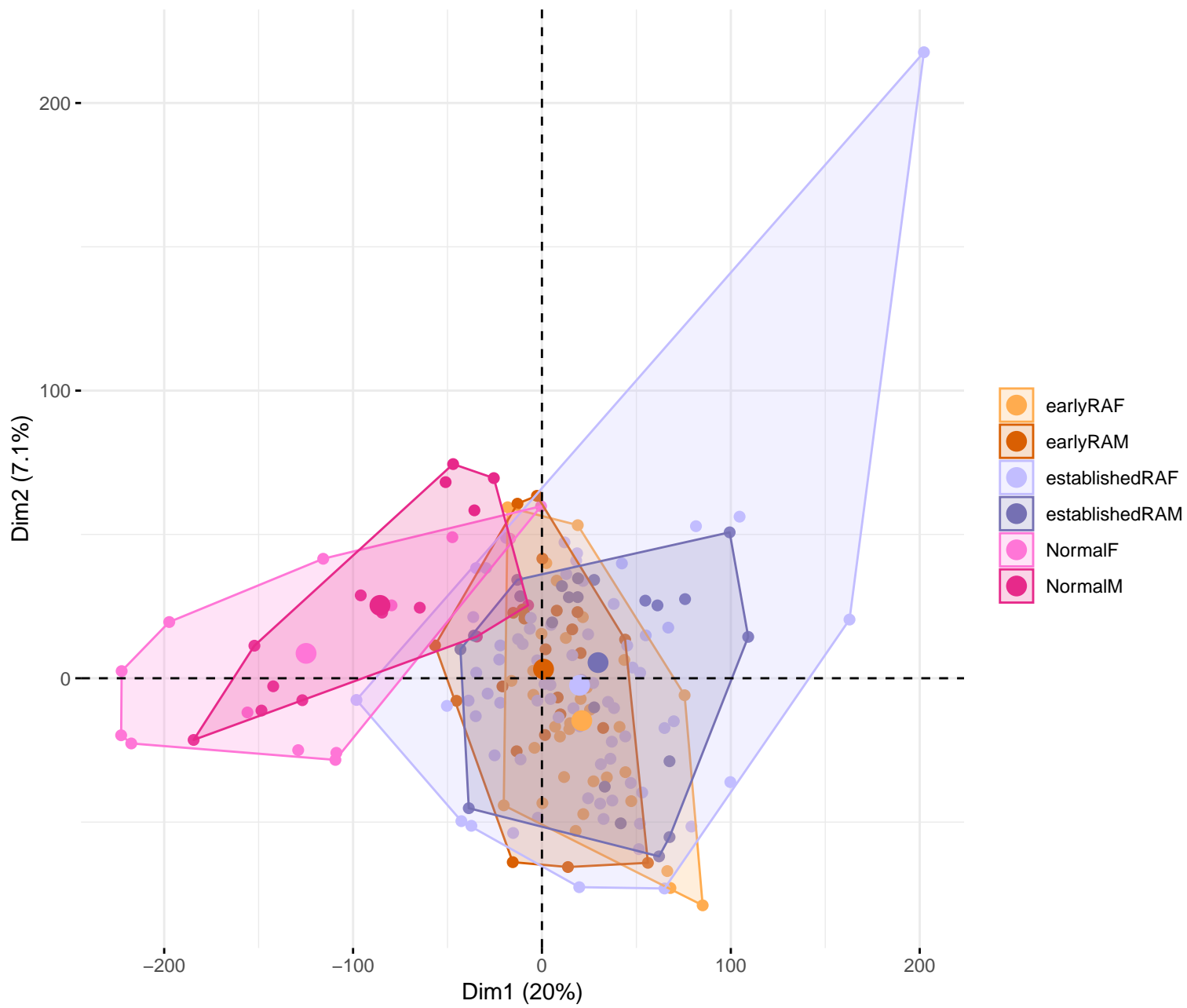

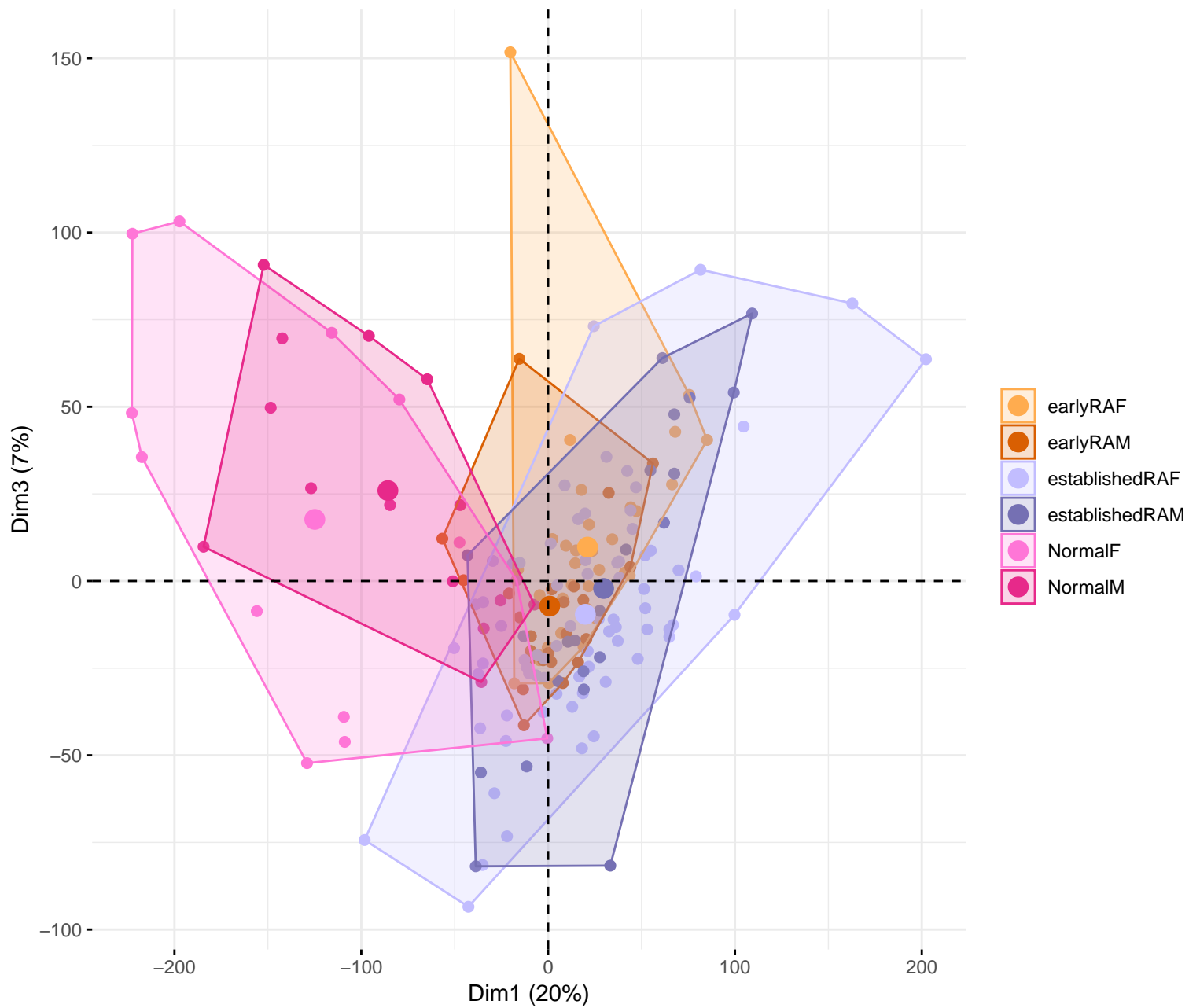

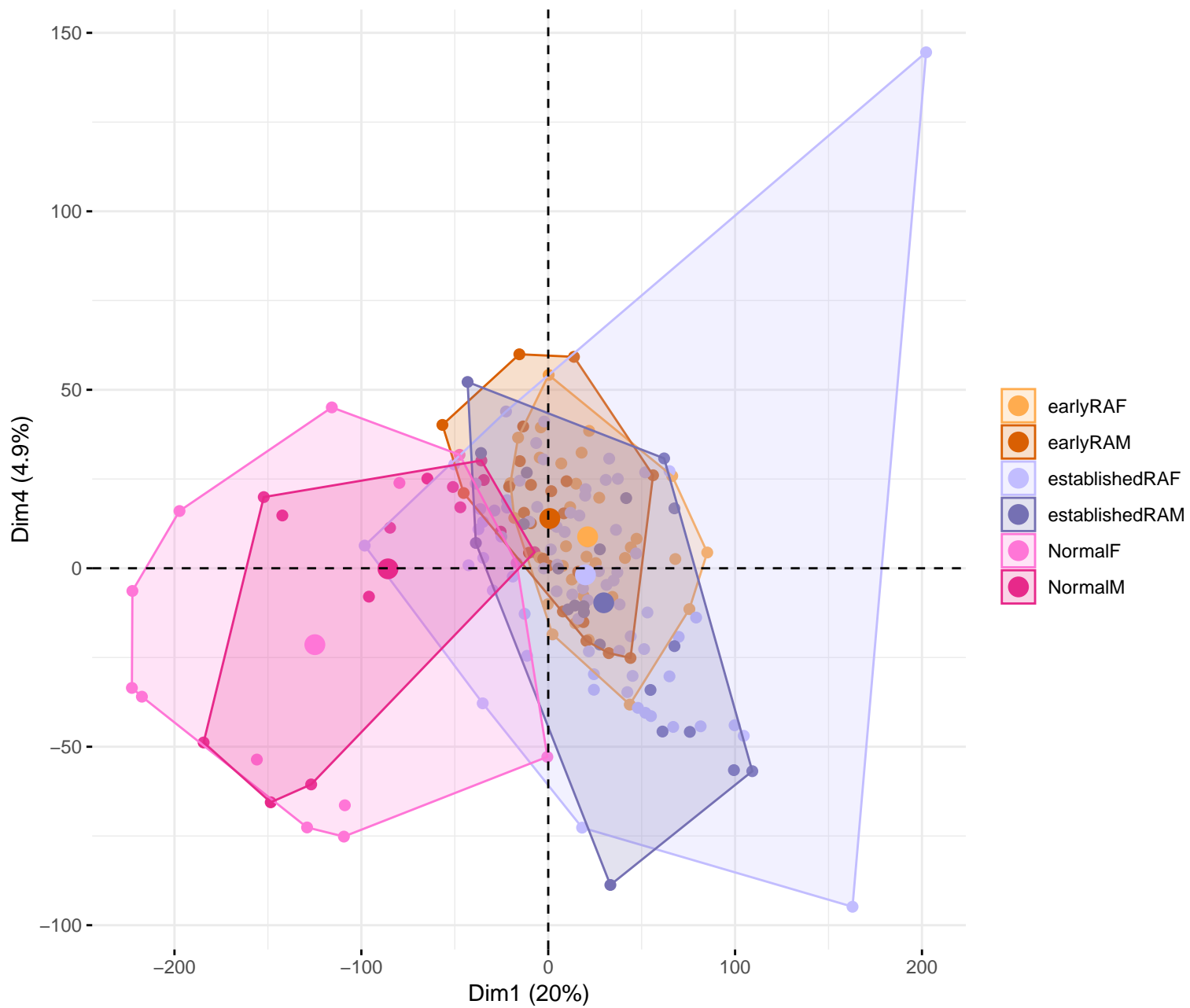

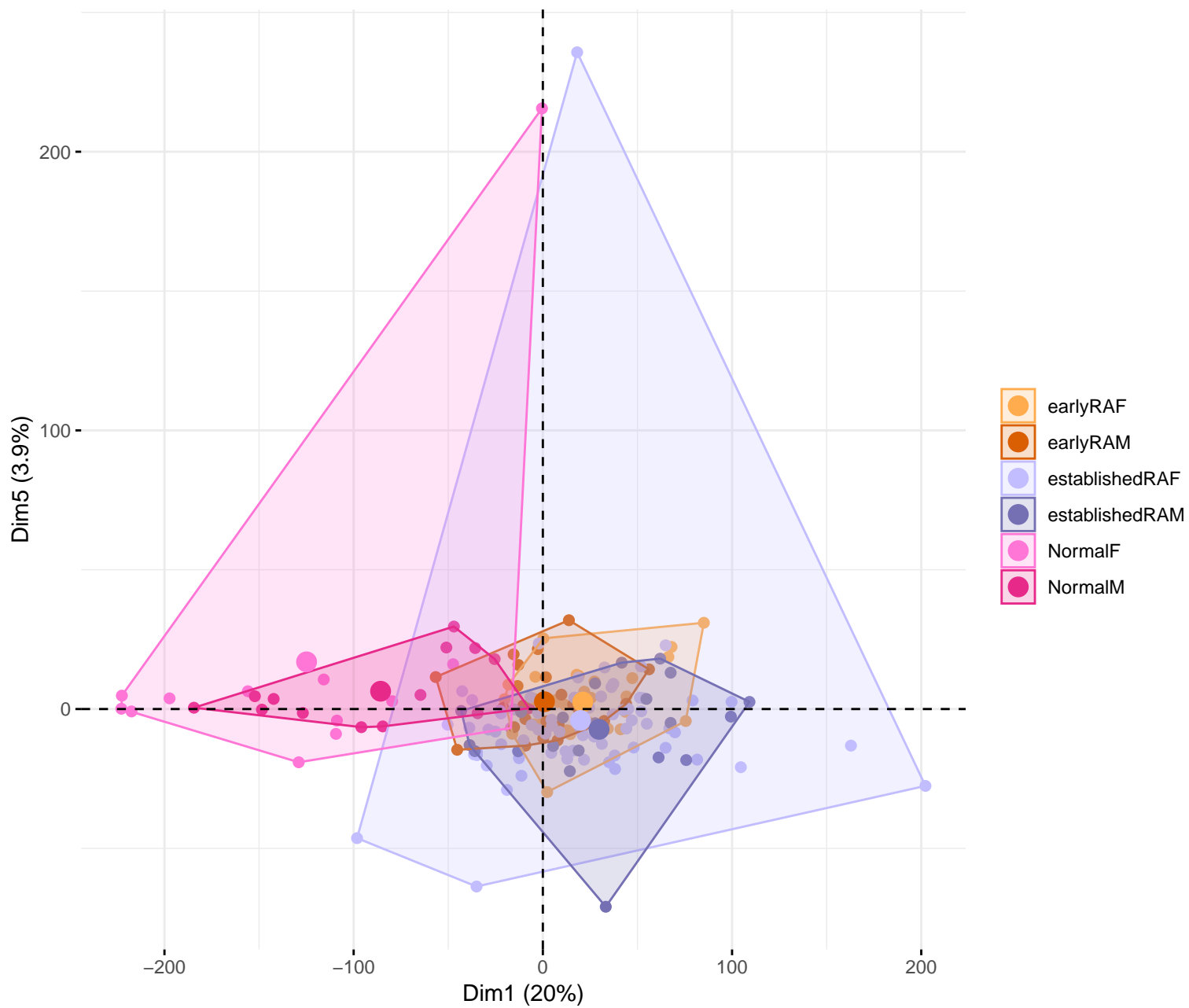

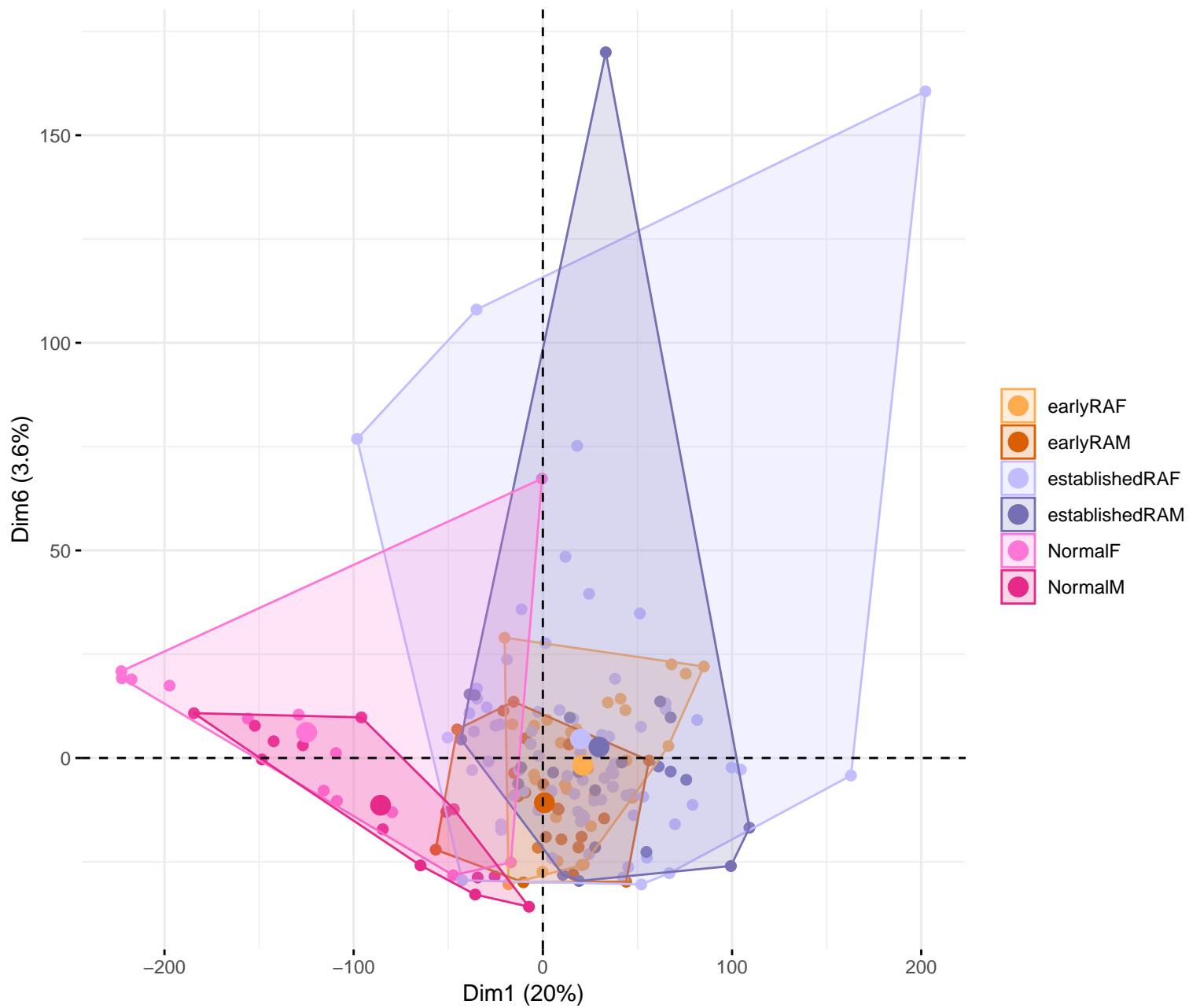

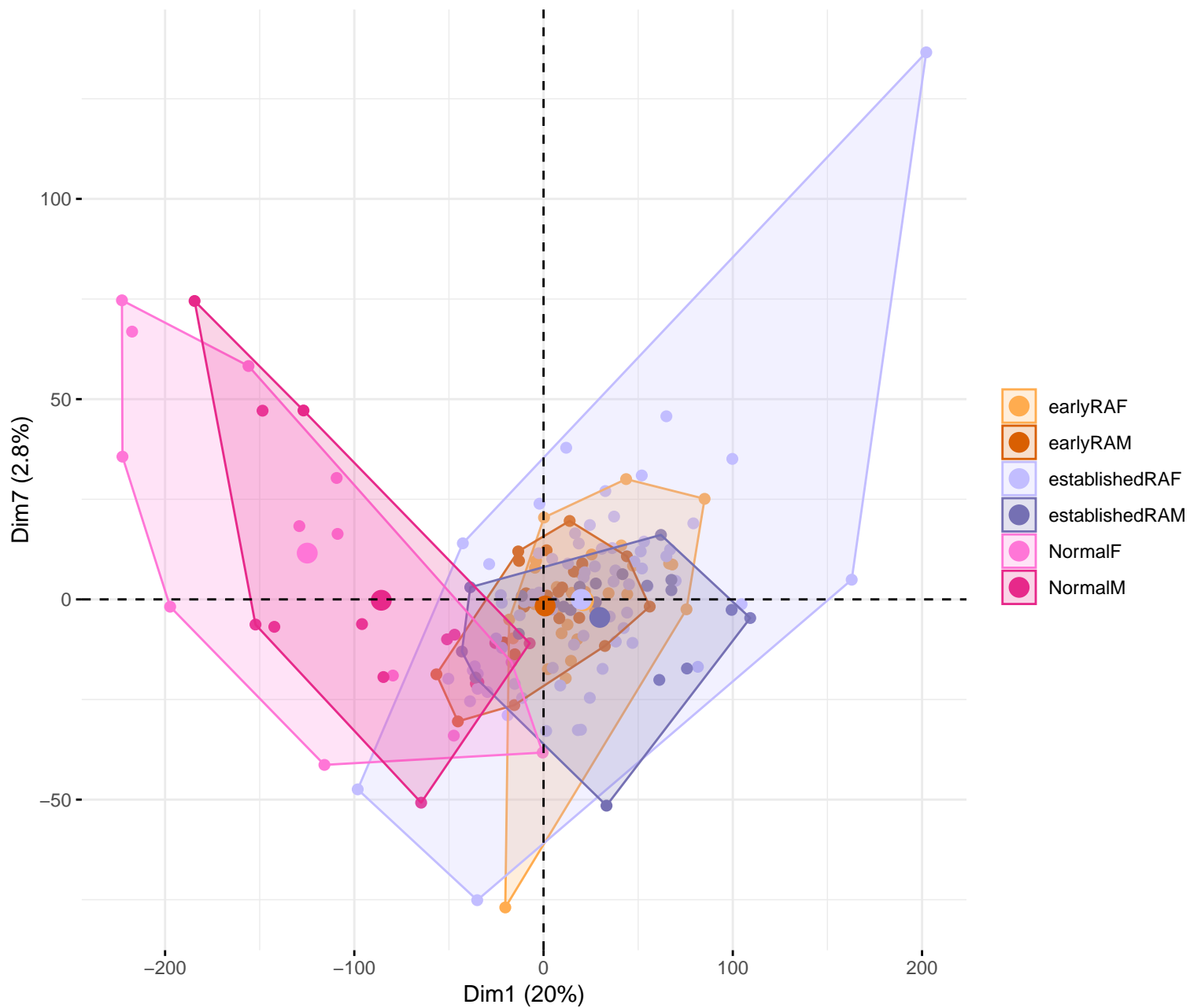

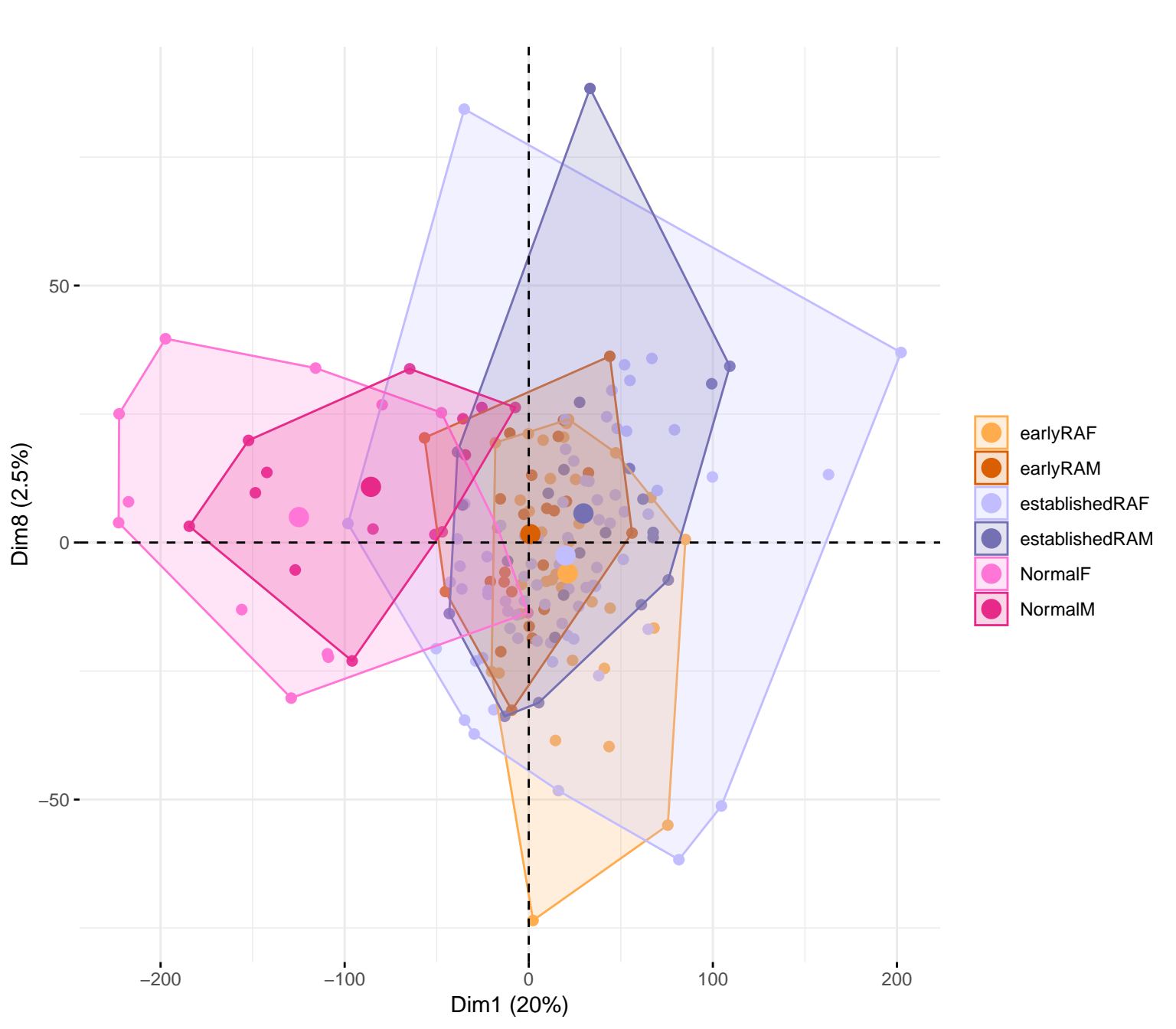

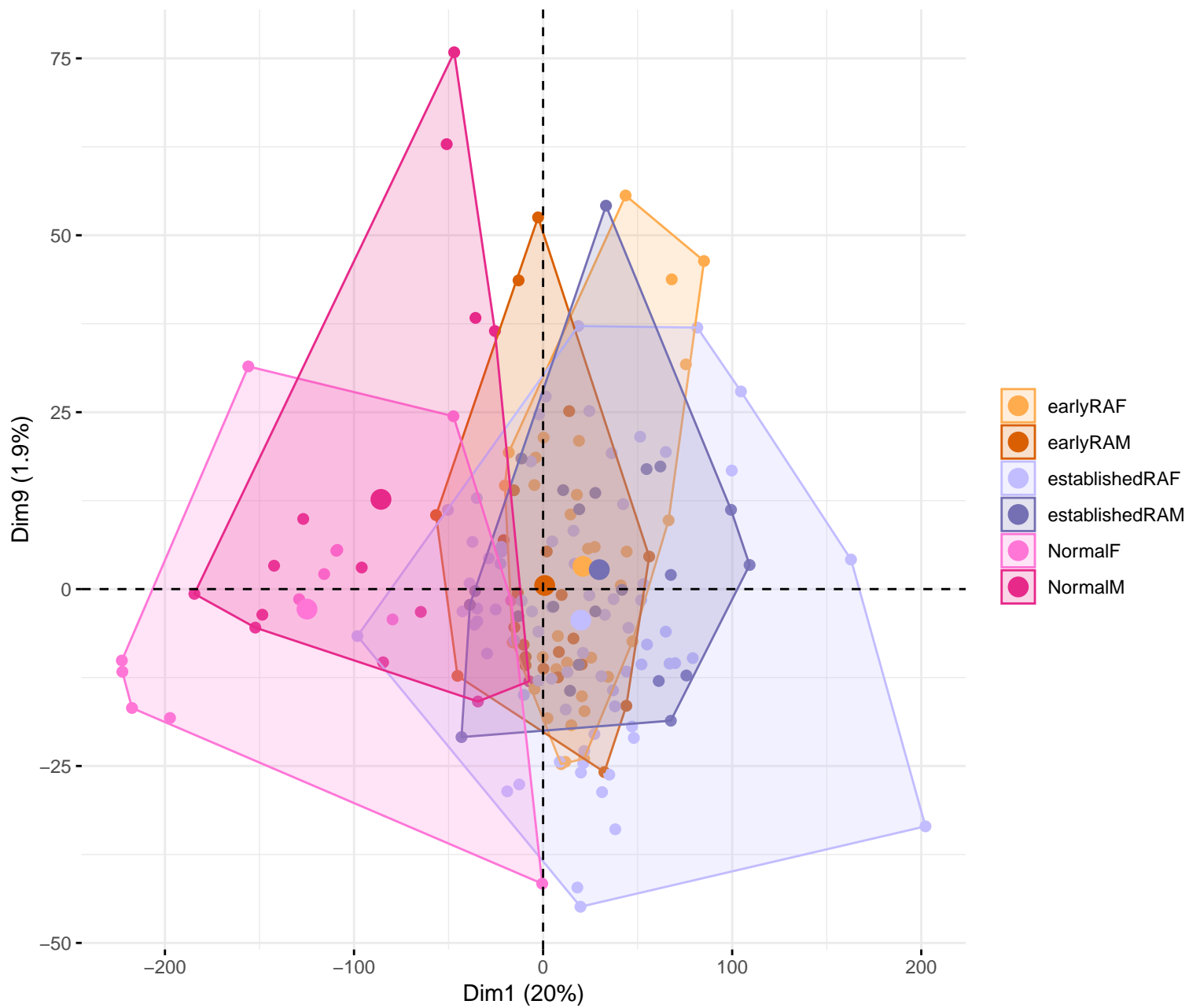

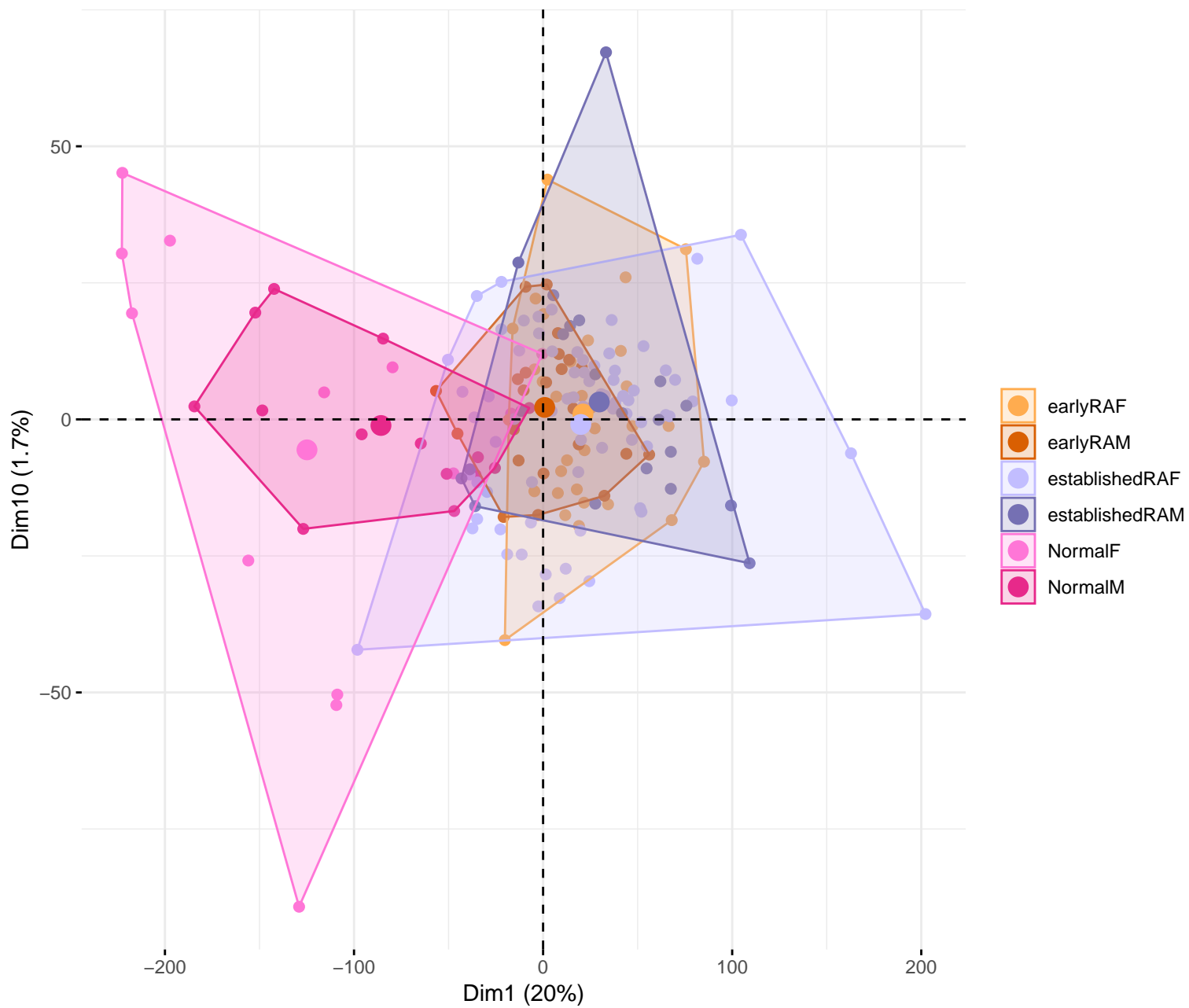

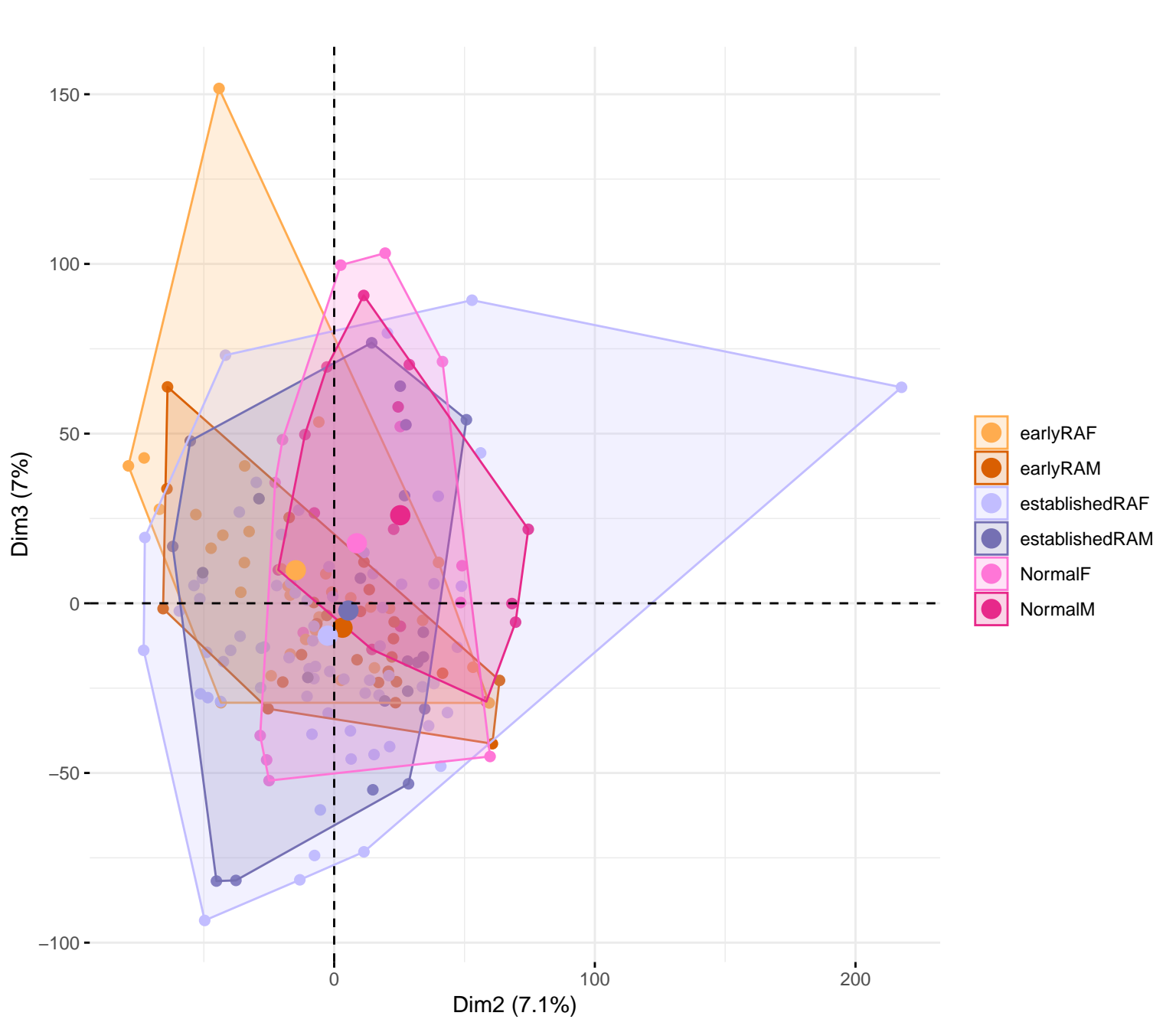

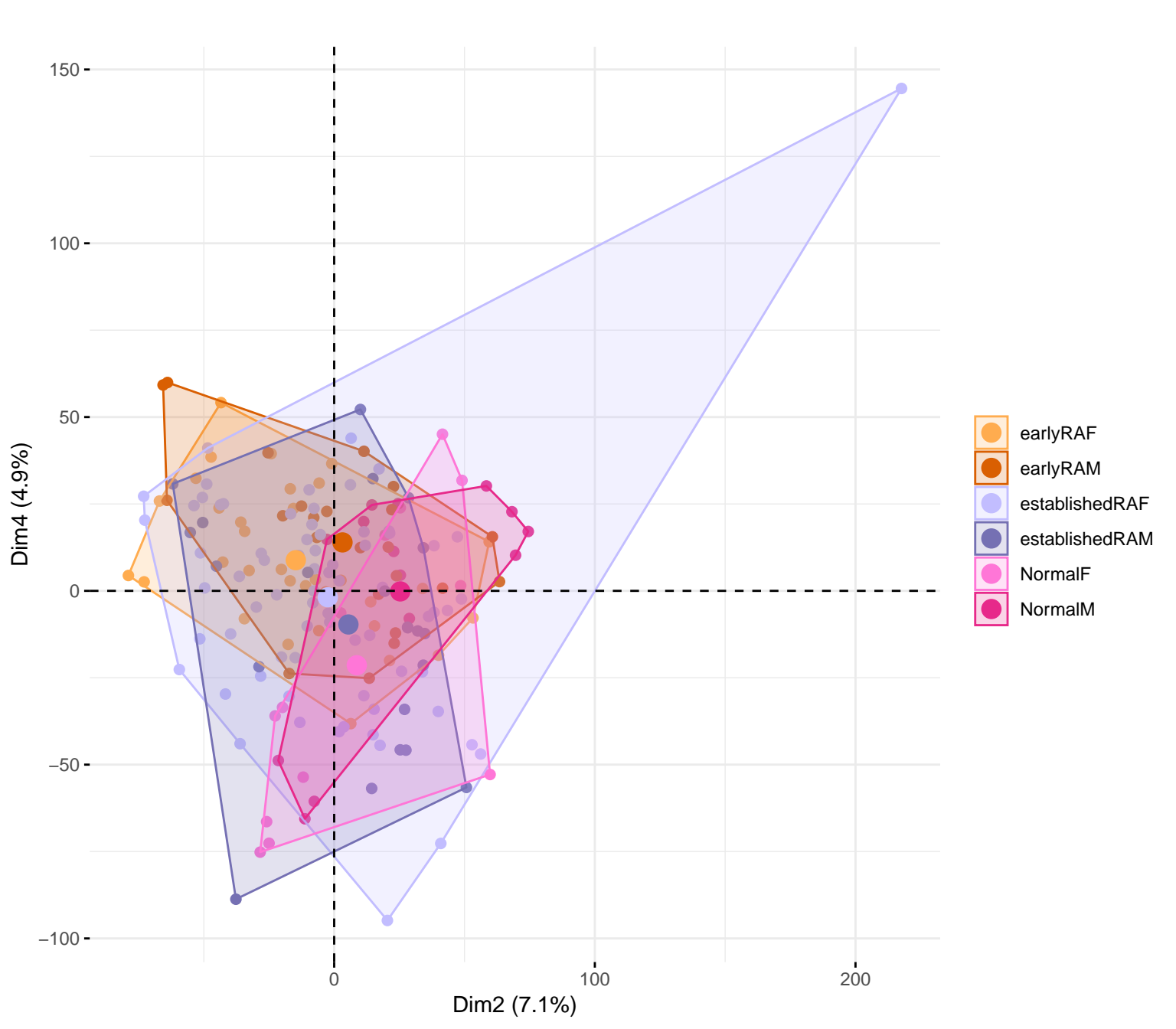

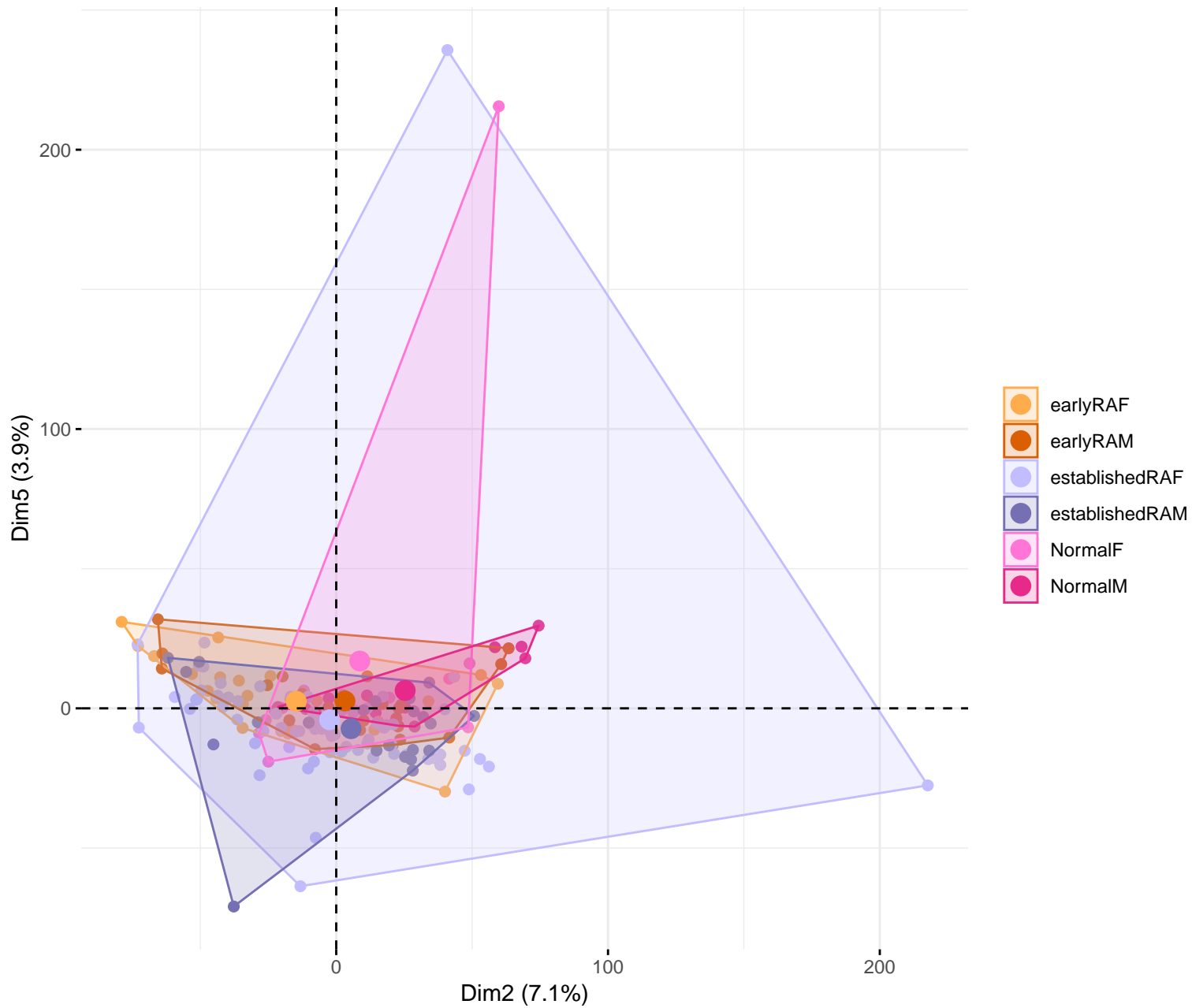

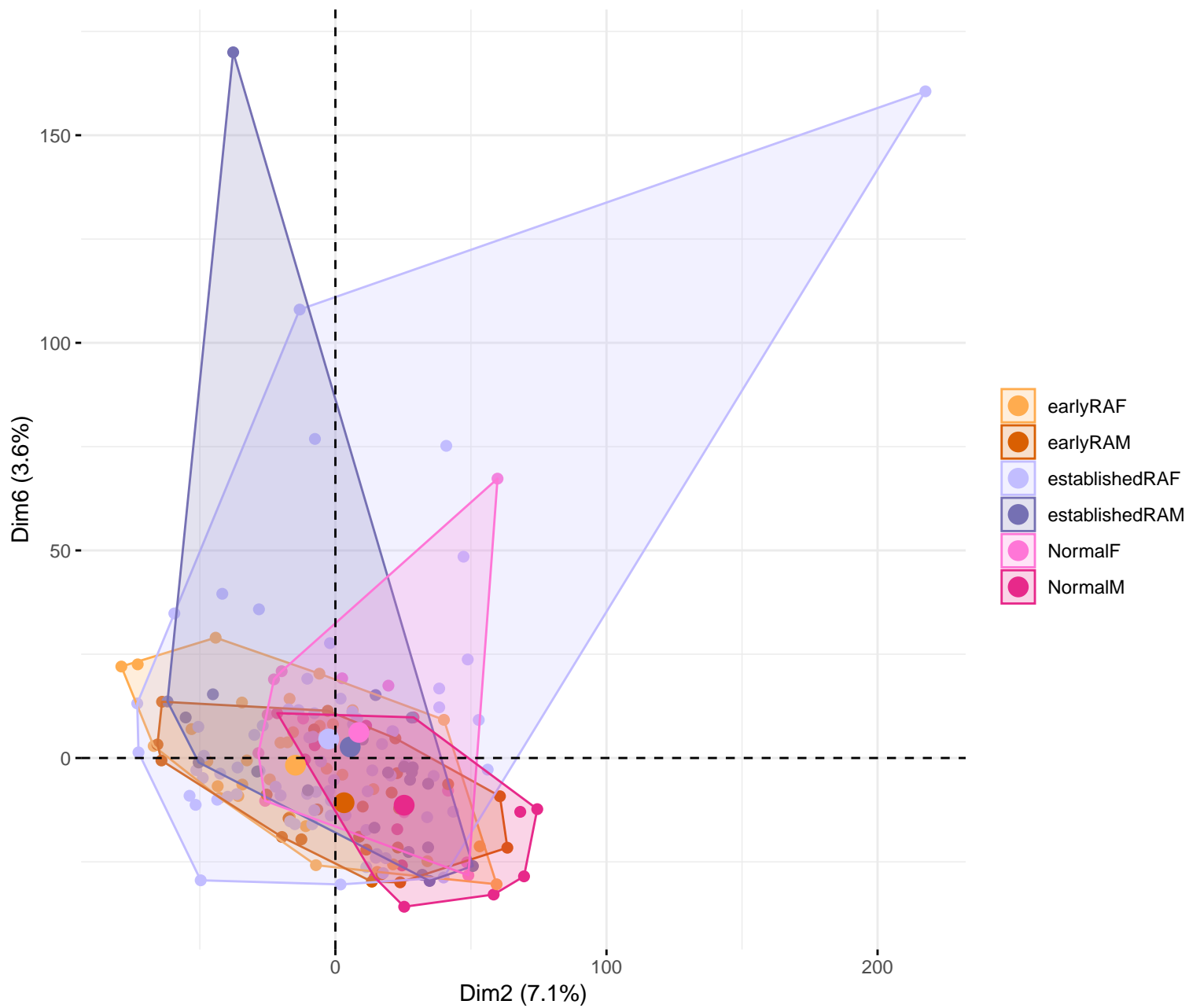

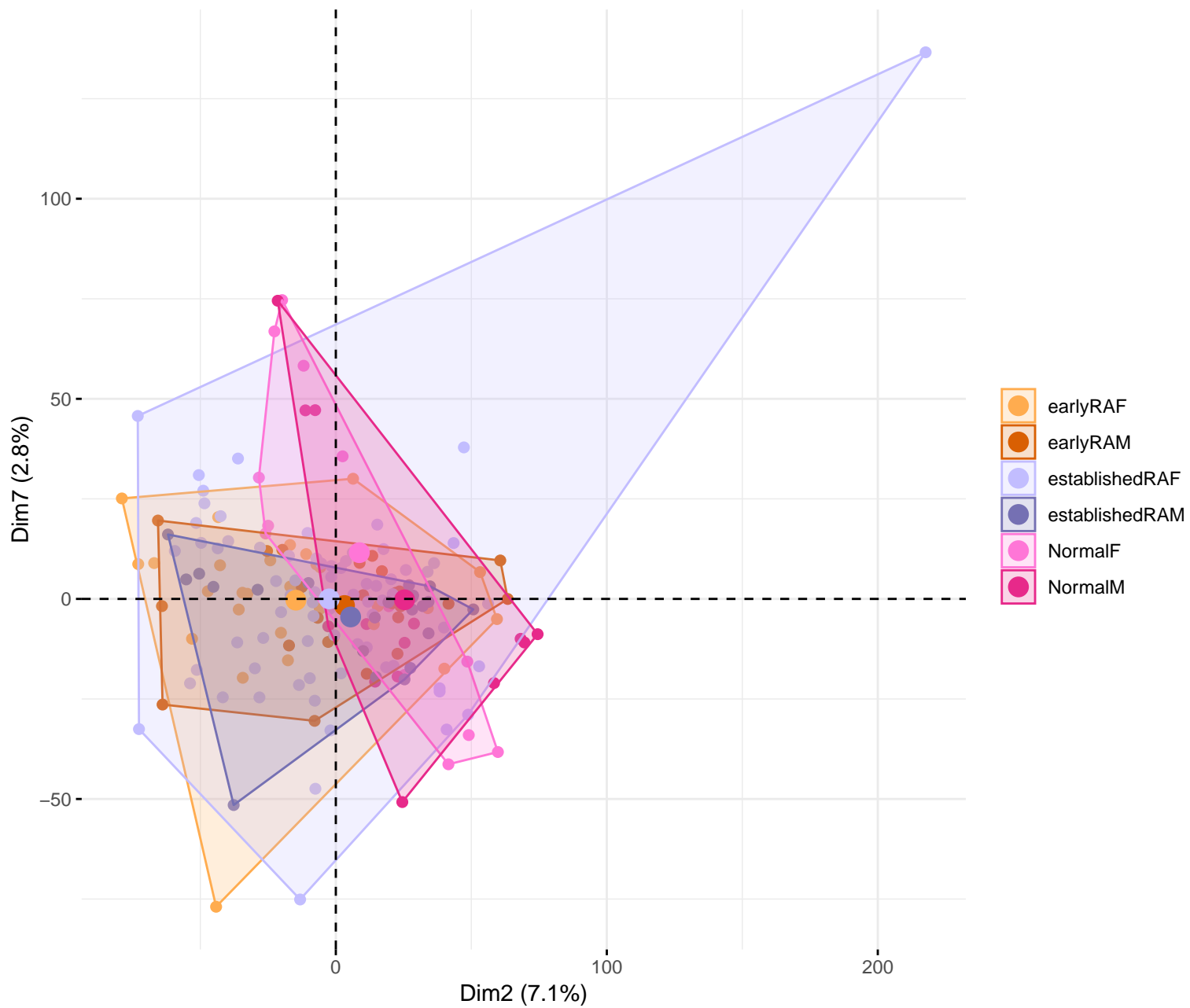

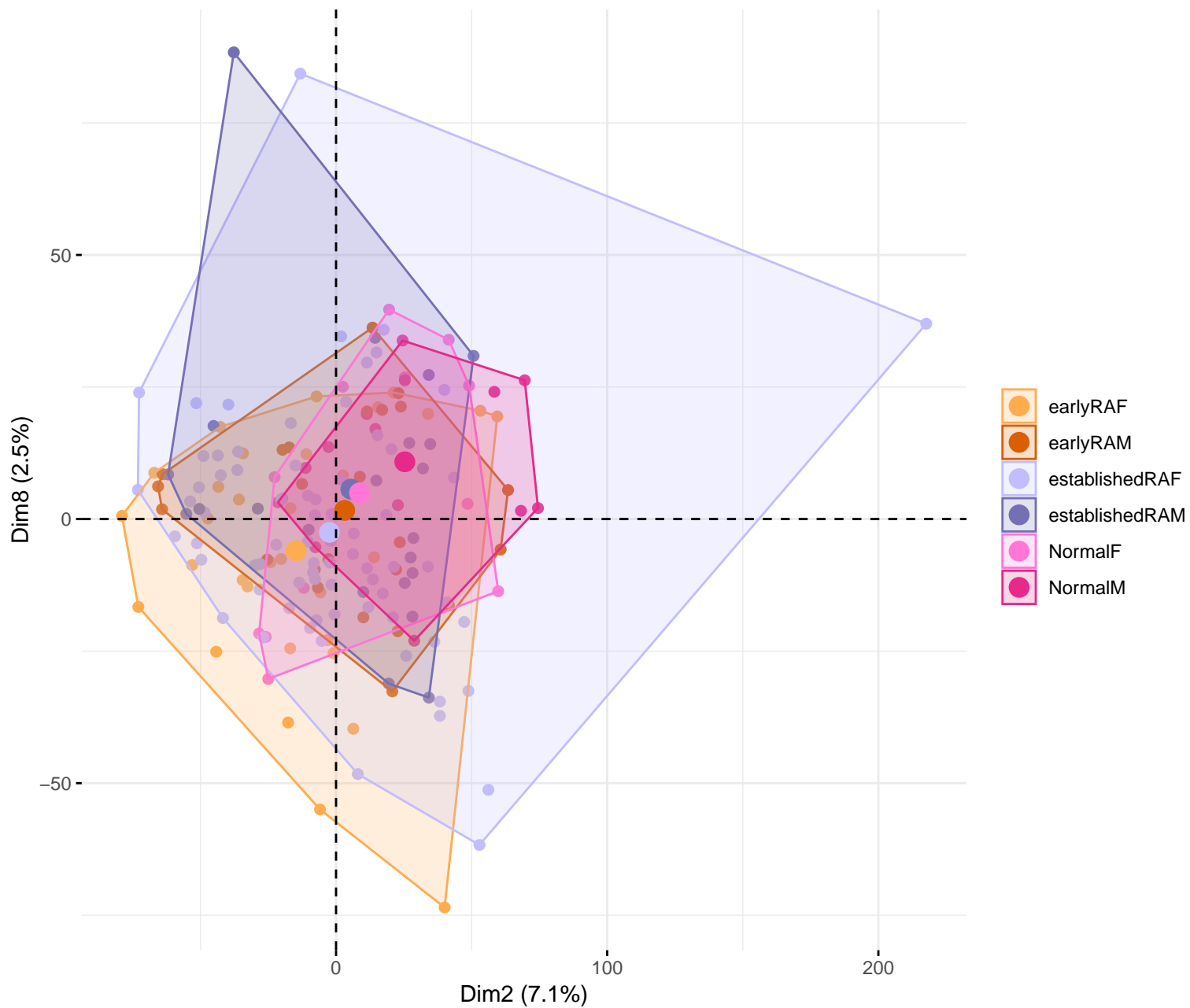

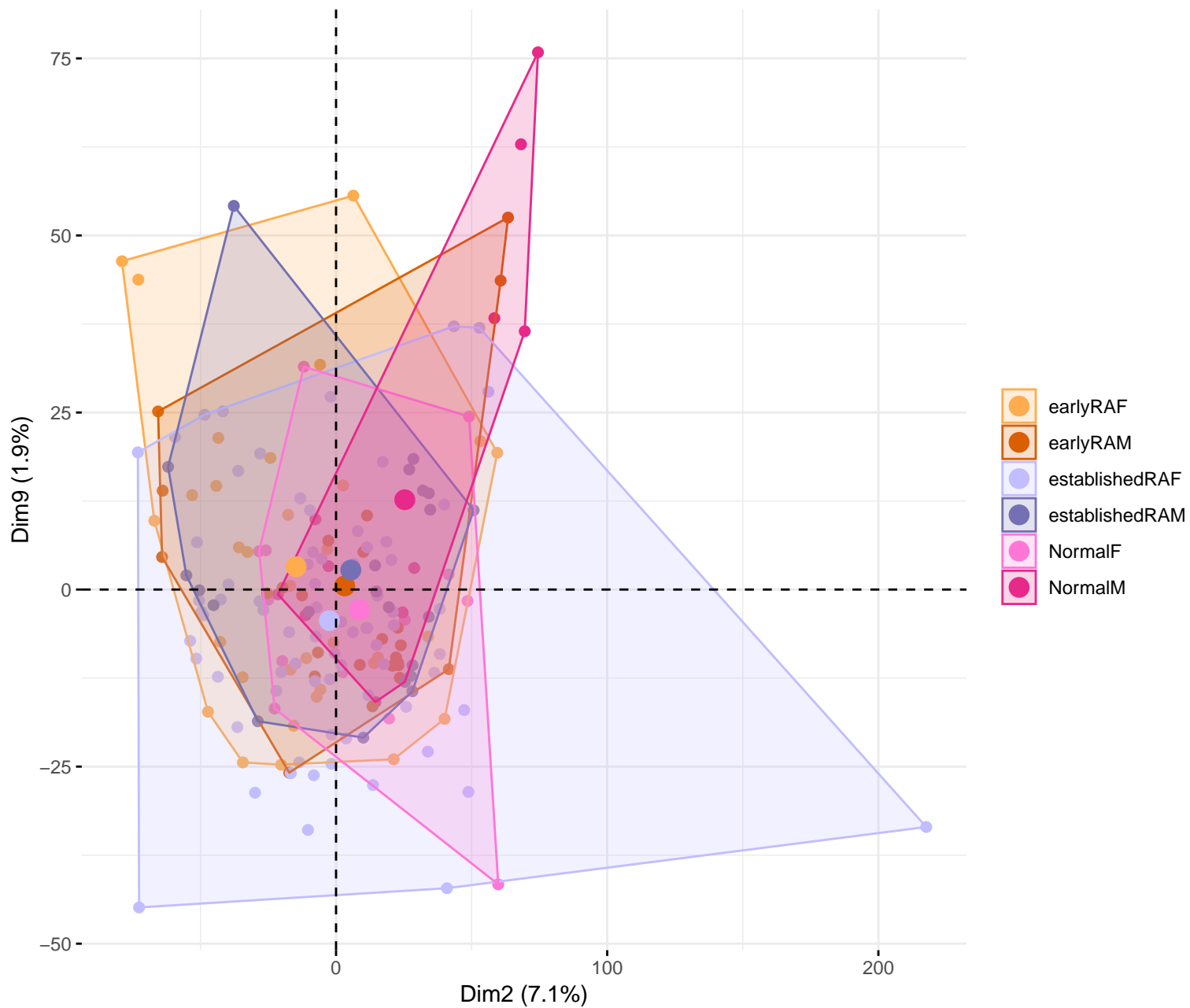

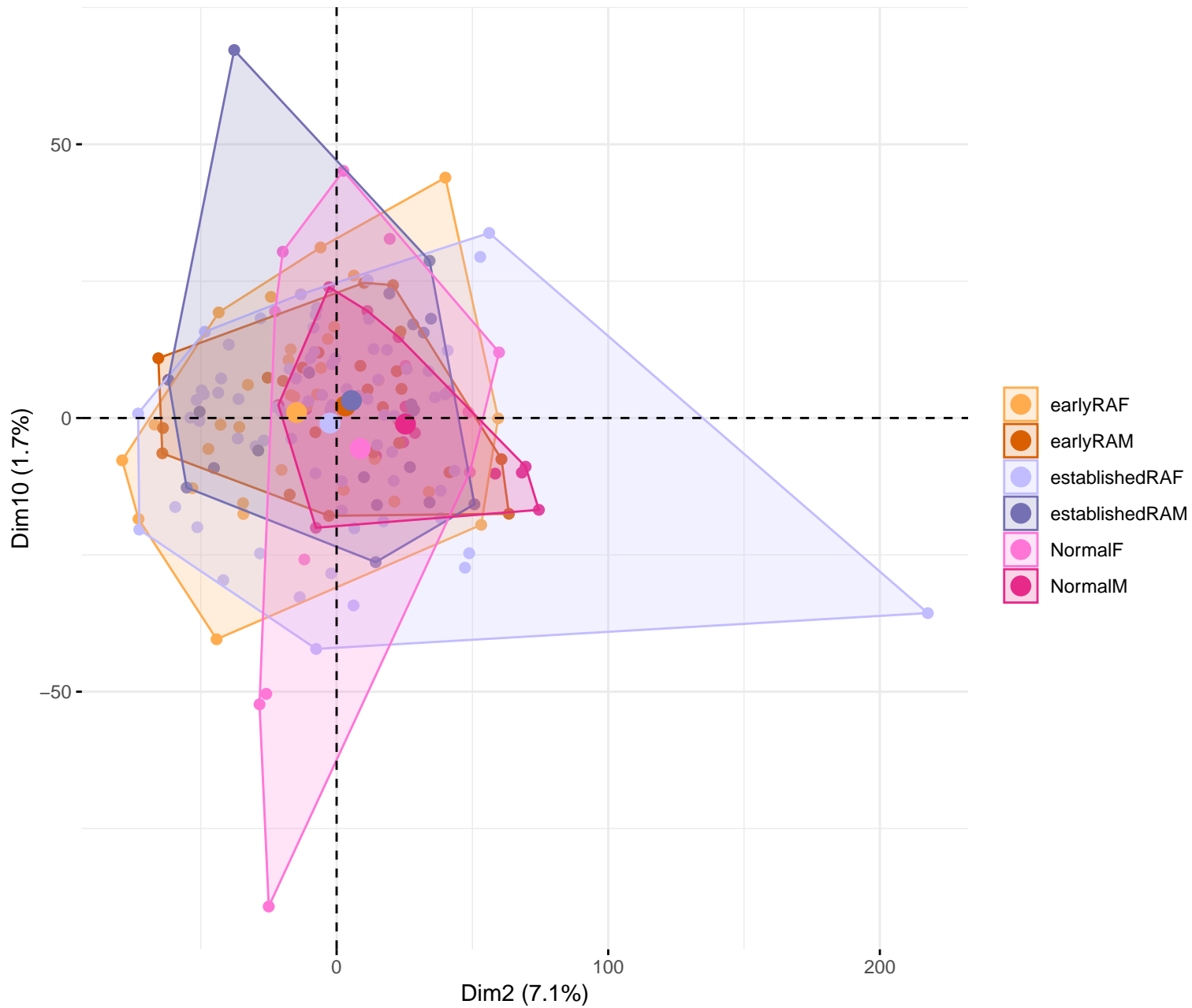

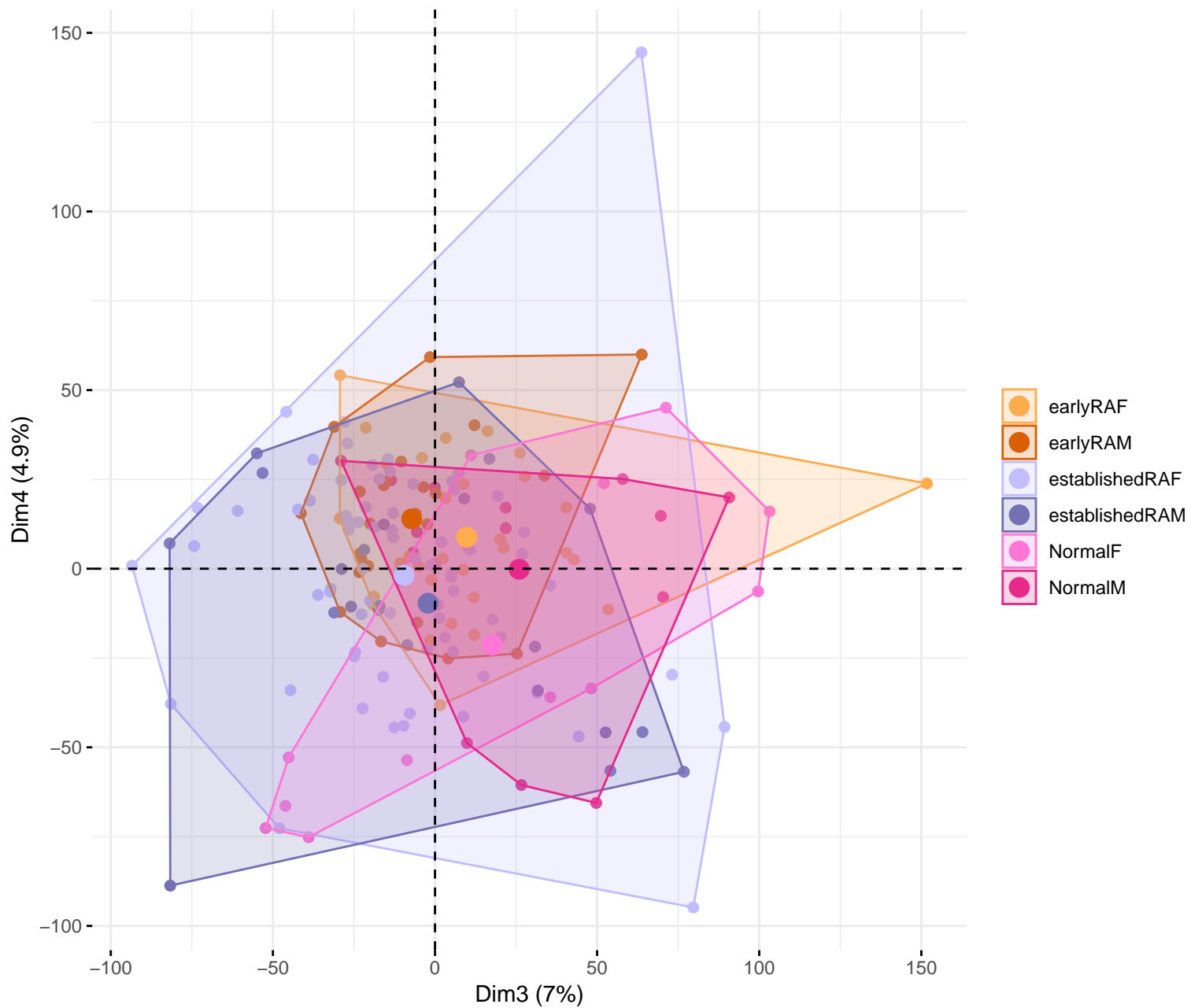

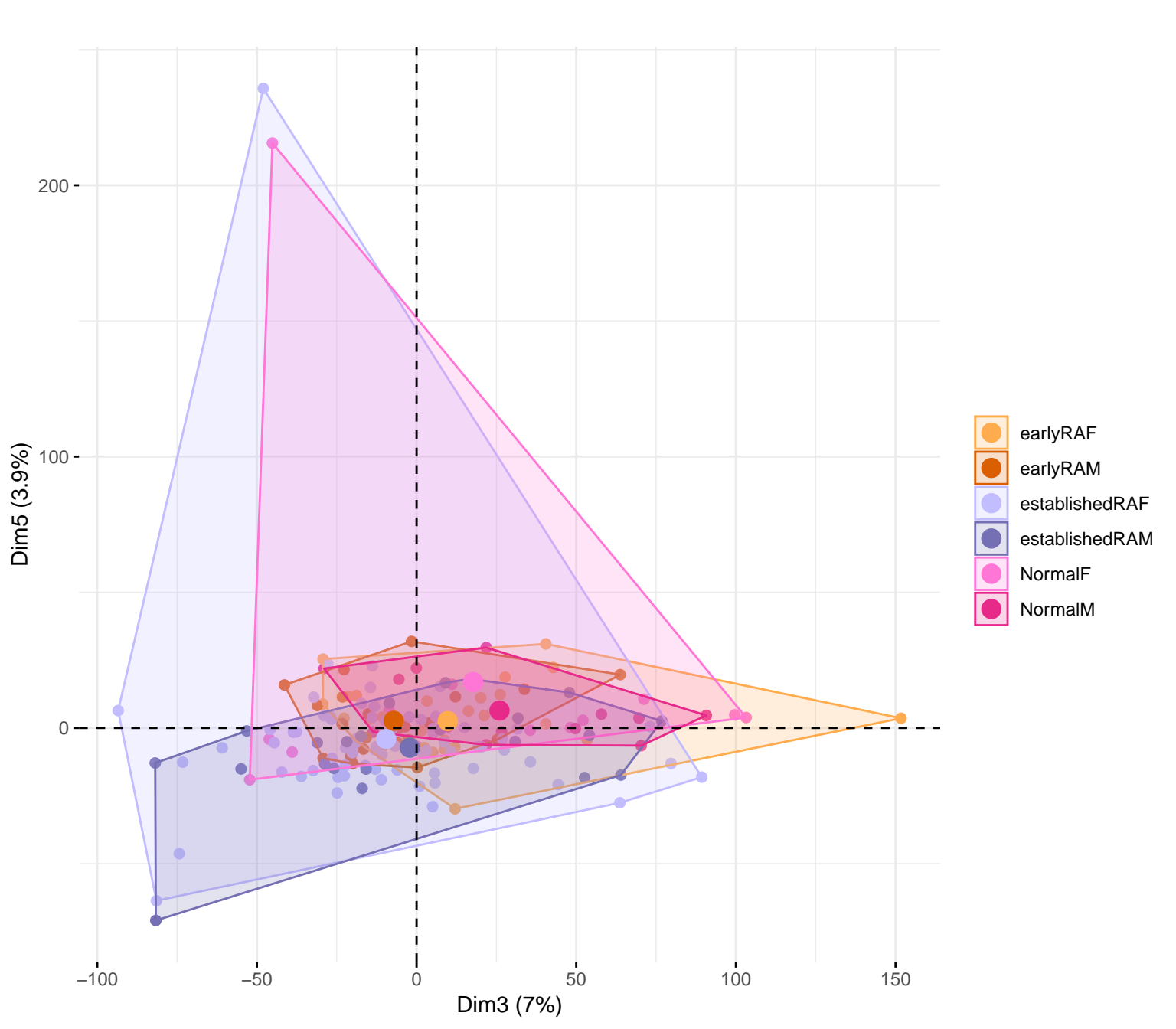

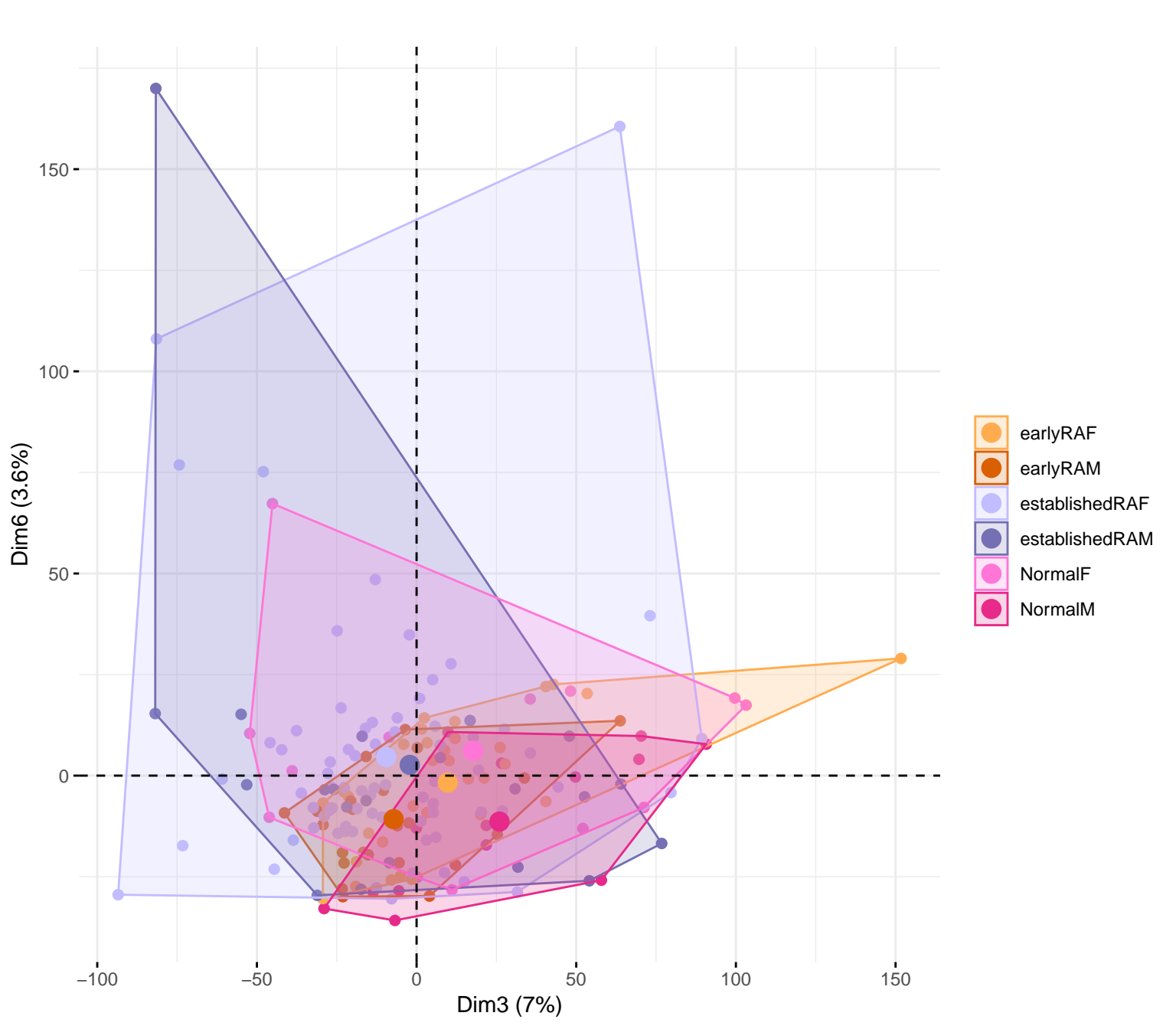

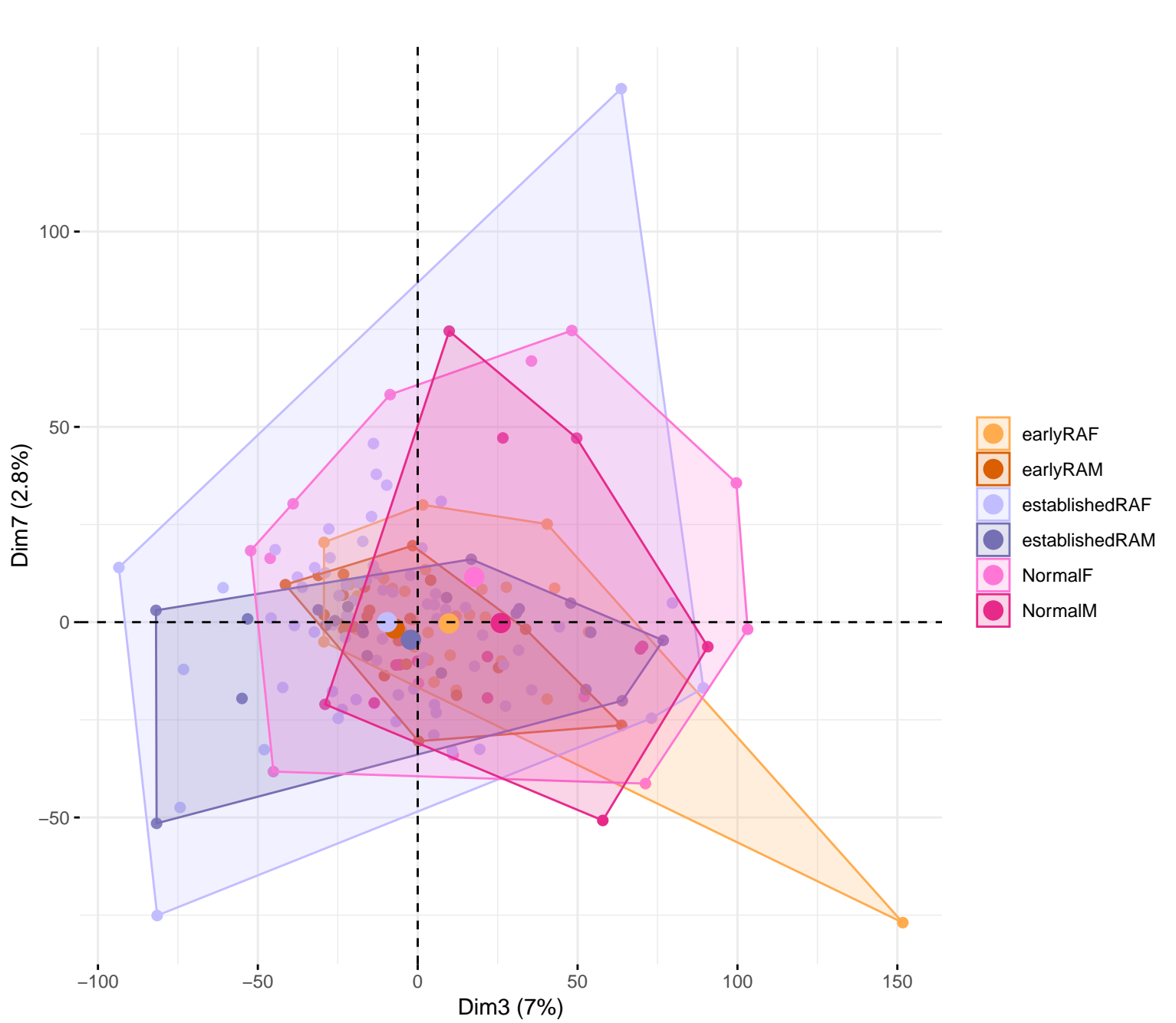

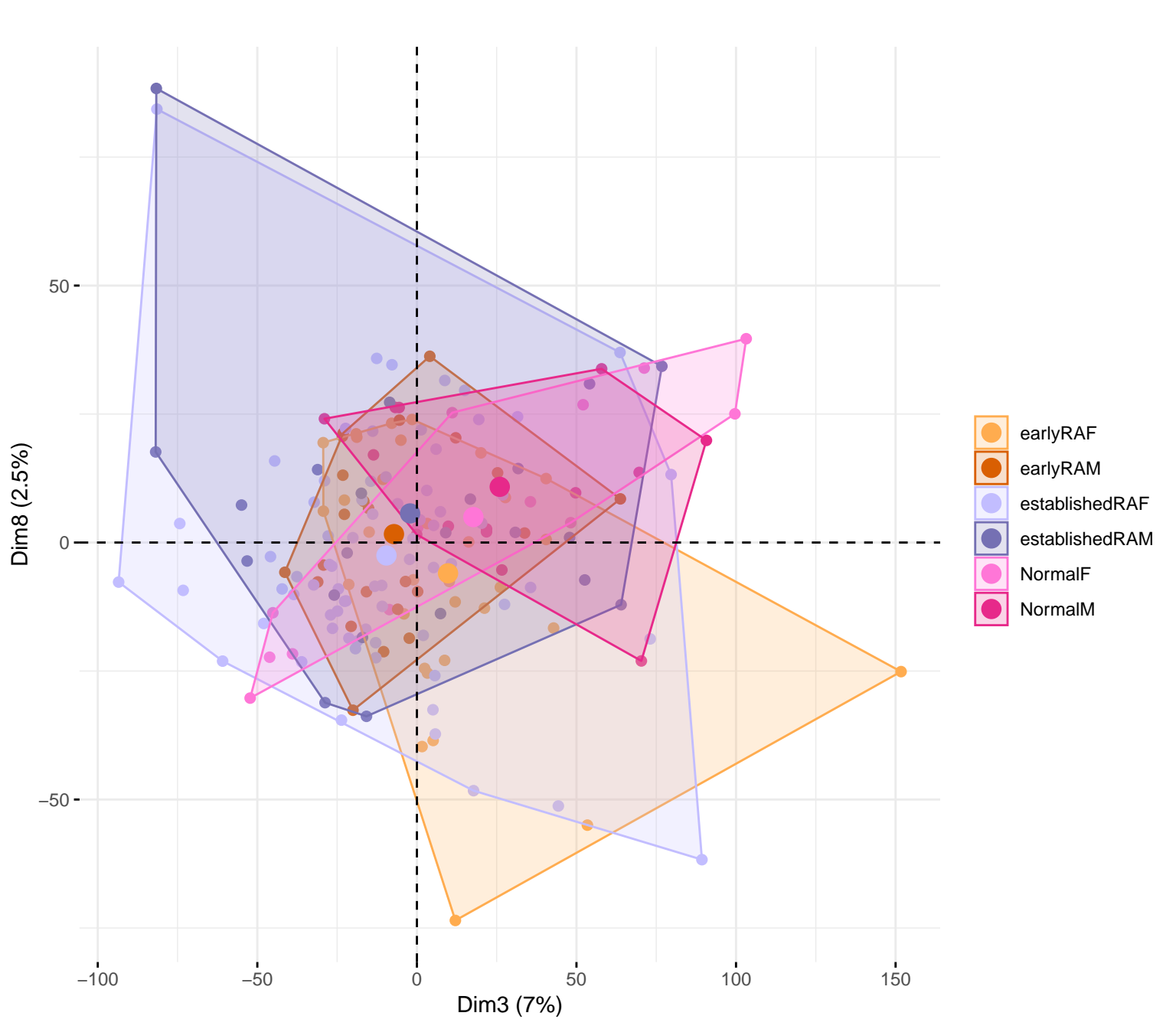

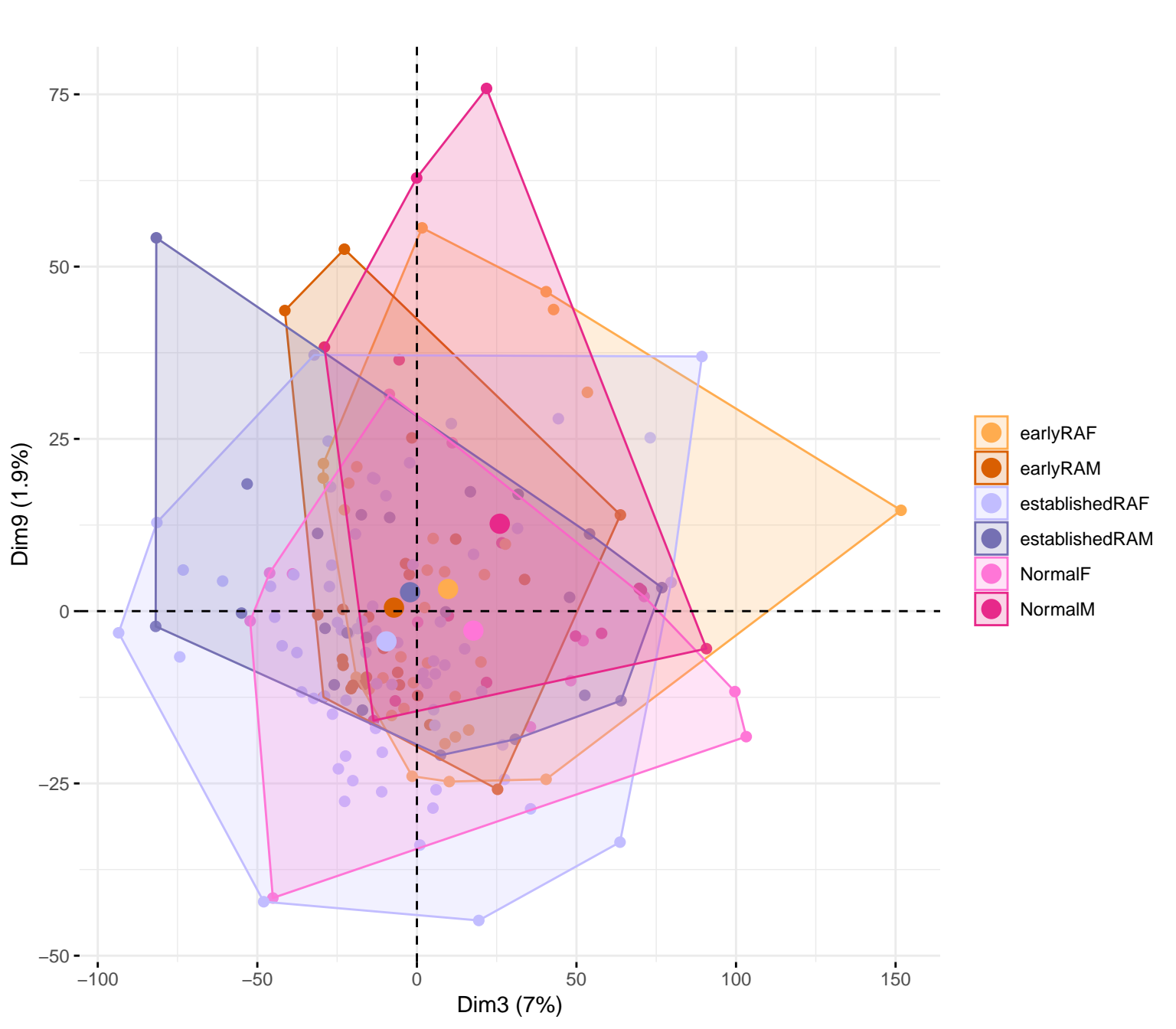

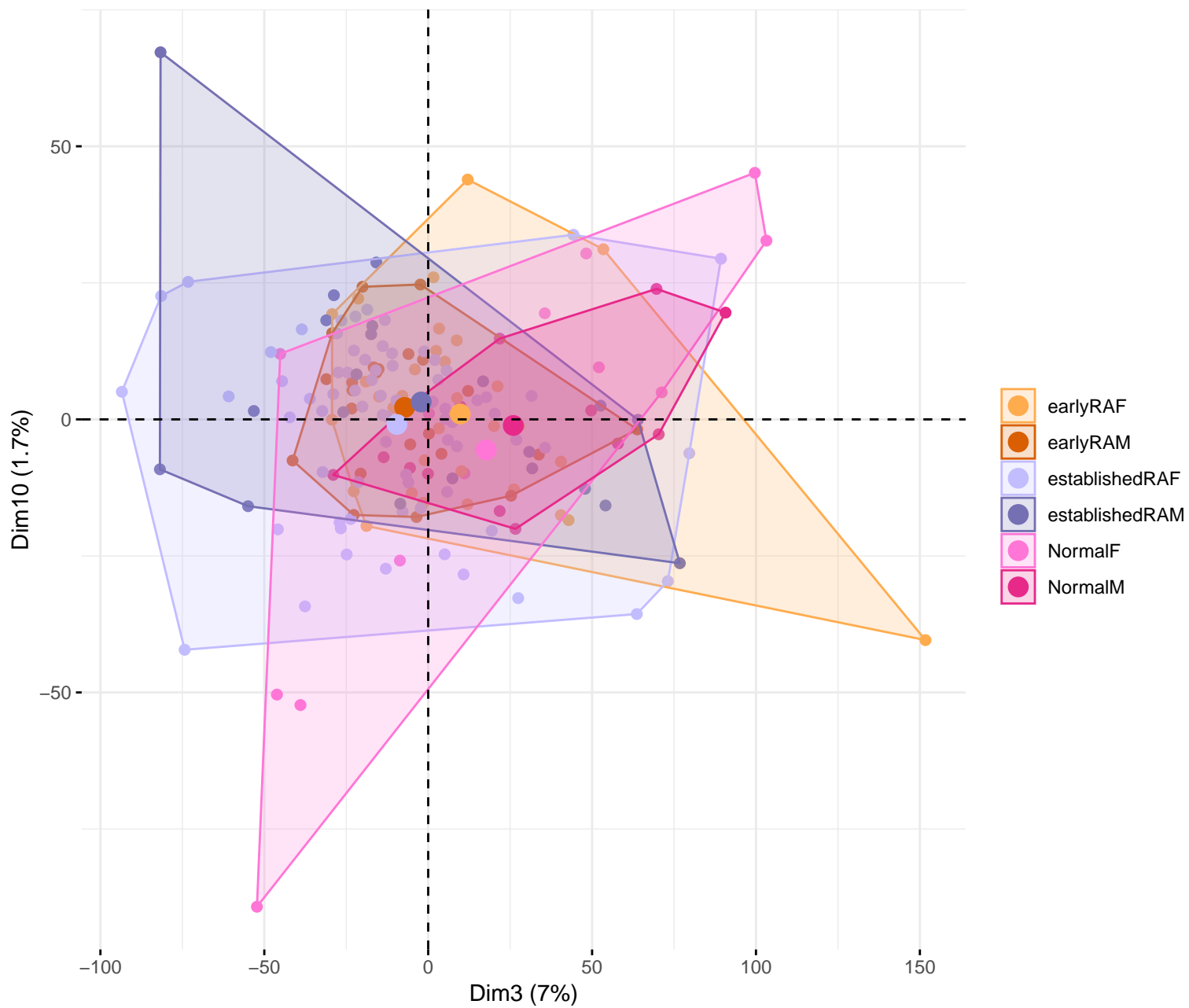

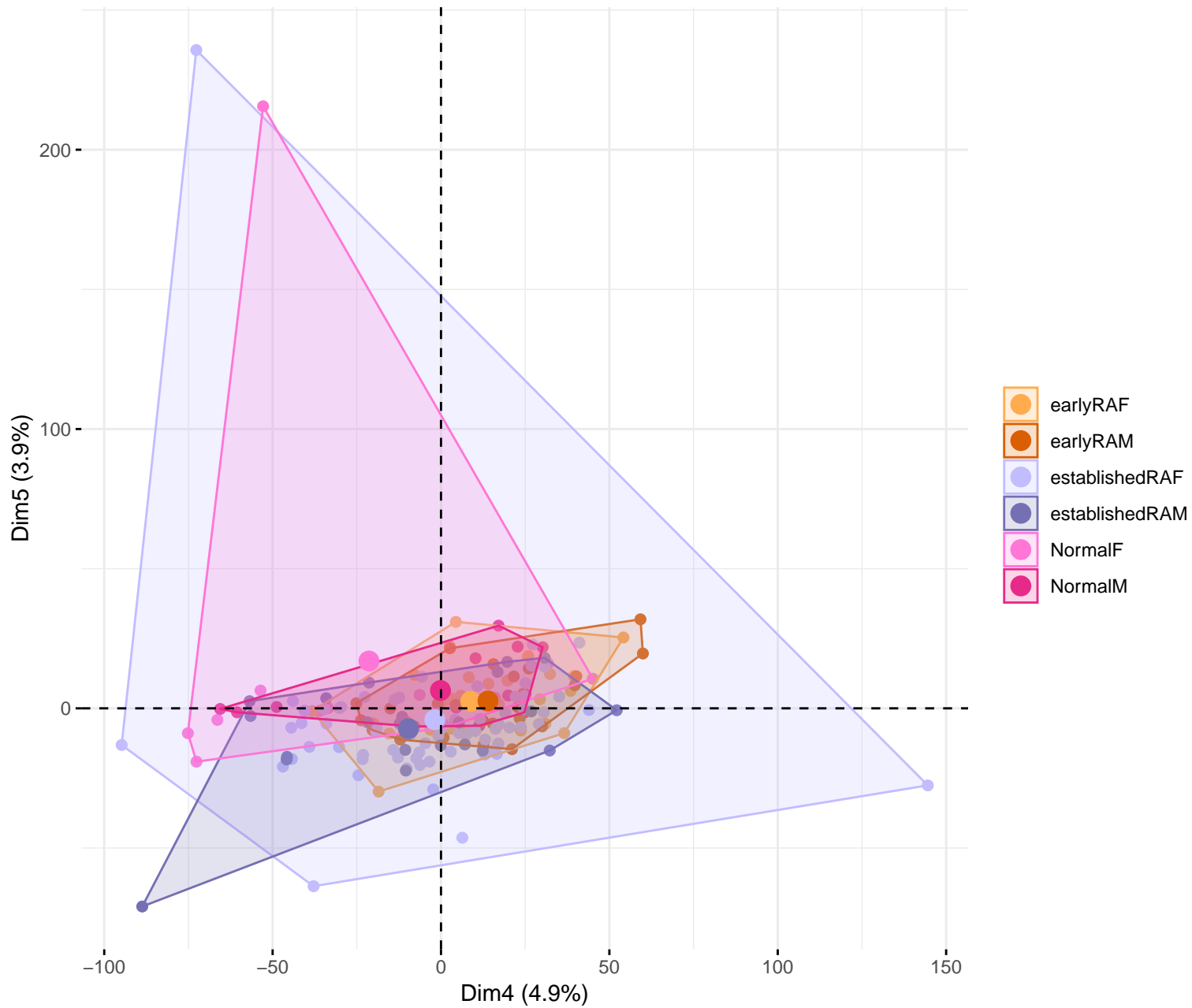

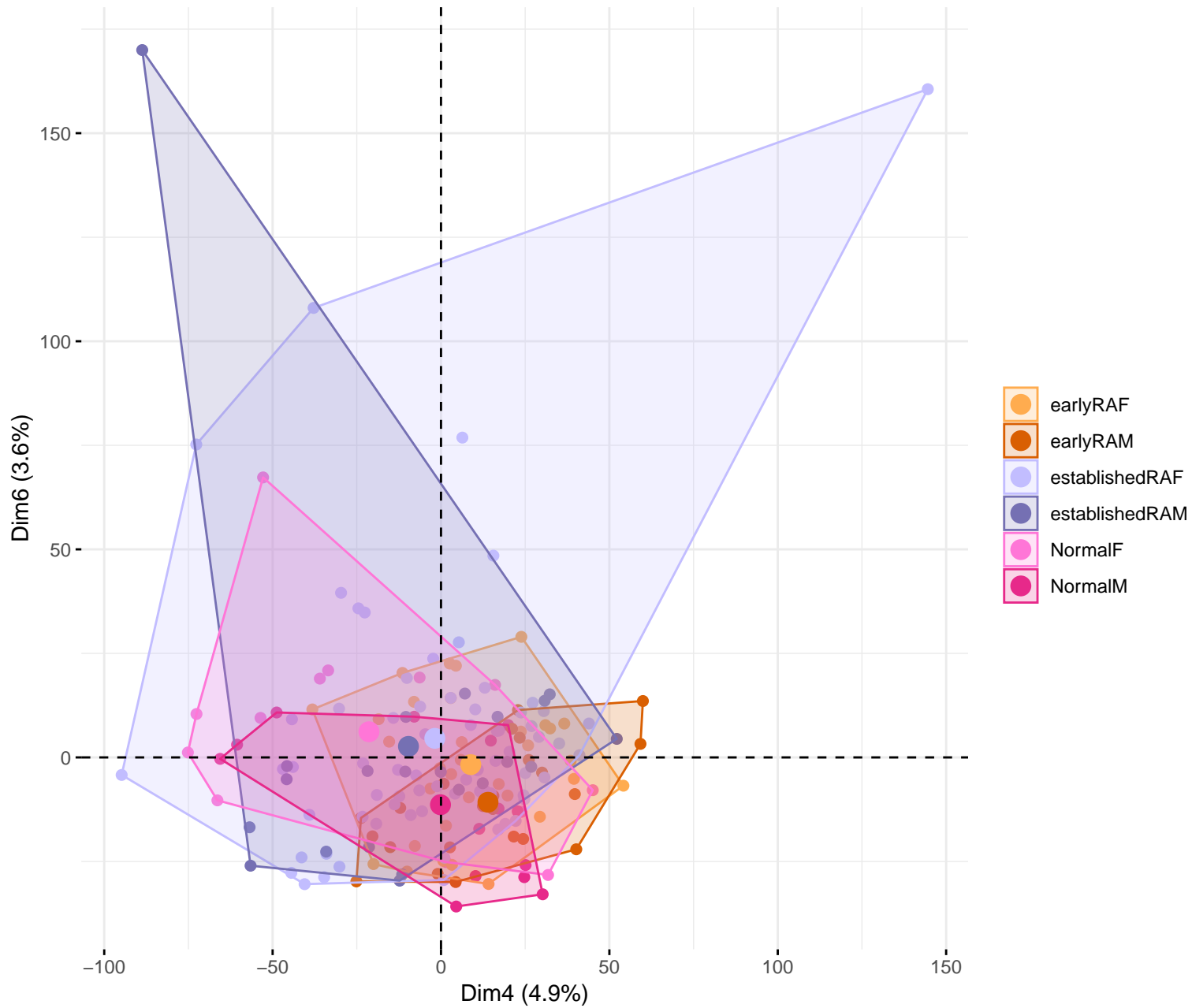

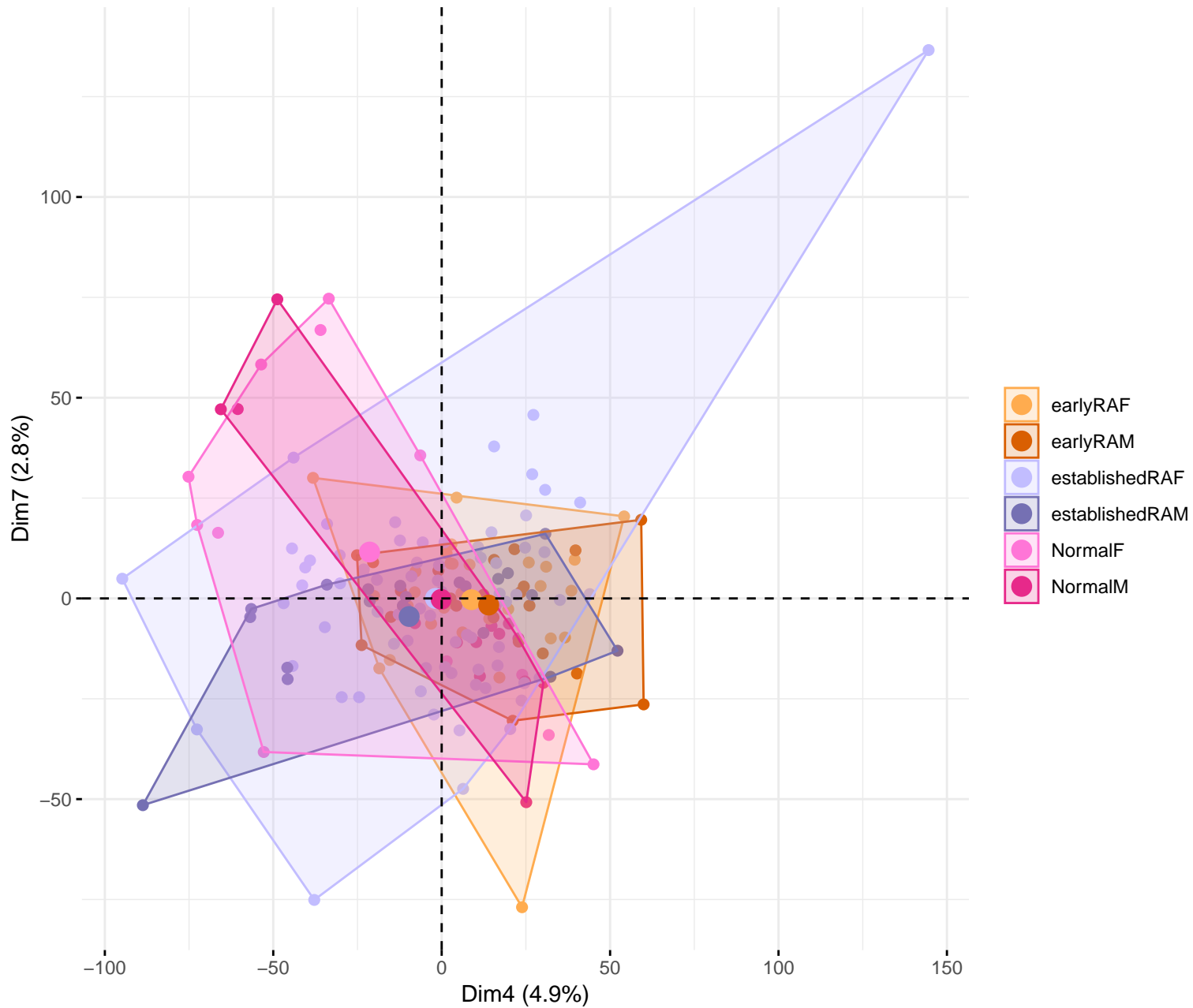

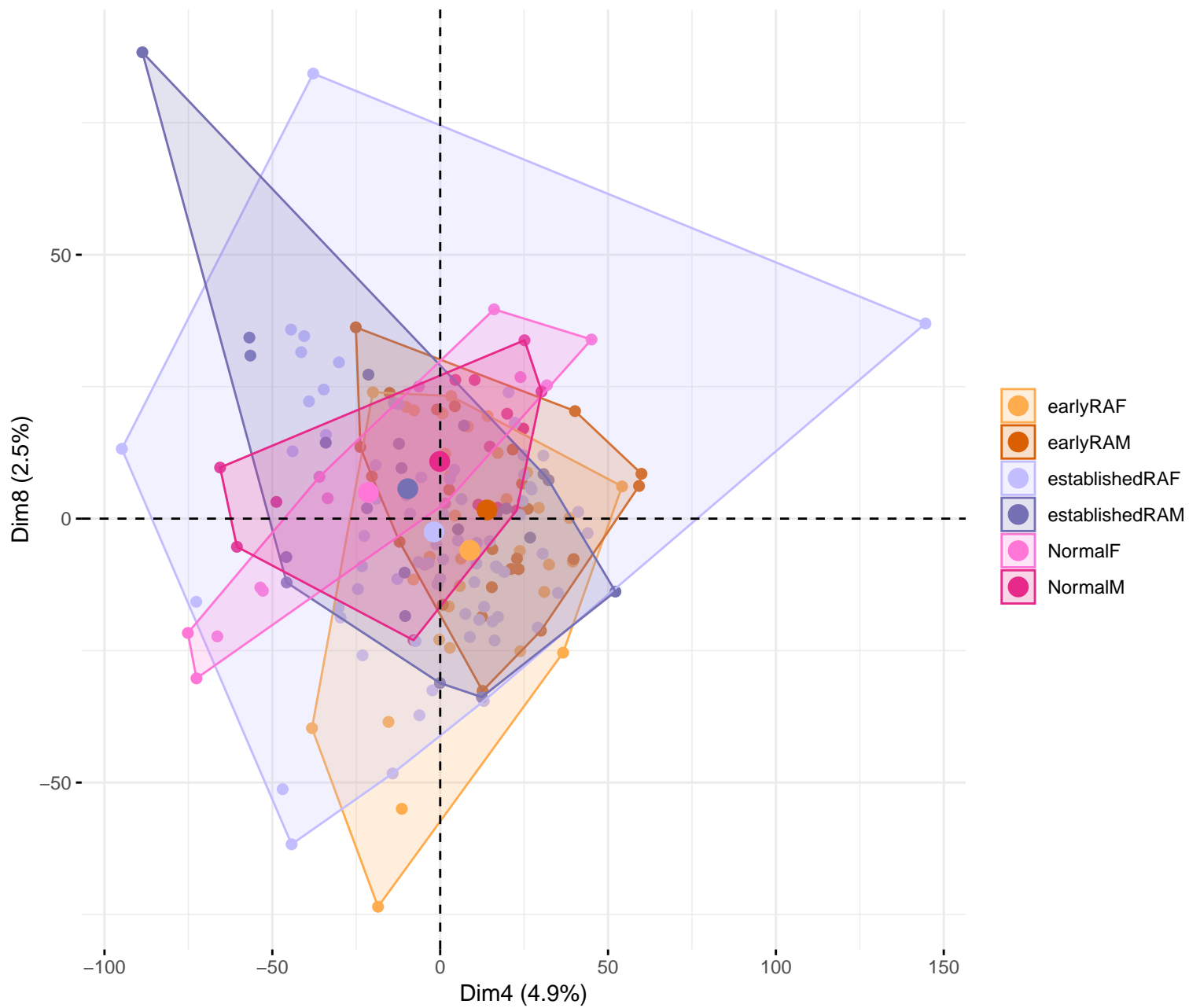

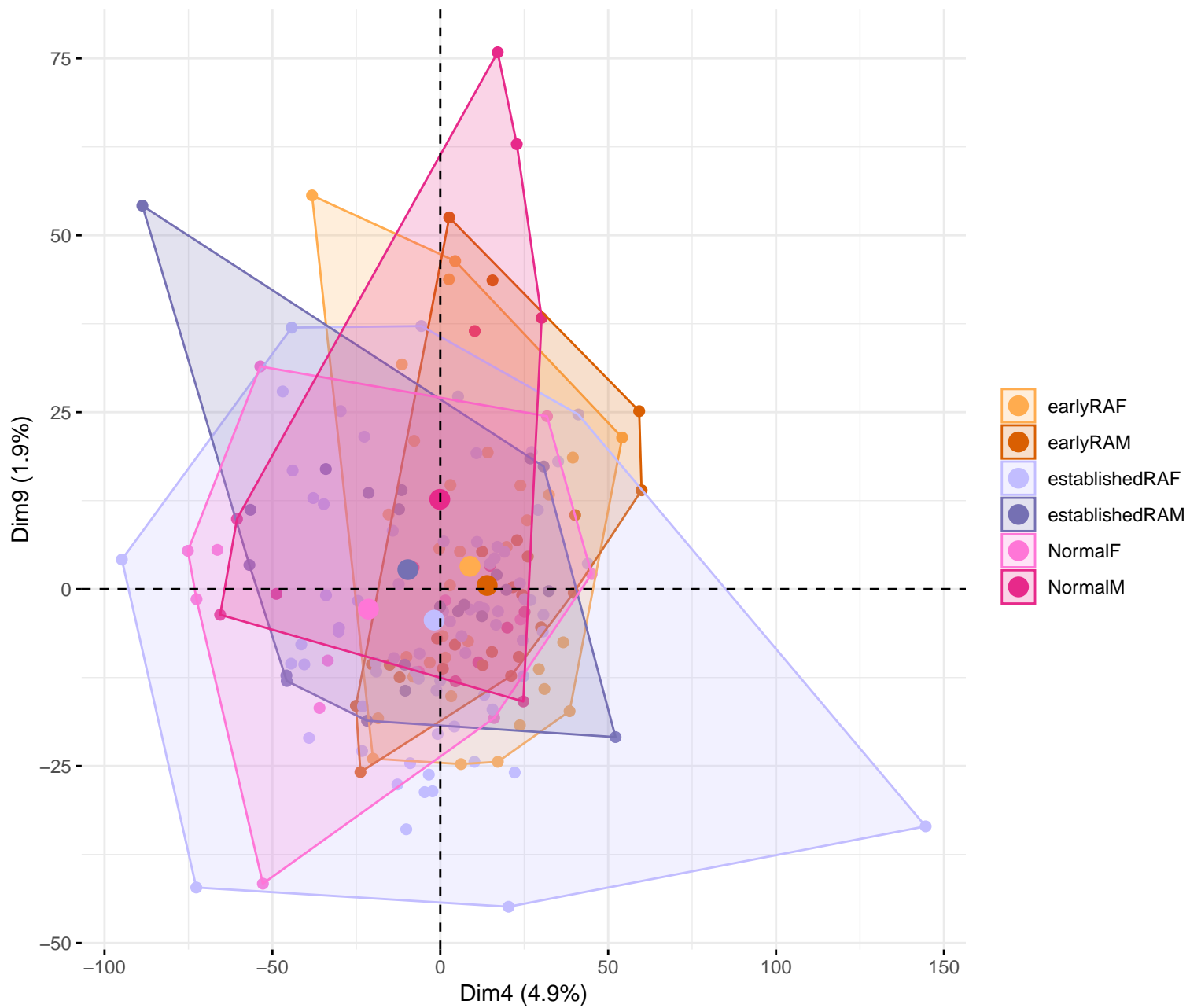

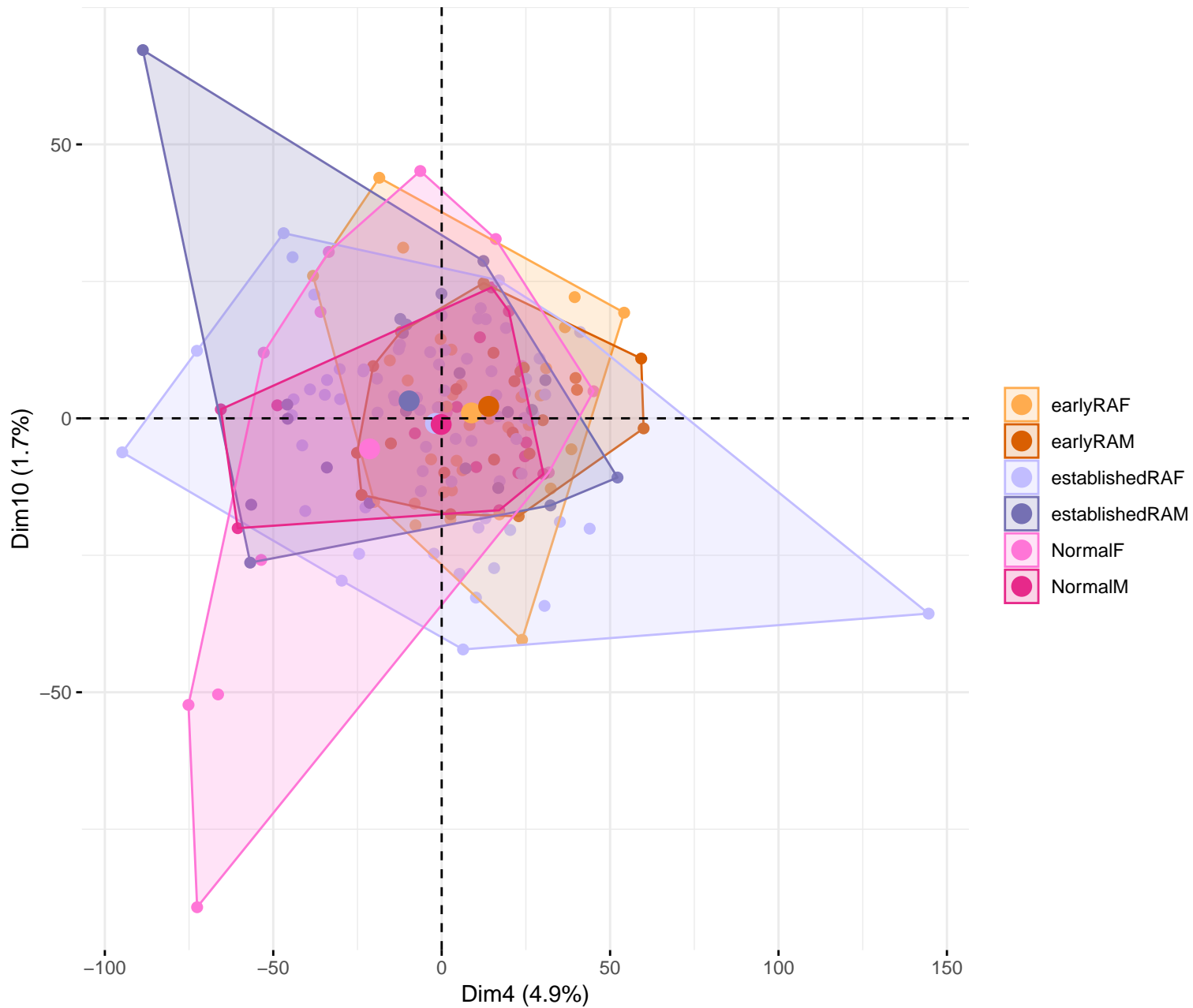

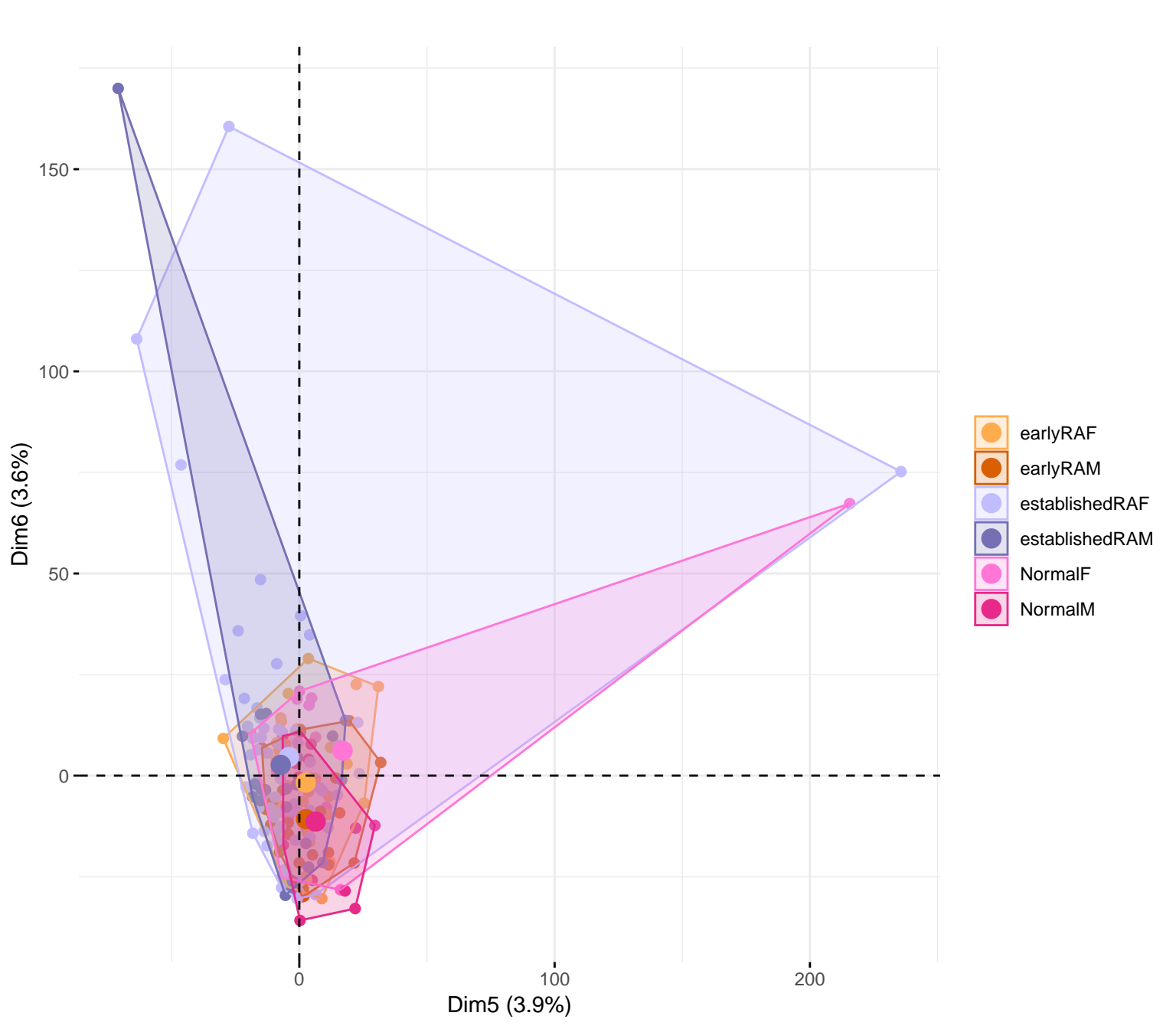

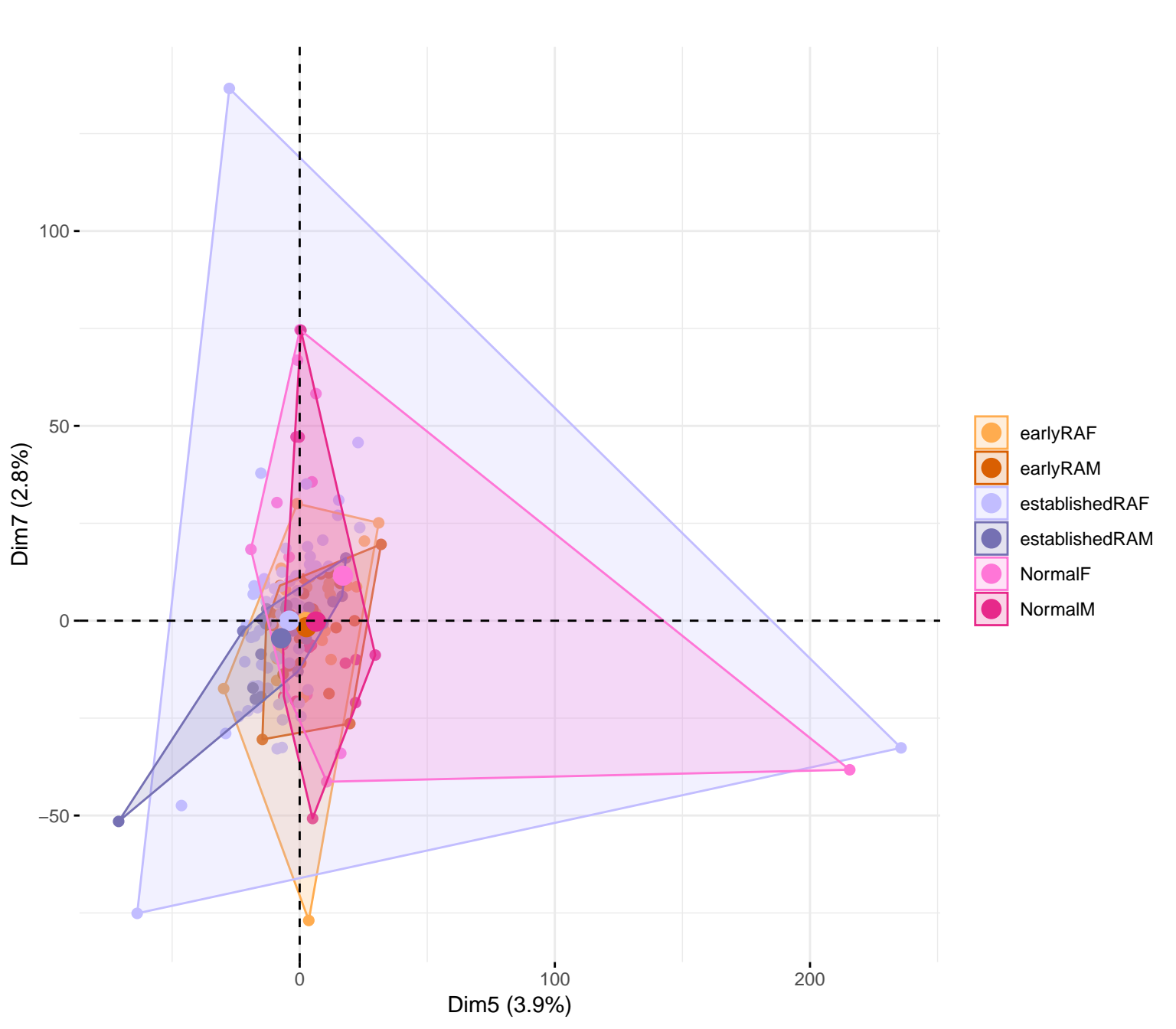

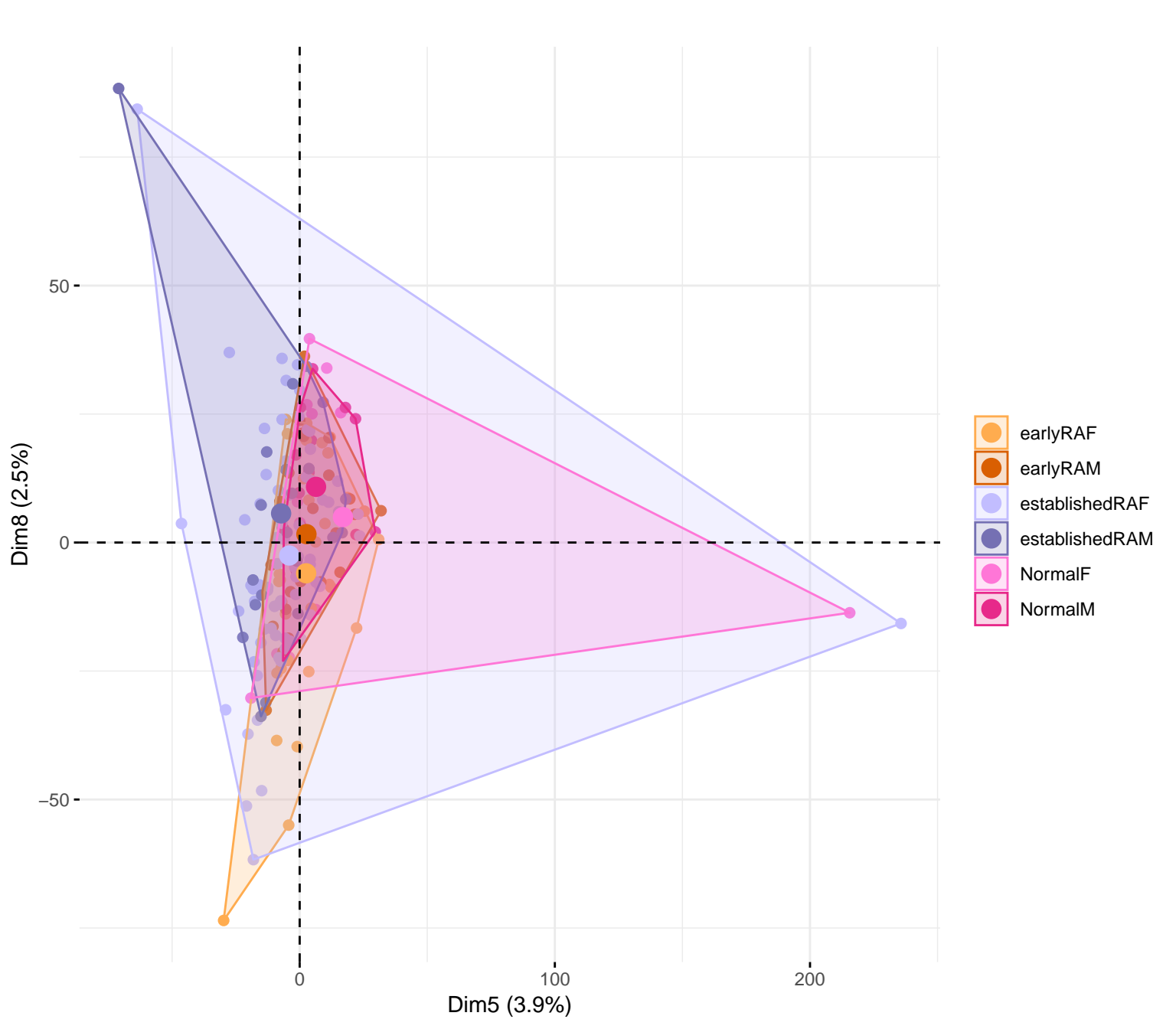

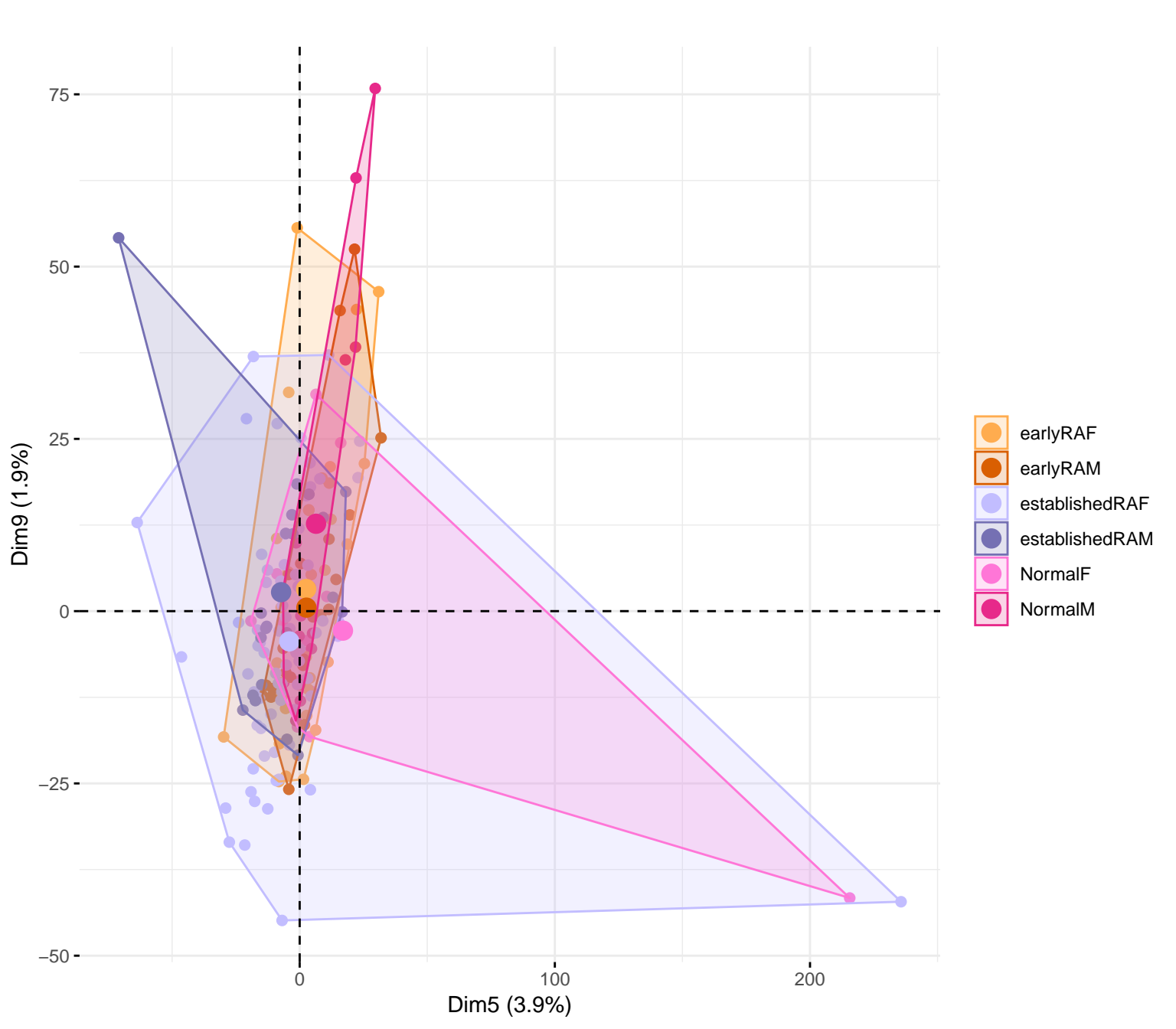

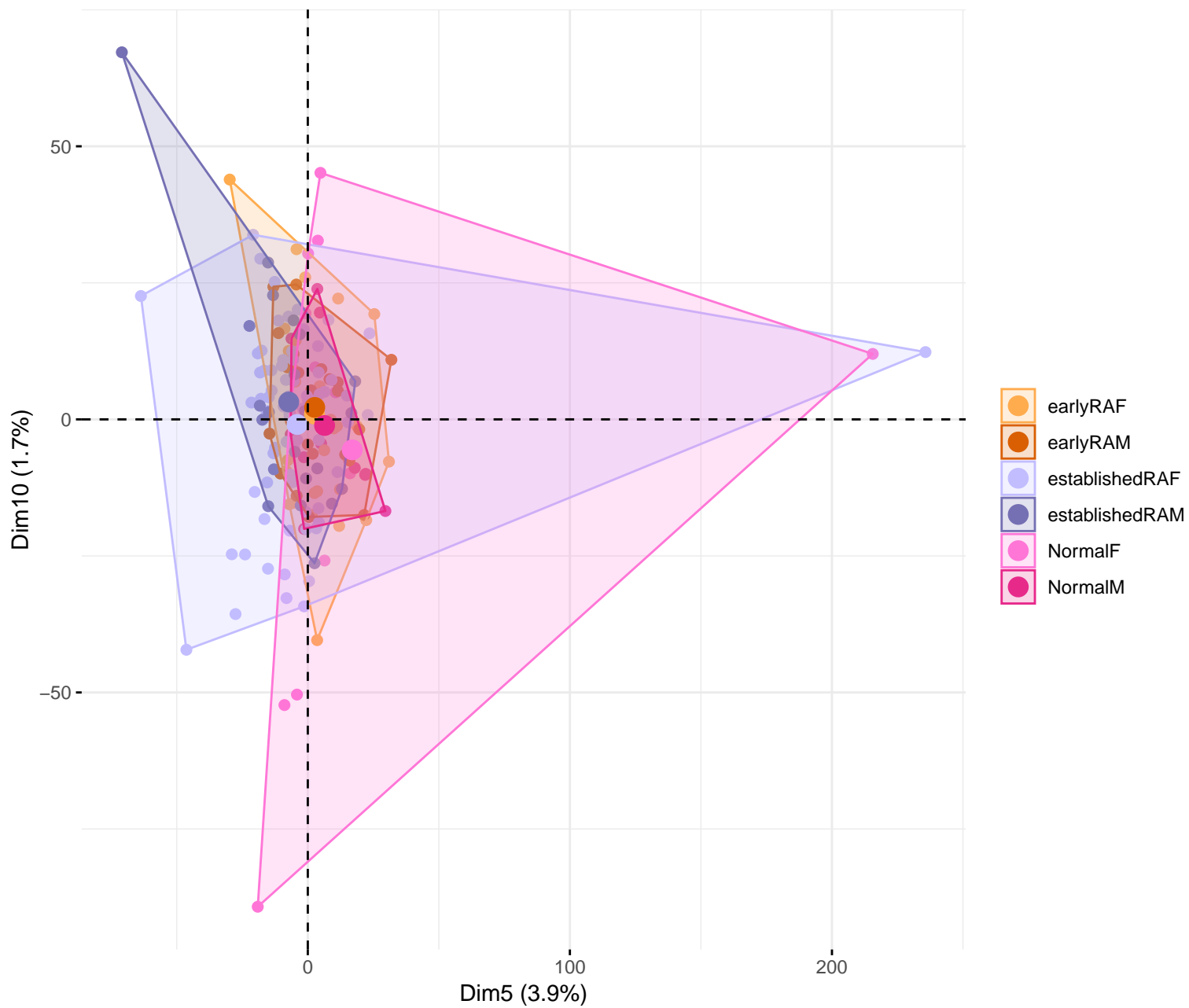

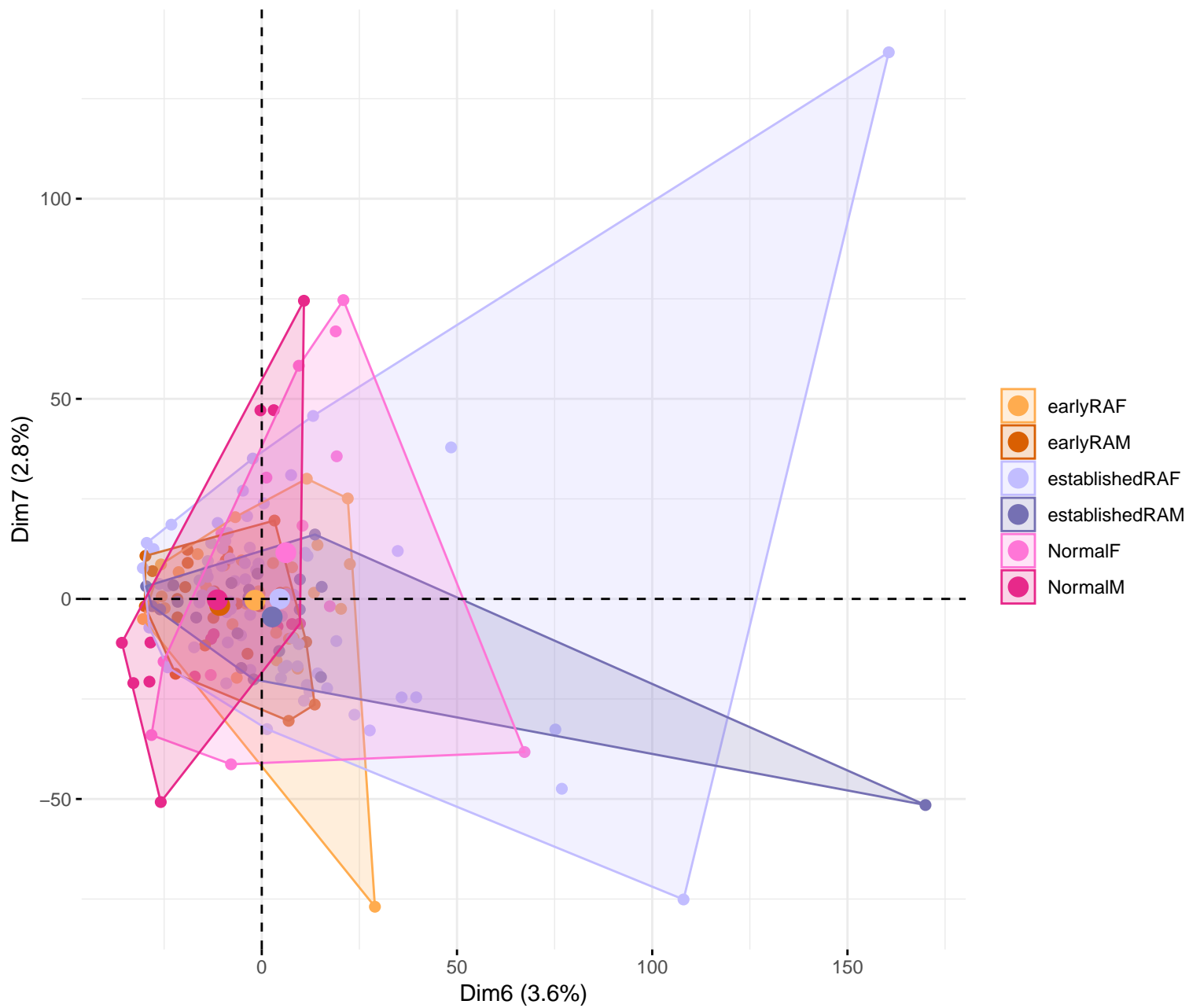

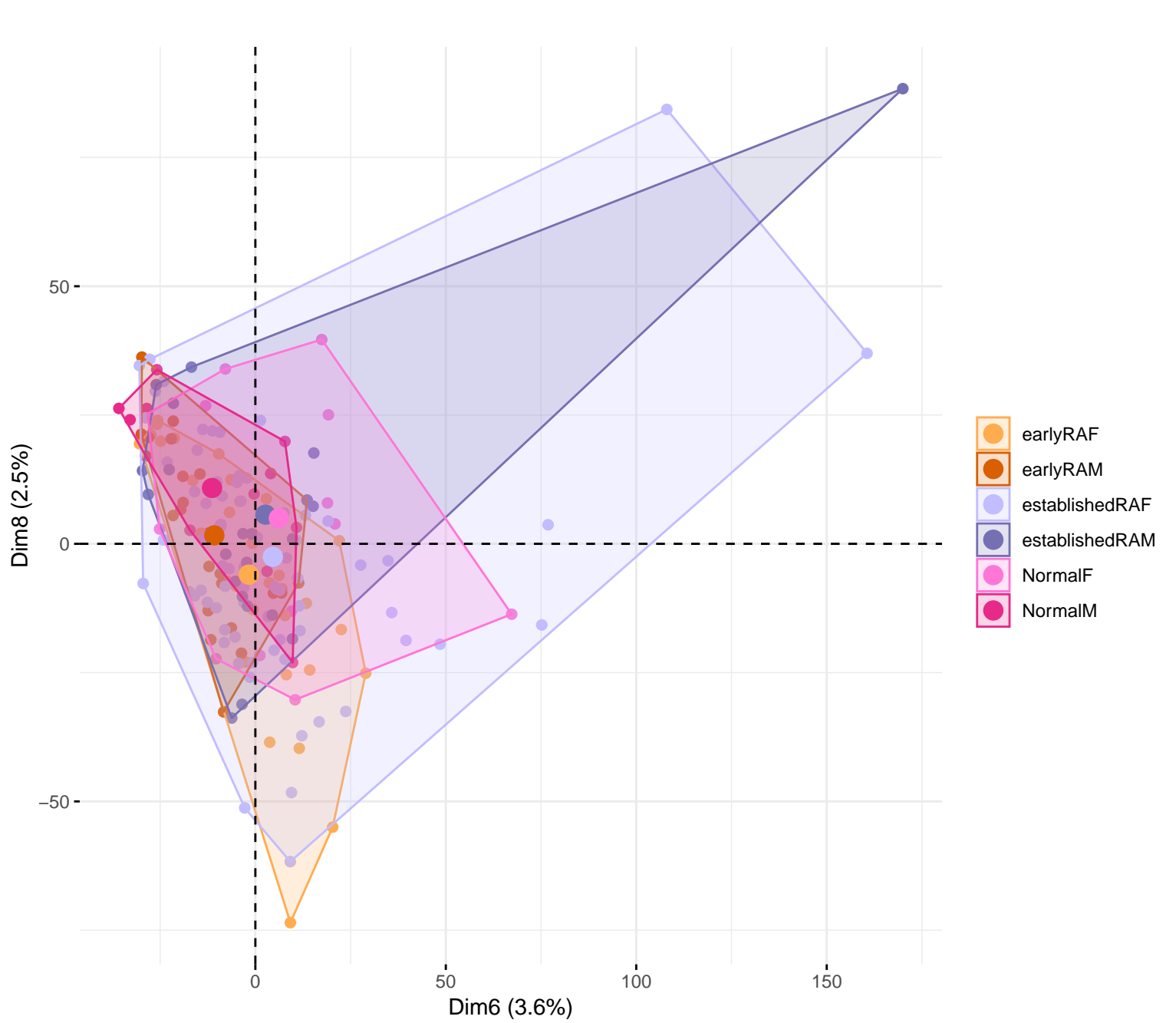

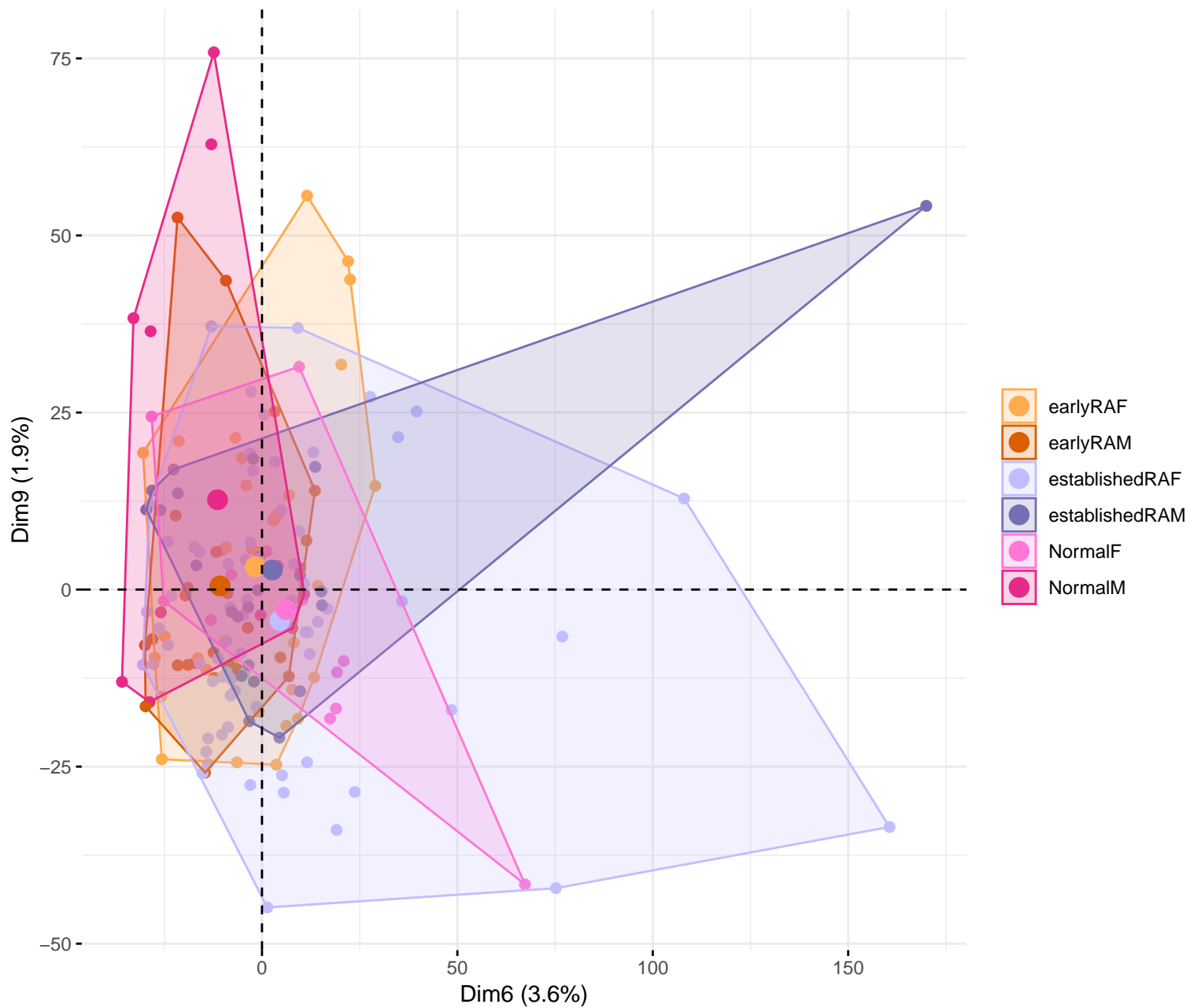

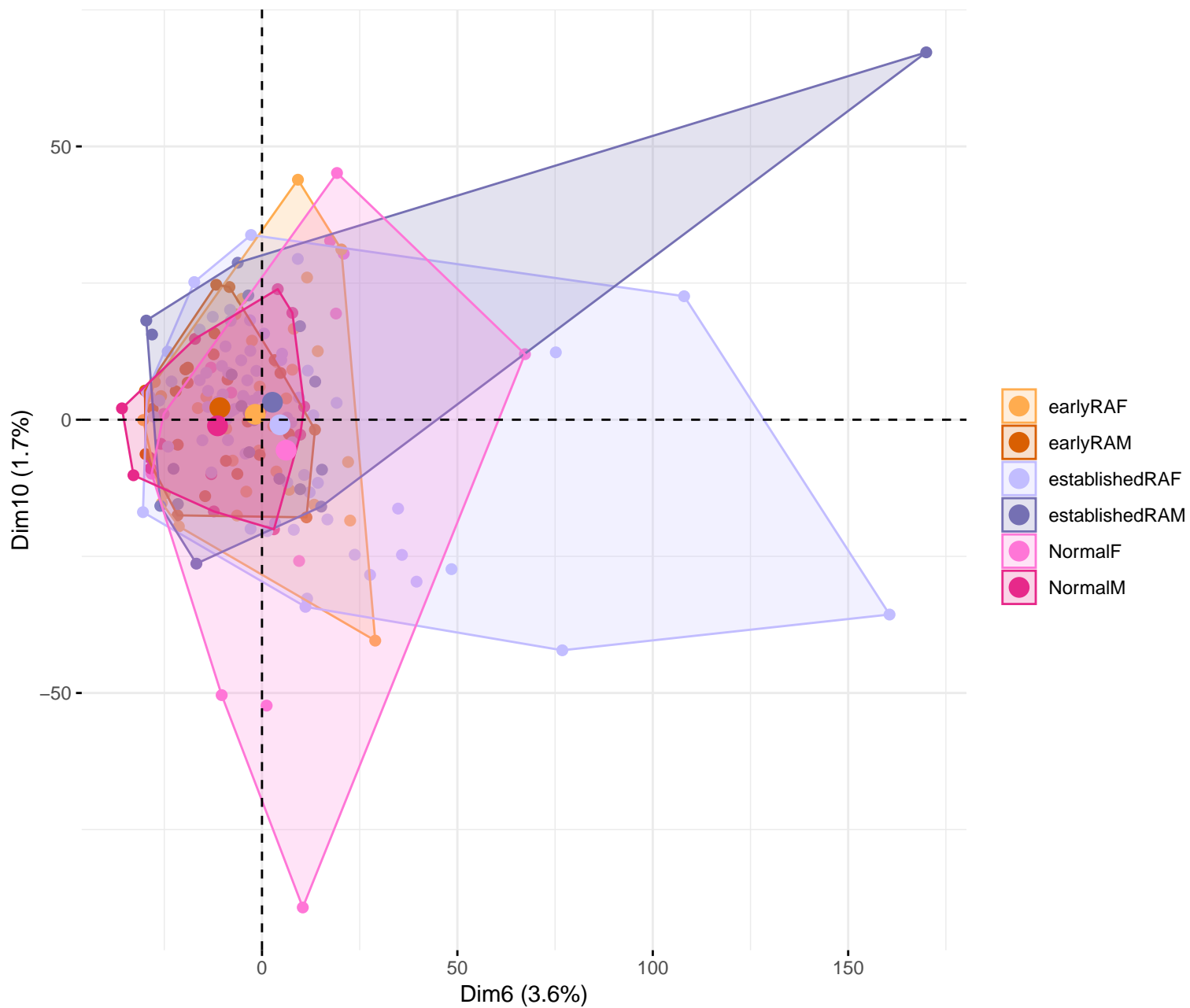

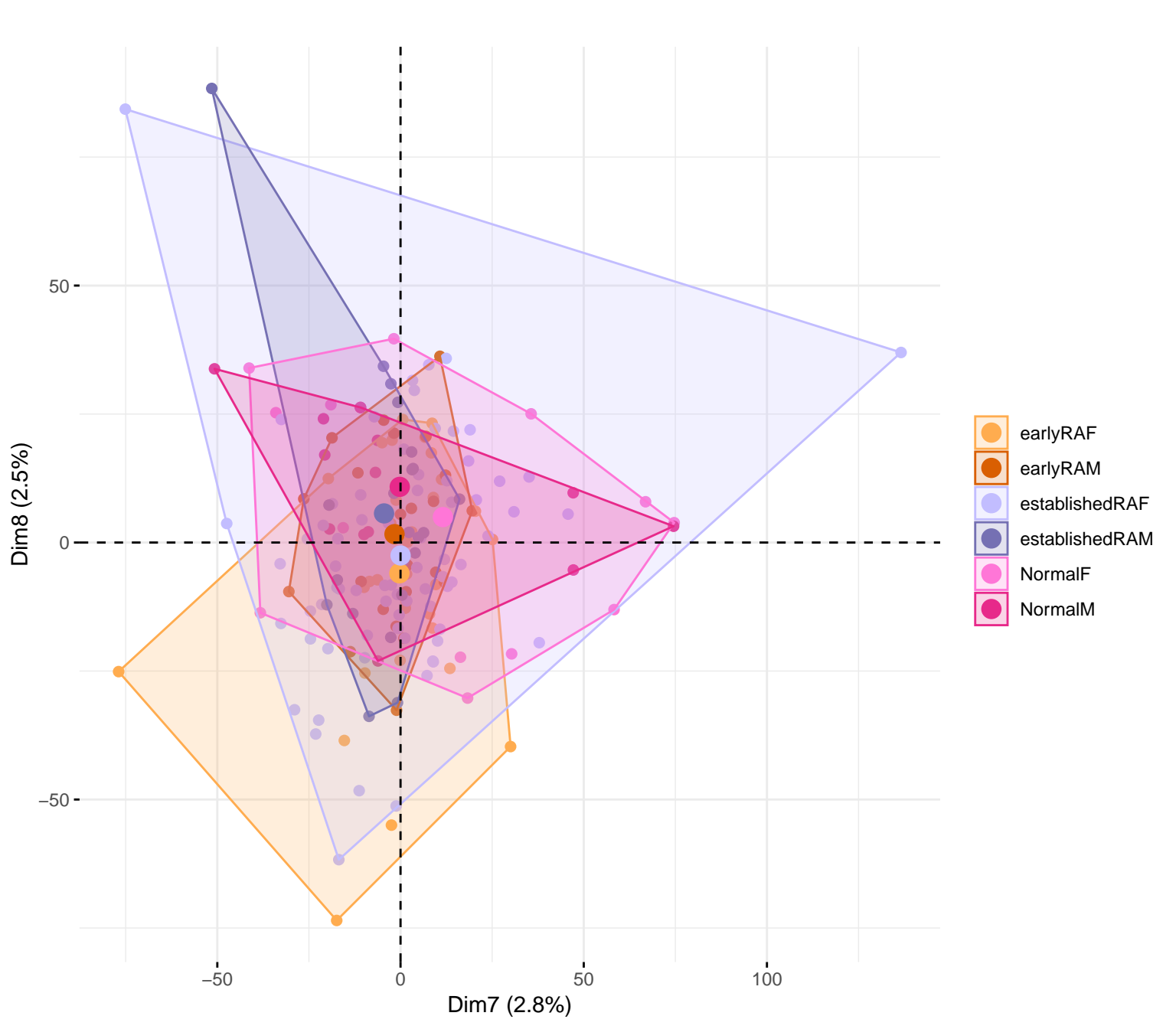

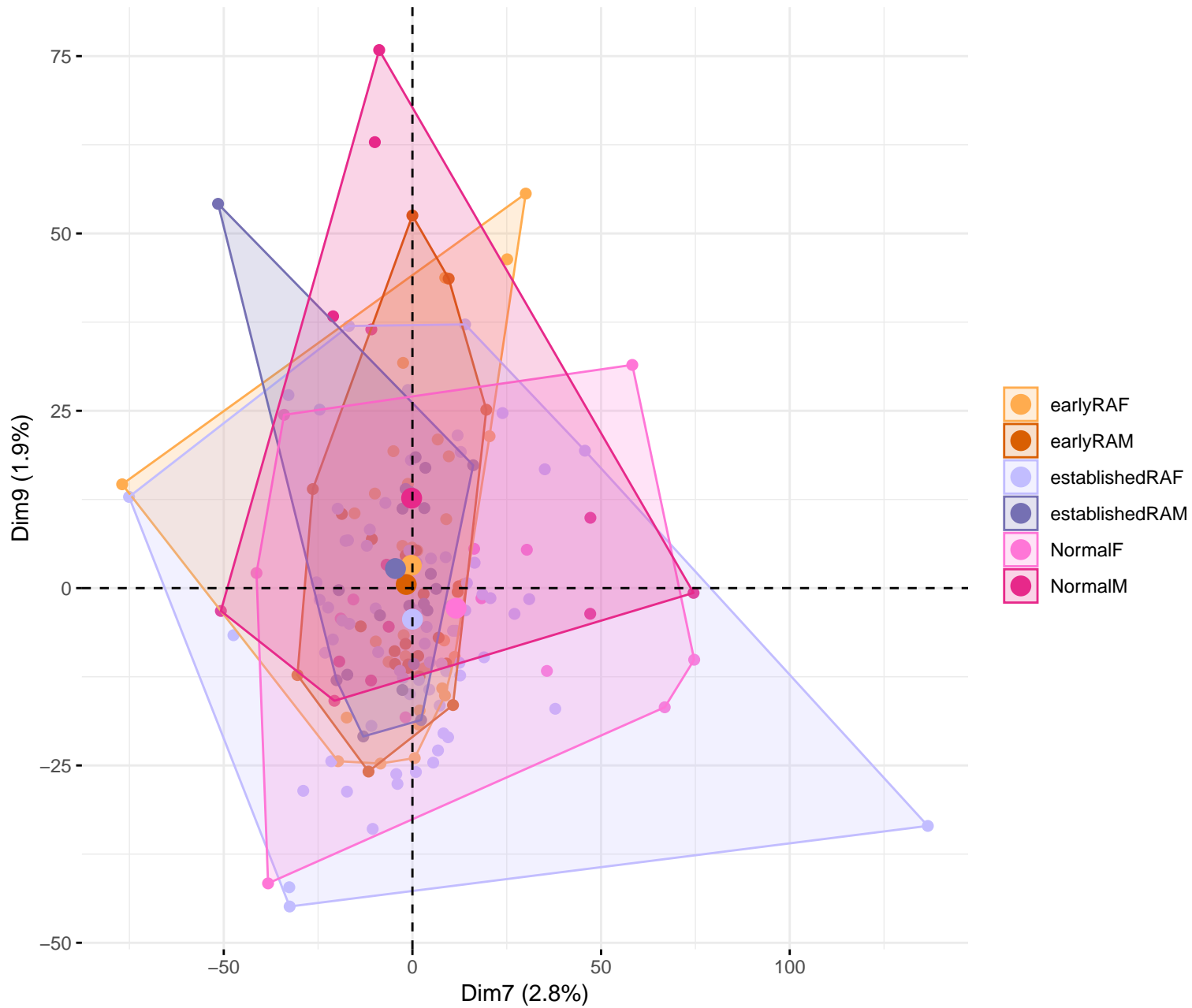

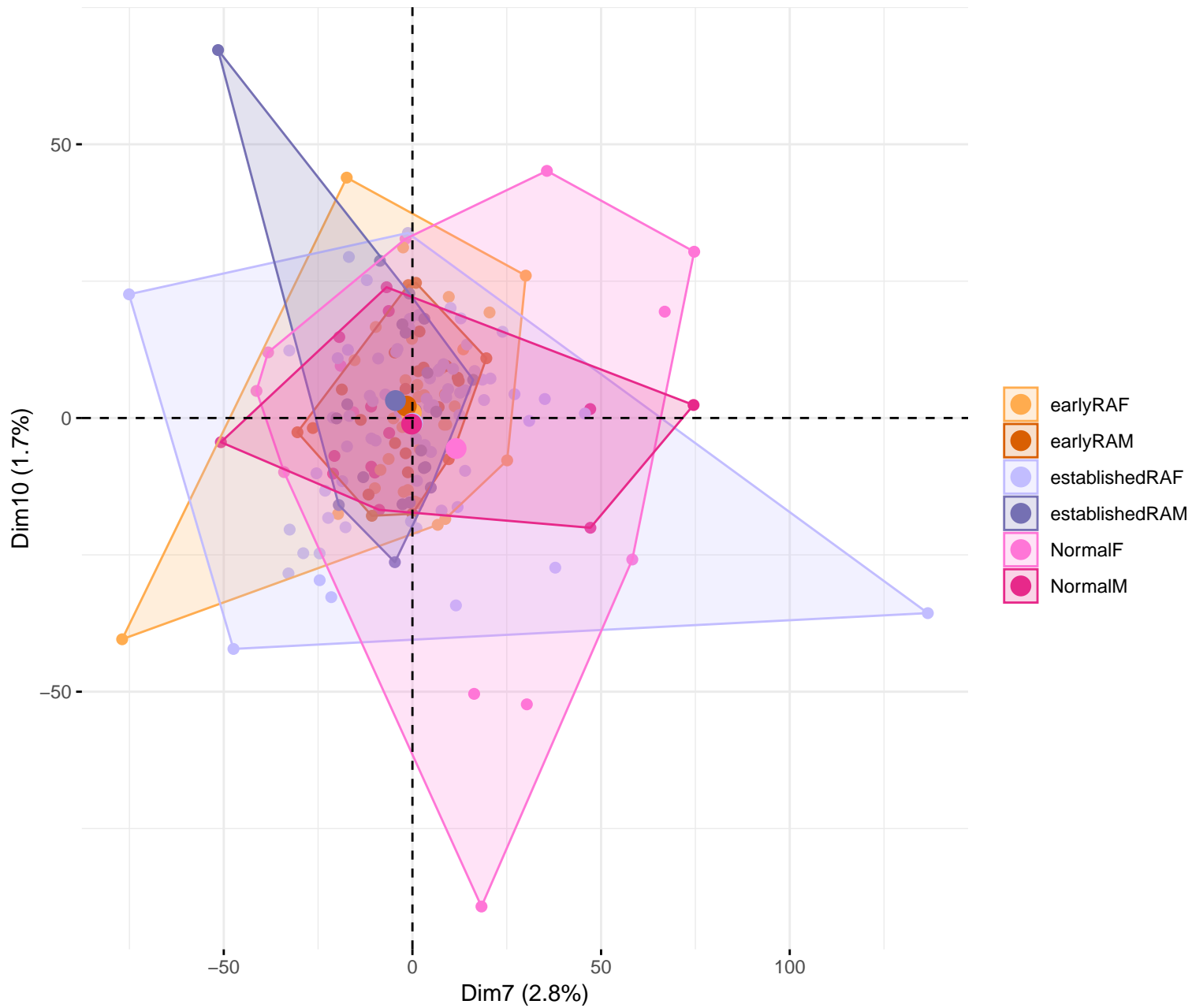

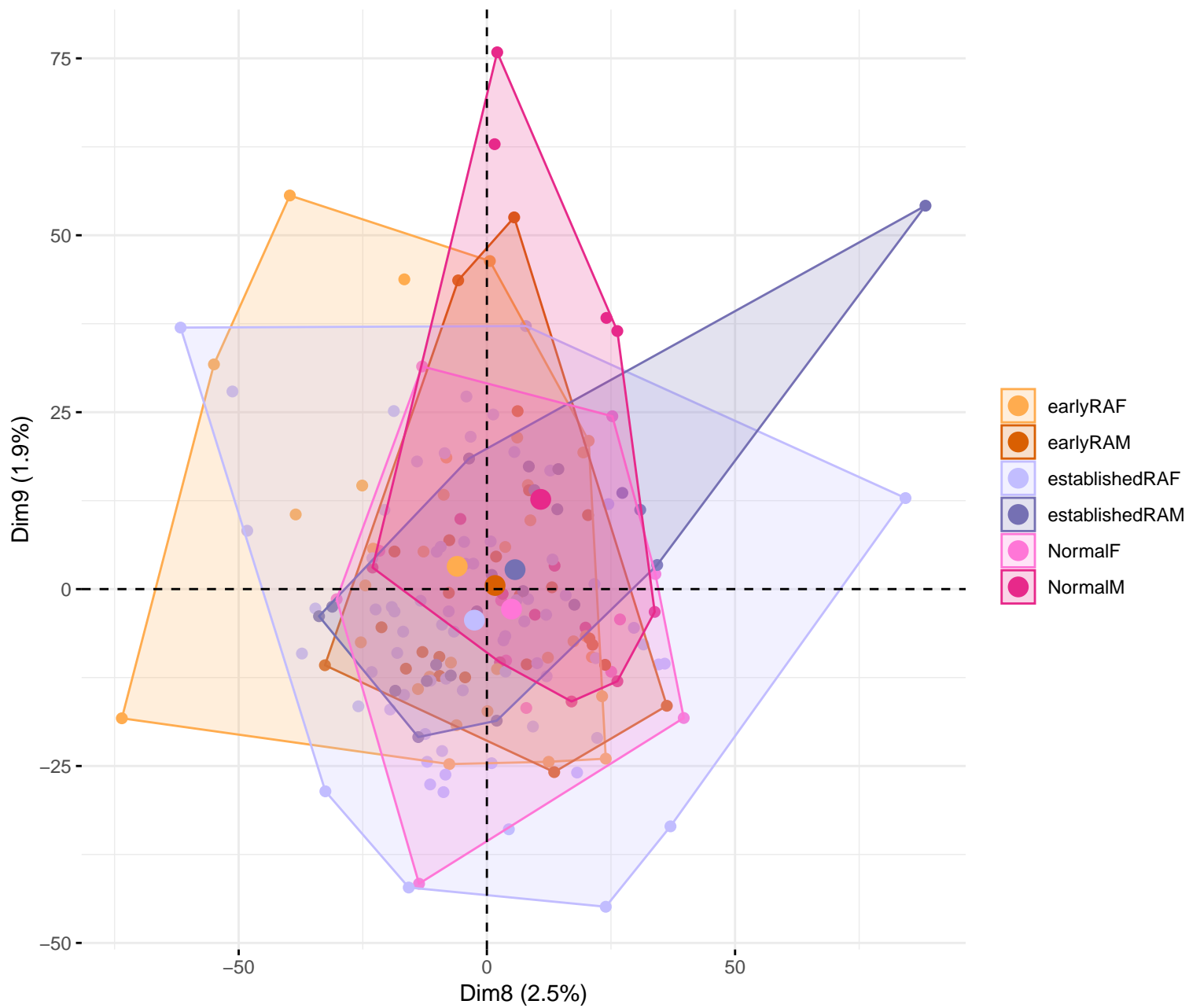

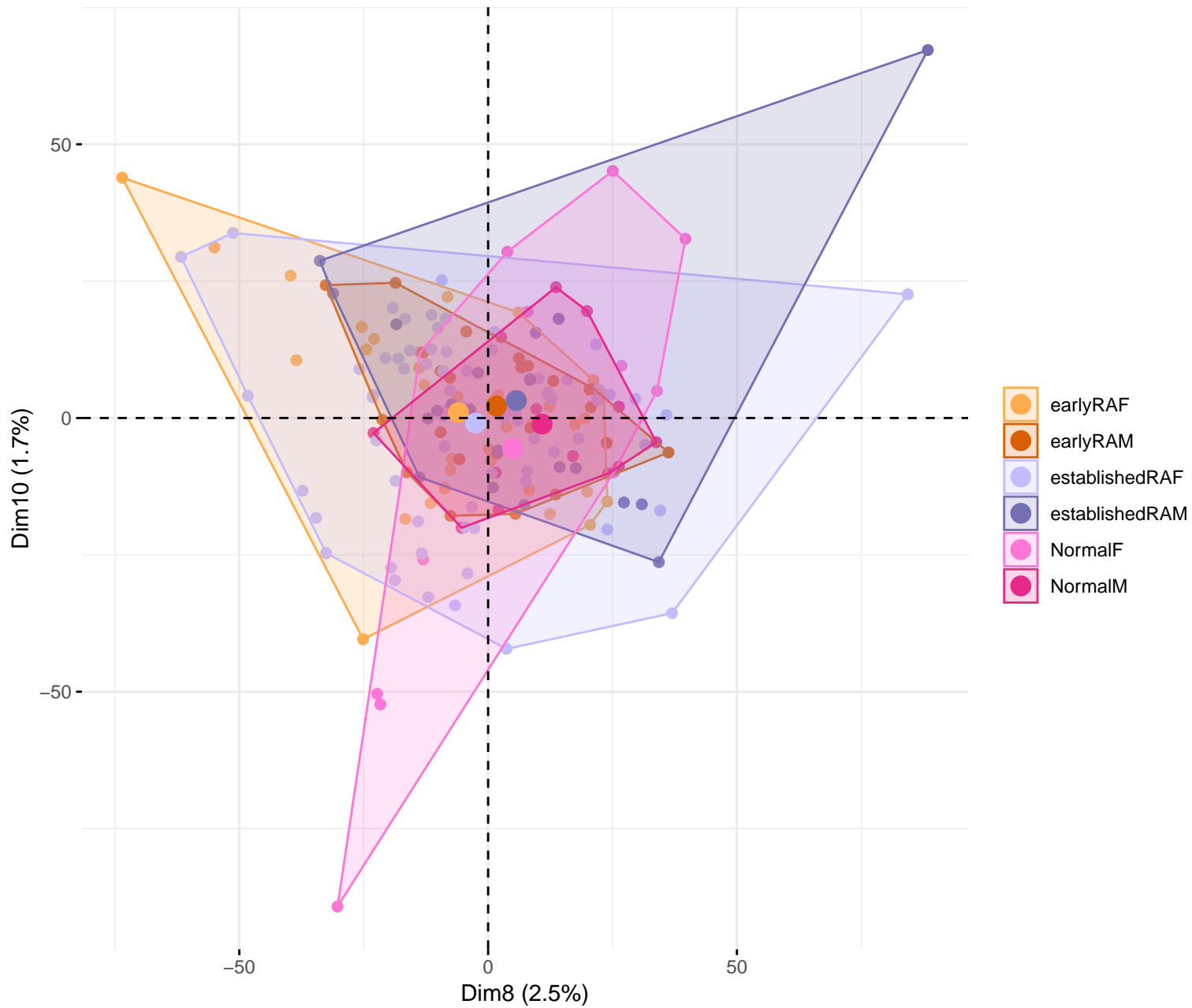

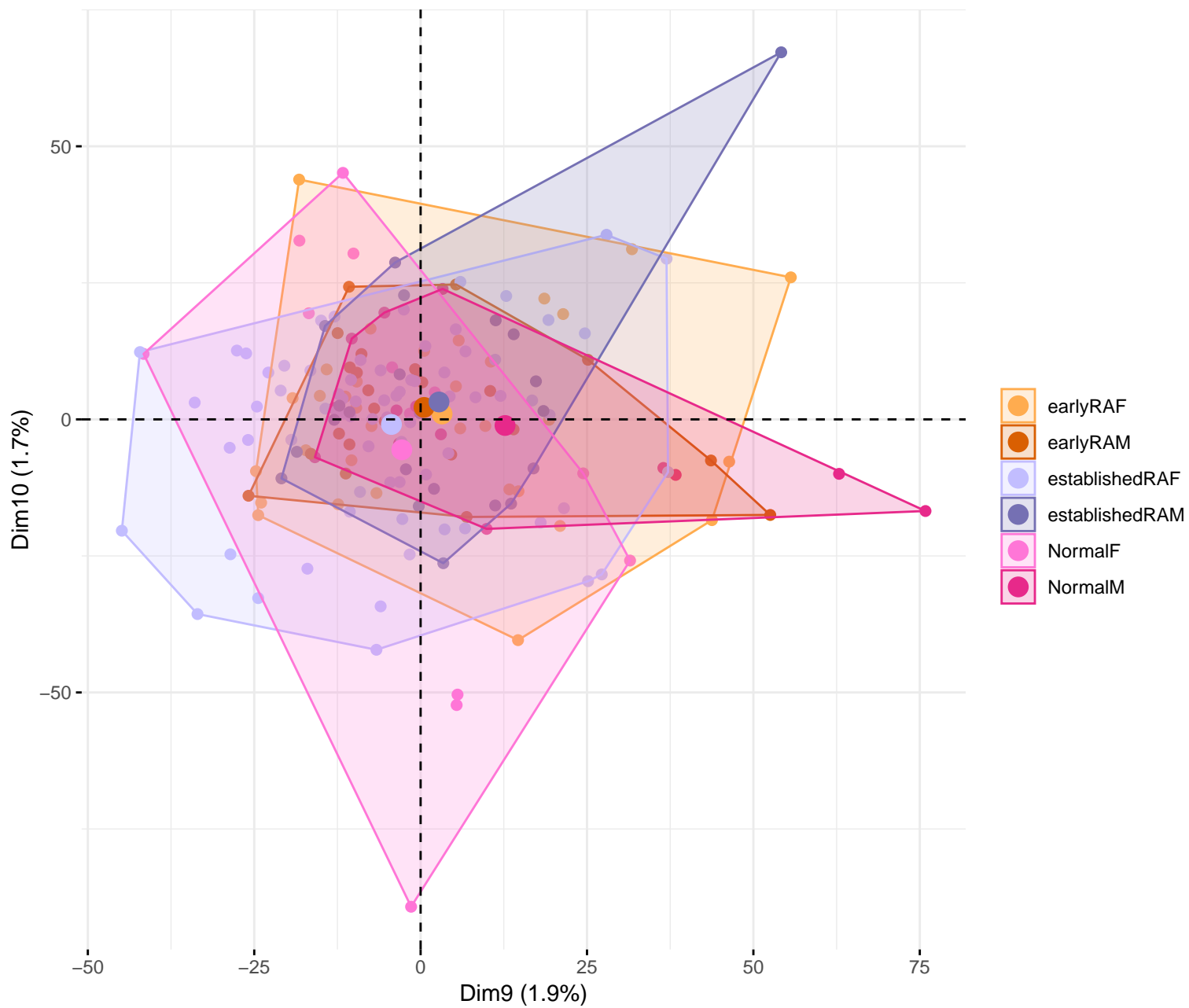

Supplement: S5 Fig — The areas are the convex hull of the condition. The largest point of one color depicts the centers of the hull. Only those conditions are shown where more than ten samples were available for male and female individuals. Number of samples: 33 earlyRAF, 24 earlyRAM, 73 establishedRAF, 22 establishedRAM, 13 NormalF, 14 NormalM. (PDF) [file pone.0219698.s005.pdf]

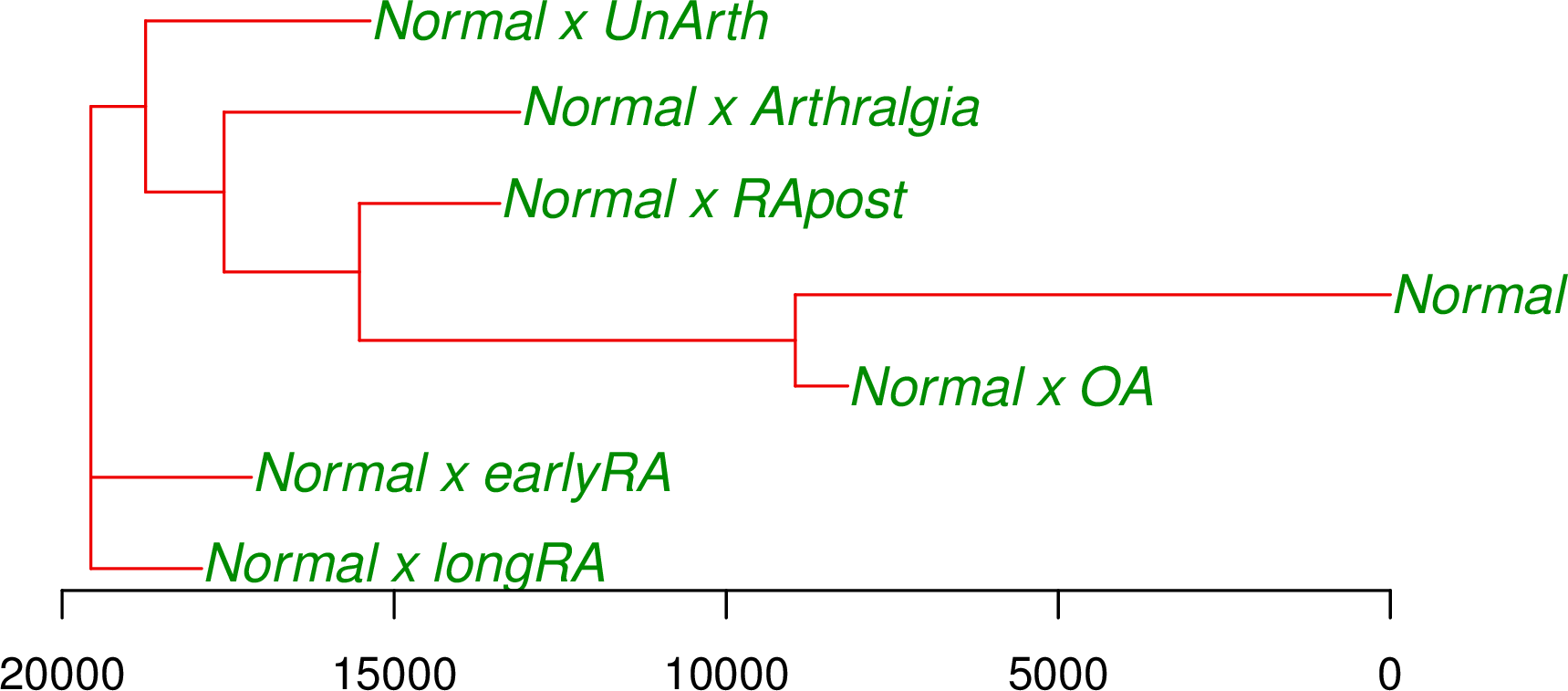

Supplement: S6 Fig — Origin for the fold-changes is normal/healthy (normal/healthy is the 0-vector). The x-axis is based on the Manhattan distance of significant fold-changes. The distance might be meaningless as an absolute value, but informative as relative distance. (TIF) [file pone.0219698.s006.tif]

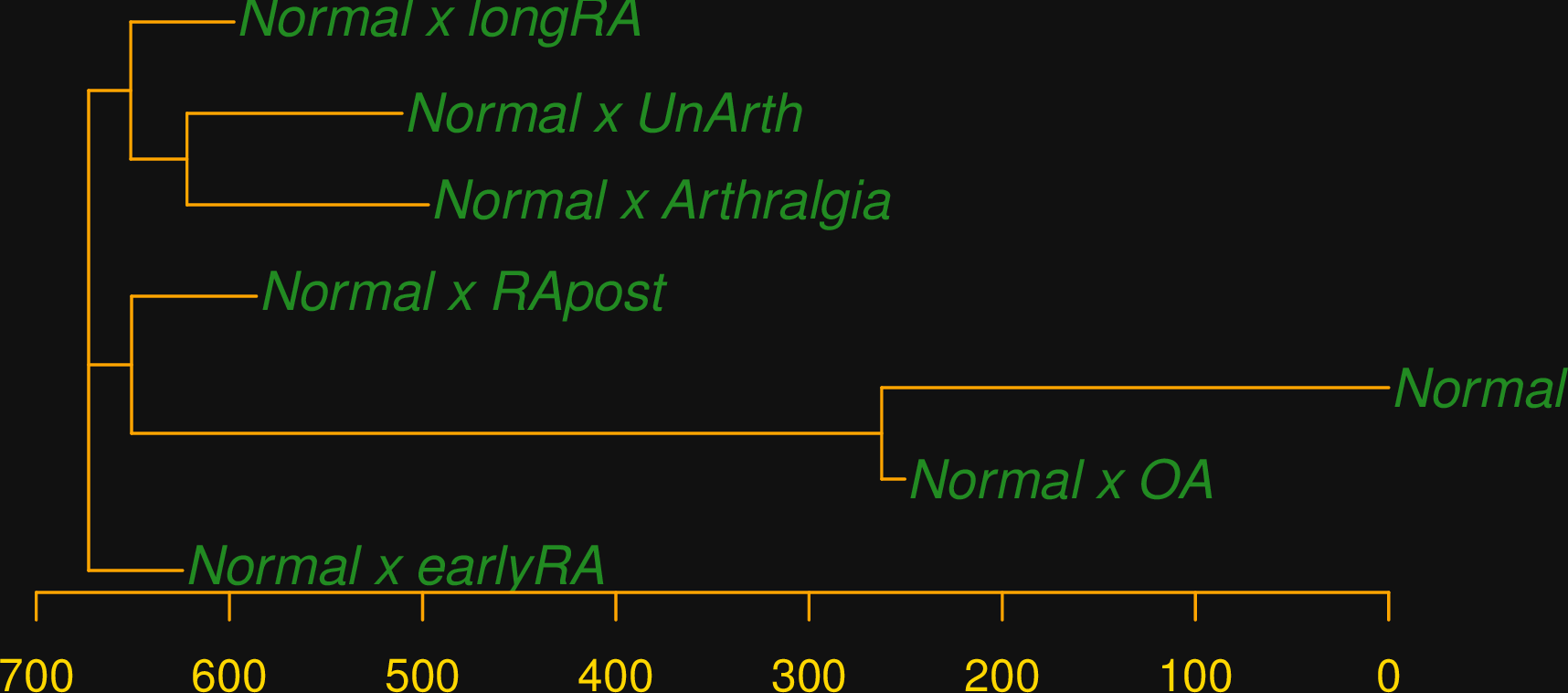

Supplement: S7 Fig — Origin for the fold-changes is normal/healthy (normal/healthy is the 0-vector). The x-axis is the distance. The distance might be meaningless as an absolute value, but informative as relative distance. (TIF) [file pone.0219698.s007.tif]

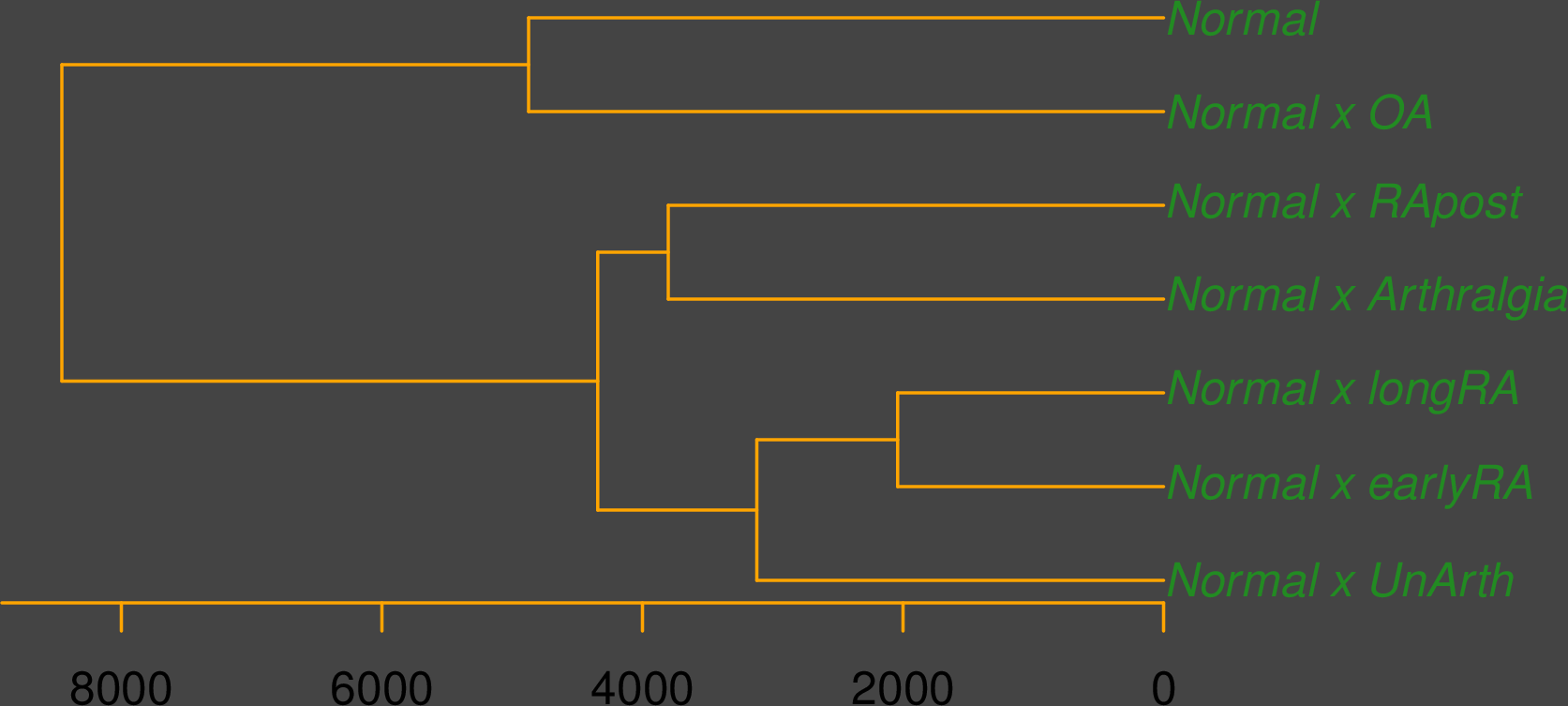

Supplement: S8 Fig — Origin for the fold-changes is normal/healthy (normal/healthy is the 0-vector). The x-axis is the distance. The distance might be meaningless as an absolute value, but informative as relative distance. (TIF) [file pone.0219698.s008.tif]

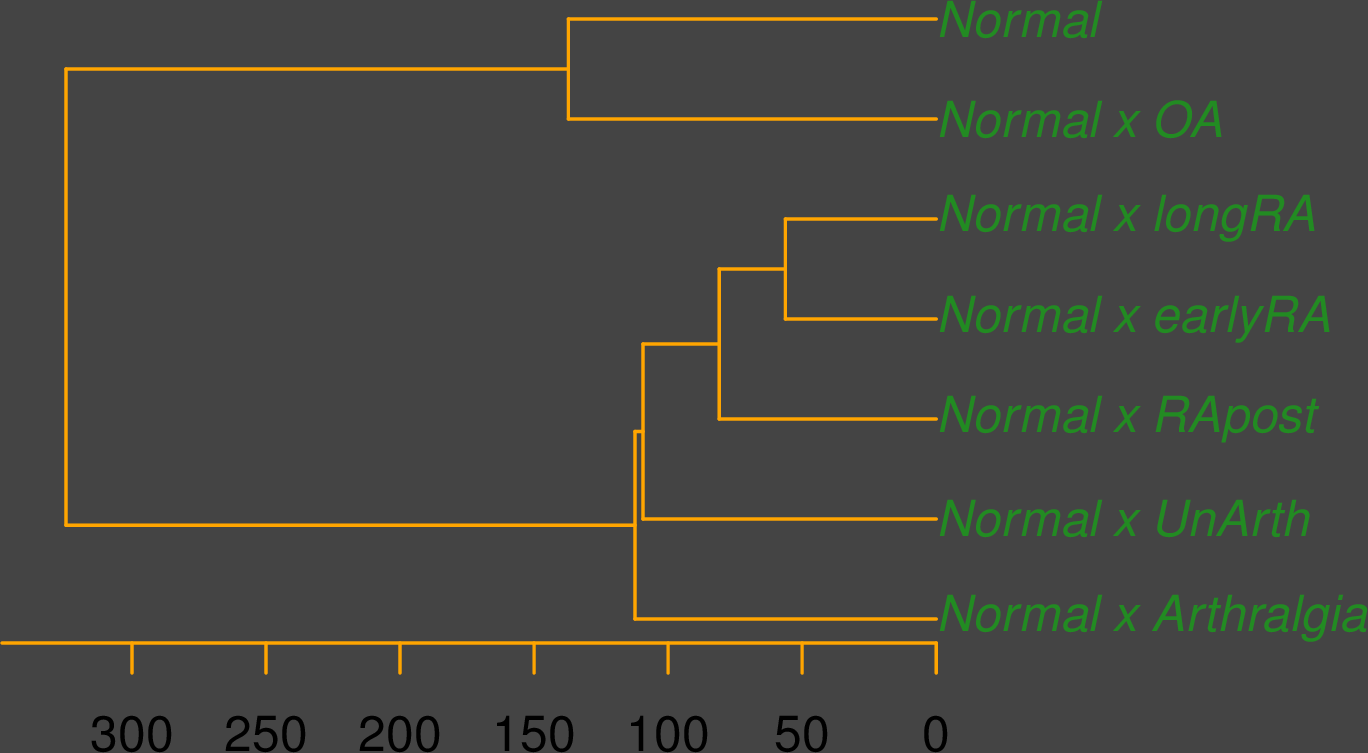

Supplement: S9 Fig — Origin for the fold-changes is normal/healthy (normal/healthy is the 0-vector). The x-axis is the distance. The distance might be meaningless as an absolute value, but informative as relative distance. (TIF) [file pone.0219698.s009.tif]

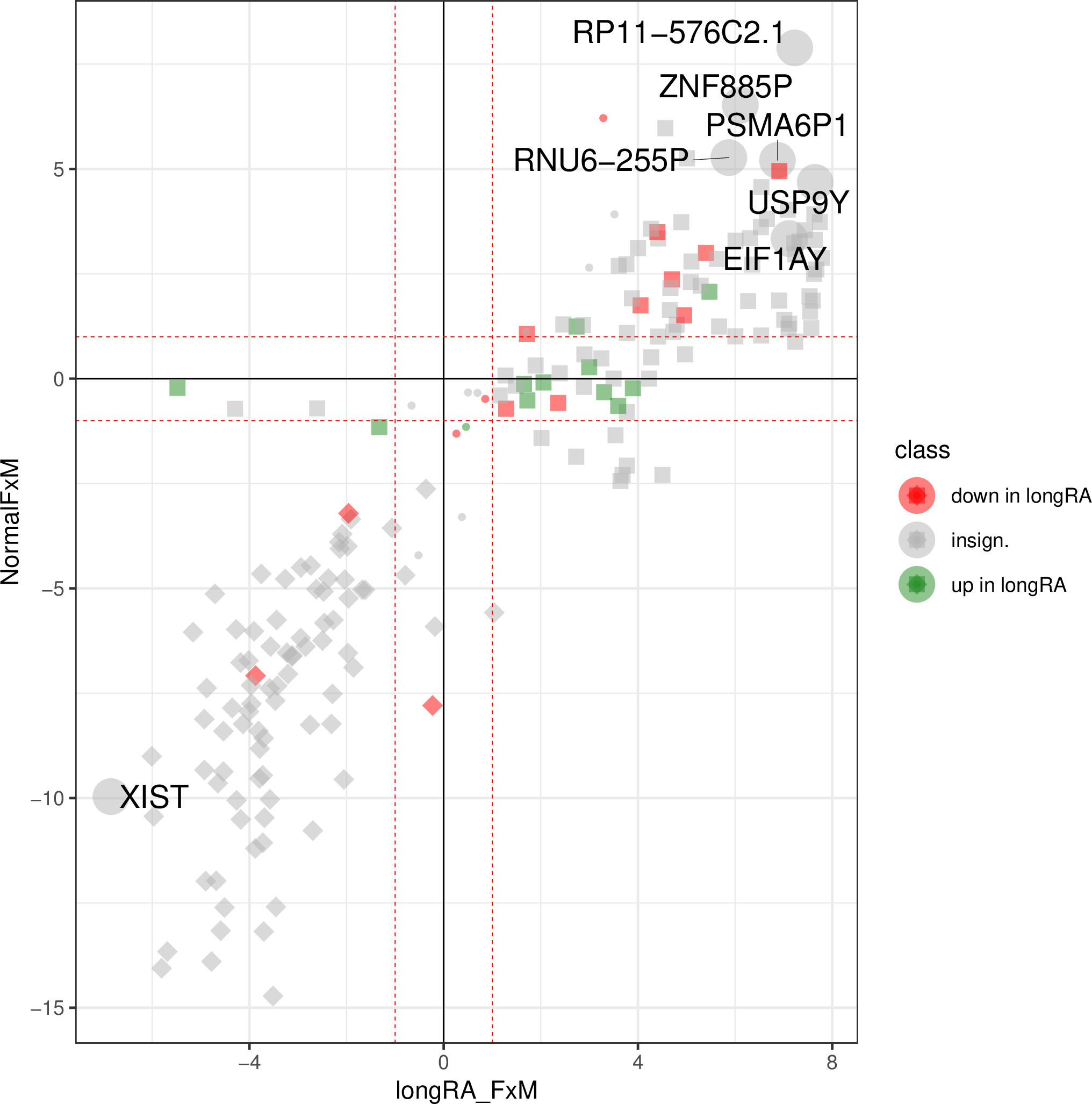

Supplement: S10 Fig — Only genes are shown which are significantly differentially expressed in men and women. The size and shape shows the significance in differences of men and women: the large circles are genes significantly differentially expressed between the sexes in established/long RA and normal condition, these genes are also labelled. Small squares mean a significant difference between men and woman only in established/long RA, small diamonds mean a significant difference only between healthy men and woman. The color represents the significance of the difference in expression between normal and established/long RA. (TIF) [file pone.0219698.s010.tif]

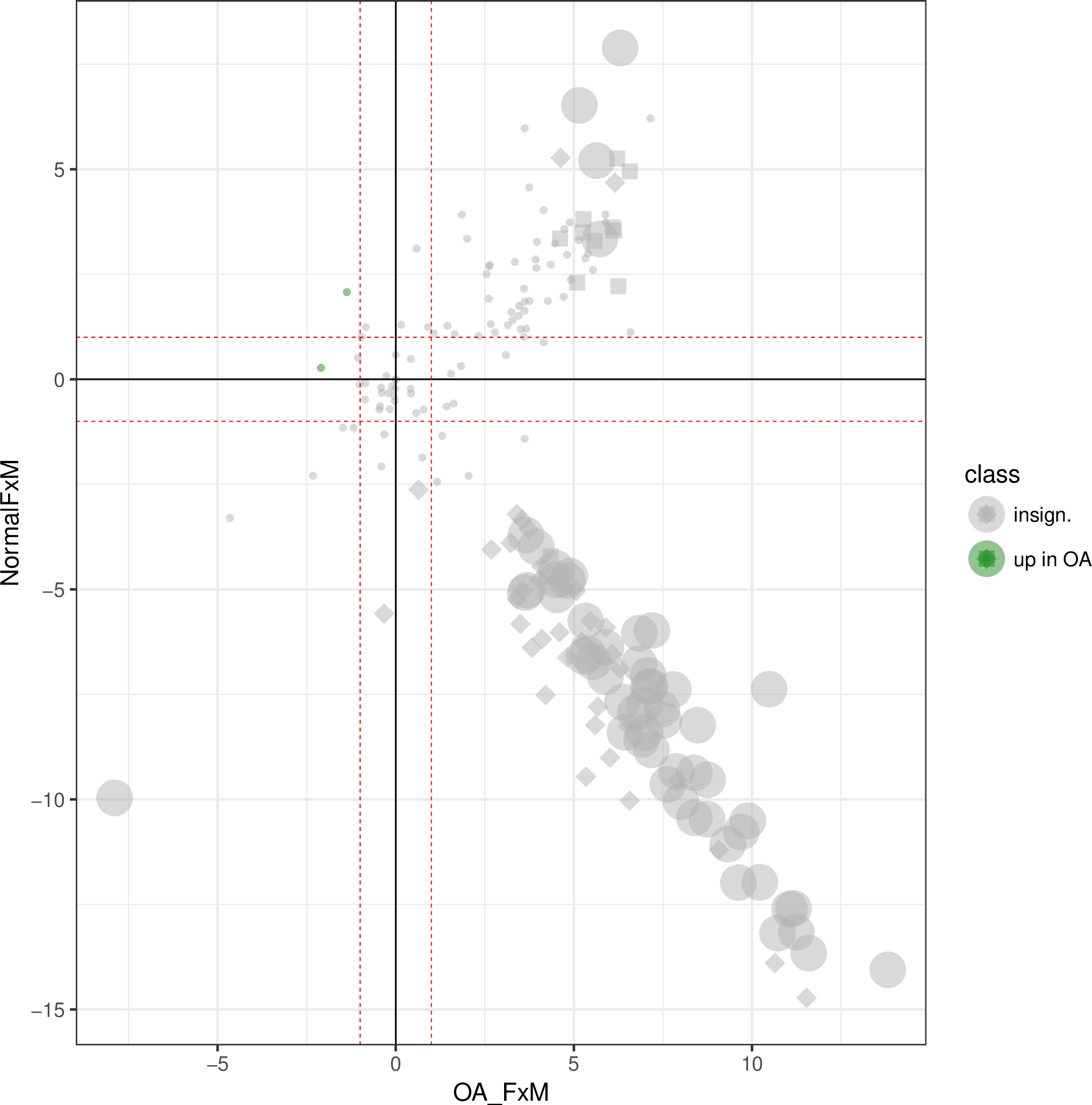

Supplement: S11 Fig — Only genes are shown which are significantly differentially expressed in men and women. The size and shape shows the significance in differences of men and women: the large circles are genes significantly differentially expressed between the sexes in OA and normal condition. Small squares mean a significant difference between men and woman only in OA, small diamonds mean a significant difference only between healthy men and woman. The color represents the significance of the difference in expression between normal and OA. (TIF) [file pone.0219698.s011.tif]

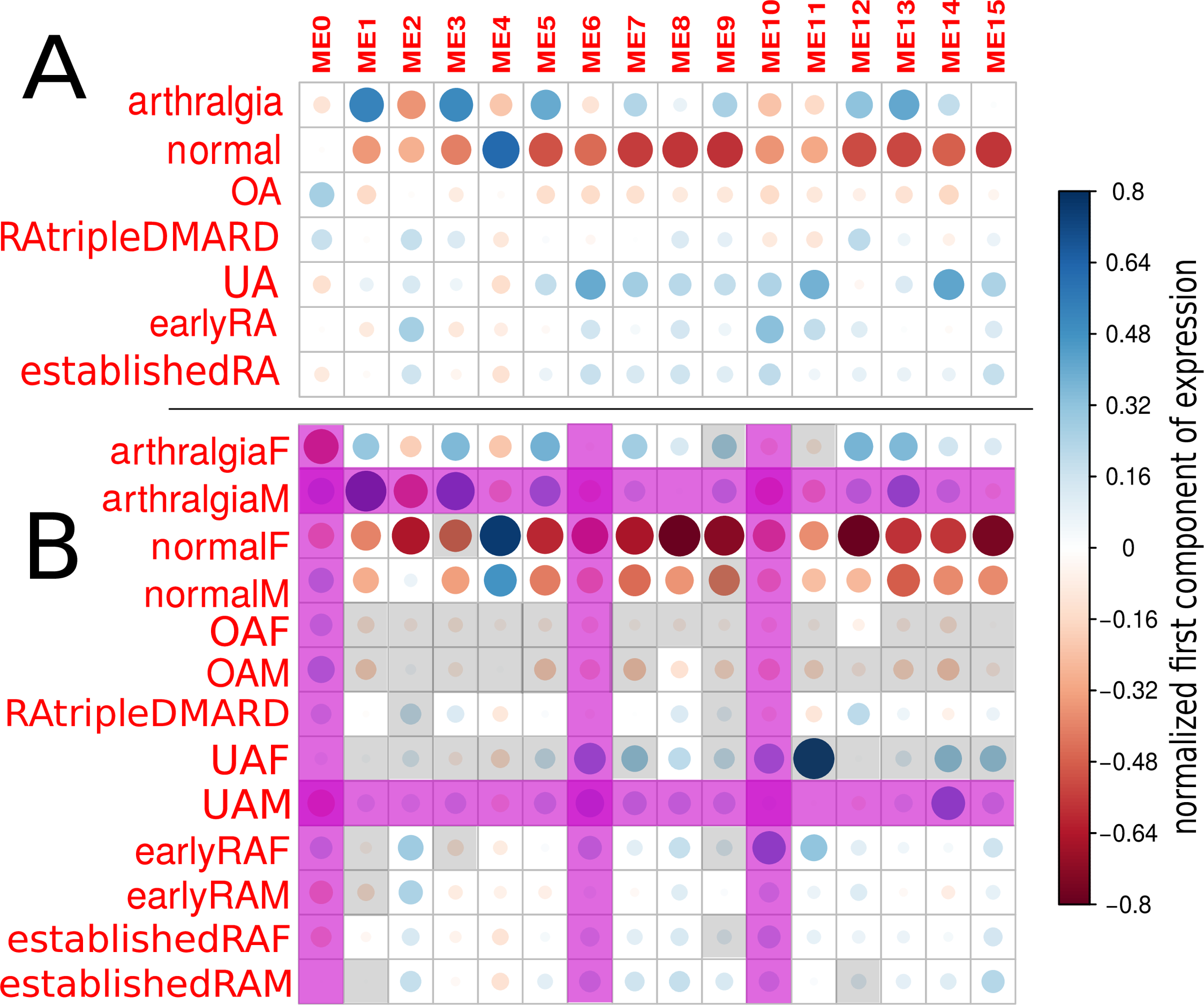

Supplement: S12 Fig — Genes were clustered into modules of co-expression and their module eigengenes (ME) normalized expressions are shown here for all conditions. In panel B the patients are split in male/female and diagnosis, in panel A the average value of male and female is shown. The color and the size of the dots depict the first principal component per module using the scaled expression of the respective genes of a module over all conditions. Parts of panel B should be seen with caution as some groups have a very small sample size (arthralgiaM and UAM). Pairwise significant differences of conditions within modules are in S5 Table. These are summarized in panel B as color-code: purple rows and columns are not significantly different to any condition in any module, gray cells are not significantly different to any condition within the particular module. Number of samples: 8 arthralgiaF, 2 arthralgiaM, 13 normalF, 14 normalM, 13 OAF, 9 OAM, 19 RAtripleDMARD, 5 UAF, 1 UAM, 33 earlyRAF, 24 earlyRAM, 73 establishedRAF and 22 establishedRAM. (TIF) [file pone.0219698.s012.tif]

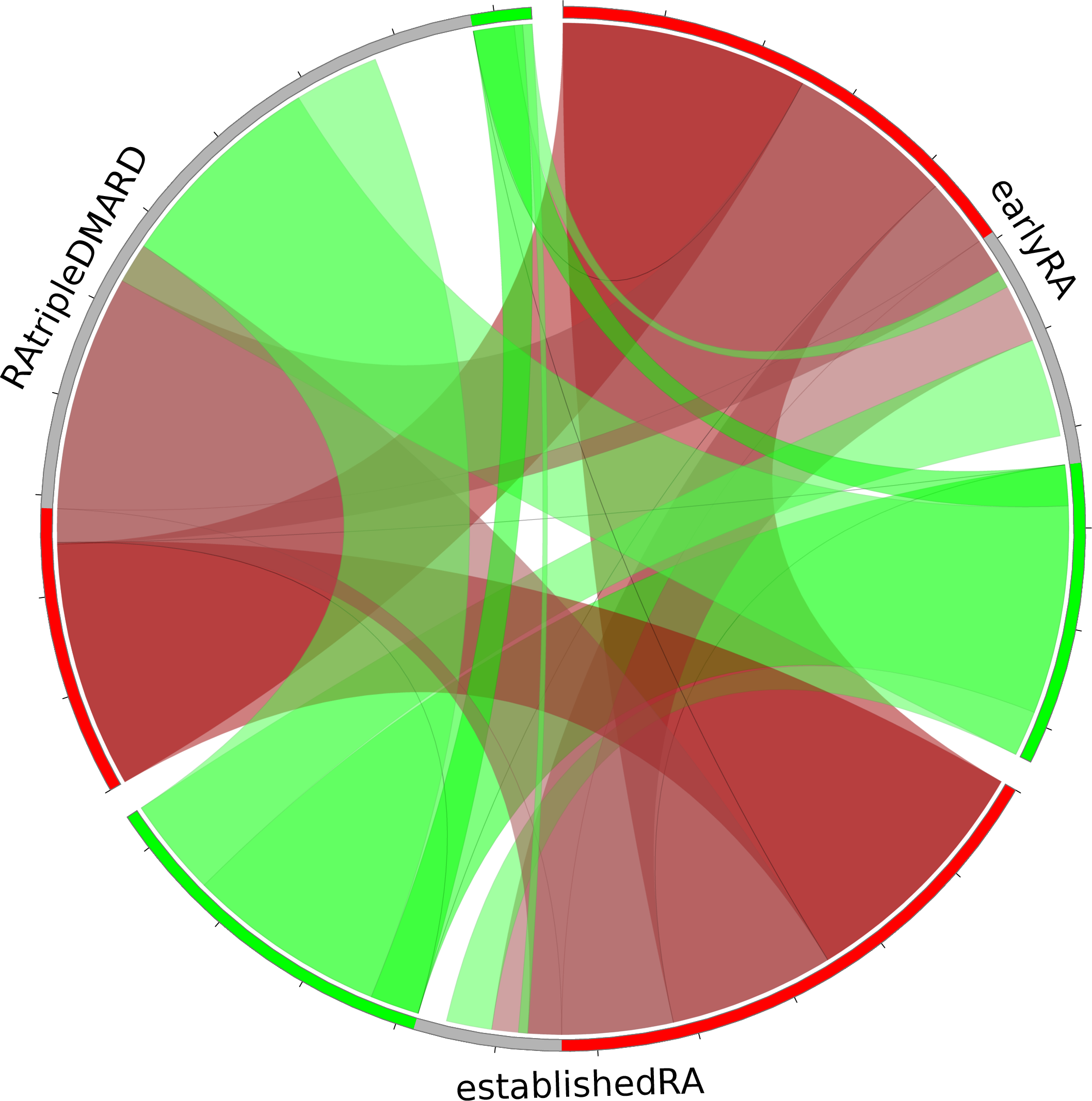

Supplement: S13 Fig — The base condition is normal, so the label ‘earlyRA’ means normal compared with early RA. Up-regulated fractions are shown in green, down-regulated fractions are shown in red; gray are fractions of genes which are not significantly differentially expressed. The full set is the union of significantly differentially expression genes in all comparisons. The colors of the arc connections are dependent on what they are connecting. Number of samples: 57 earlyRA, 95 establishedRA, 27 normal and 19 RAtripleDMARD. (TIF) [file pone.0219698.s013.tif]

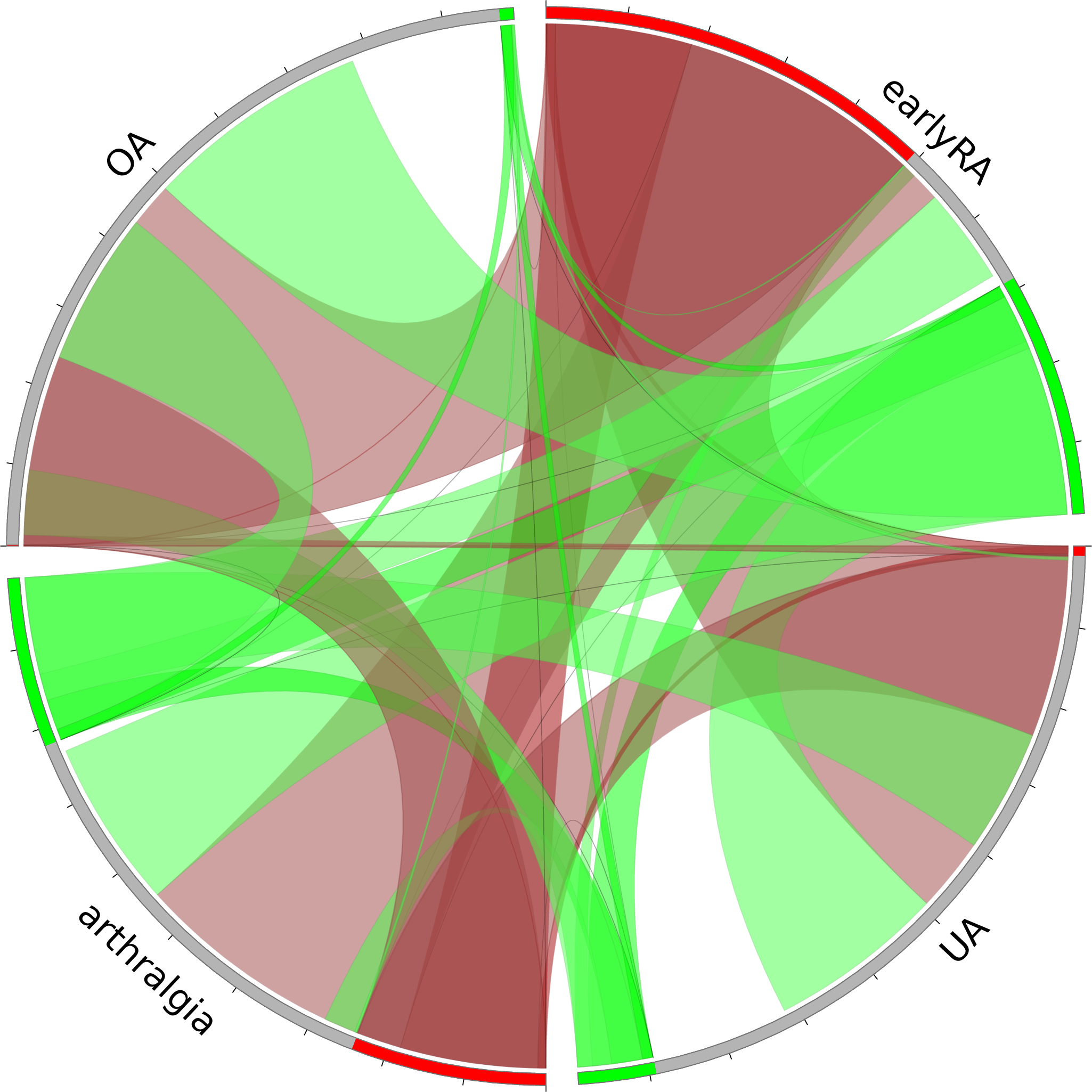

Supplement: S14 Fig — The base condition is normal, so the label ‘earlyRA’ means normal compared with early RA. Up-regulated fractions are shown in green, down-regulated fractions in red; in gray are fractions of genes which are not significantly differentially expressed. The full set is the union of significantly differentially expression genes in all comparisons in this Fig. The colors of the arc connections are dependent on what they are connecting. Number of samples: 10 arthralgia, 57 earlyRA, 27 normal, 22 OA and 6 UA. (TIF) [file pone.0219698.s014.tif]

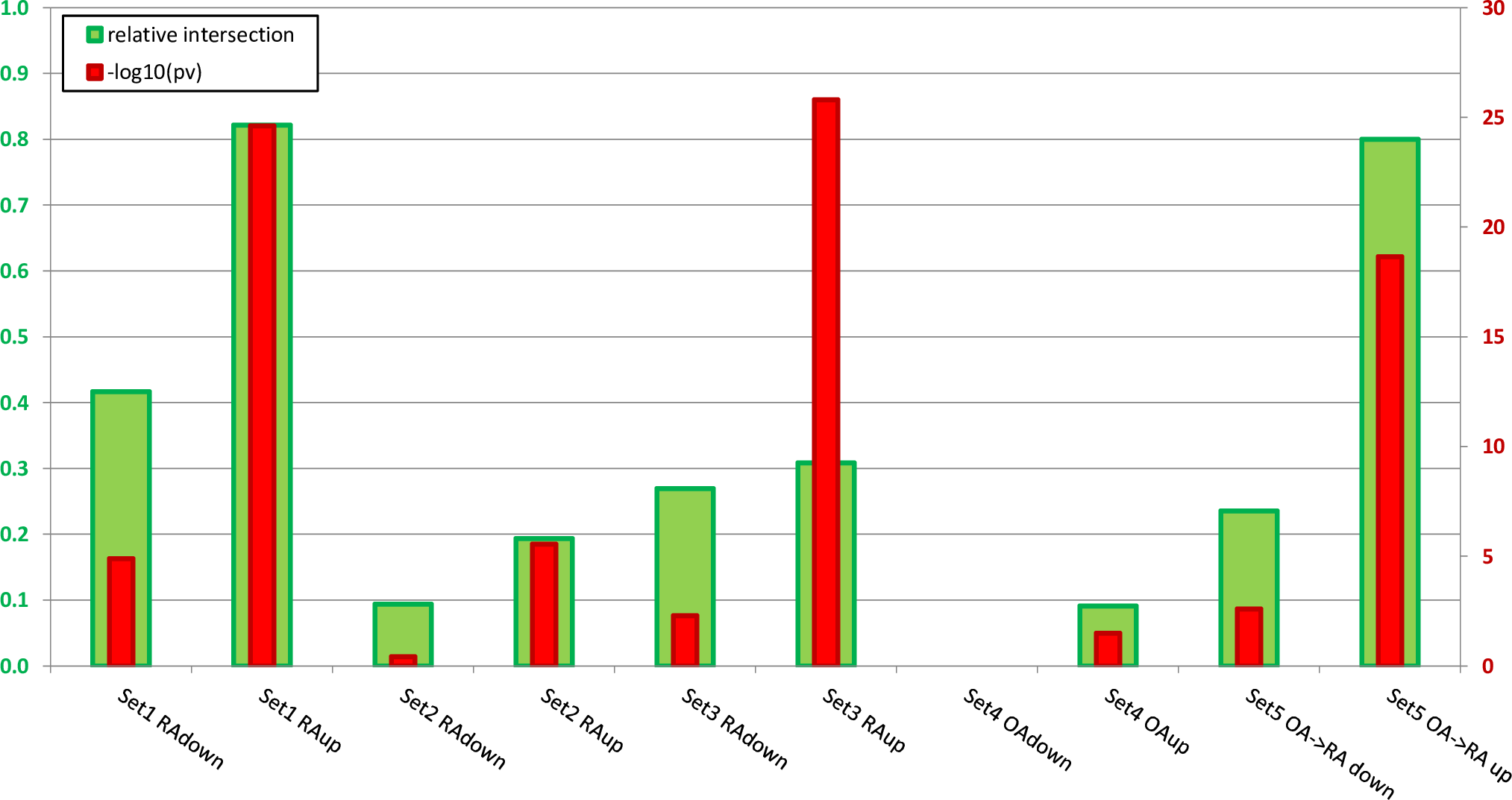

Supplement: S15 Fig — The base state of genes is the normal condition, except for ‘OA->RA’ where it is OA (base state is the first one in ‘condition compared to condition ‘). The origins of the different sources or sets (the 'Set1' to 'Set5') are listed in Table B in S1 Text. (TIF) [file pone.0219698.s015.tif]
