# Supplementary material for: Analysis of gene expression in rheumatoid arthritis and related conditions offers insights into sex-bias, gene biotypes and co-expression patterns
Source: PLoS One. 2019 Jul 25;14(7):e0219698. doi: 10.1371/journal.pone.0219698 (PMC6657850; doi:10.1371/journal.pone.0219698)
Supplement: S1 Text — Contains the sections 'Clustering of genes', 'Overlaps in differentially expressed genes between clinical conditions' and 'Comparisons with other studies'. (DOCX) [file pone.0219698.s021.docx]

supplemental text for

**Analysis of gene expression in rheumatoid arthritis and related conditions offers insights into sex-bias, gene biotypes and co-expression patterns**

Alexander Platzer^1*^, Thomas Nussbaumer^2,3^, Thomas Karonitsch^1^, Josef S. Smolen^1^, Daniel Aletaha^1^

^1^ Division of Rheumatology, Department of Medicine III, Medical University of Vienna, Vienna, Austria

^2^ Chair and Institute of Environmental Medicine, UNIKA-T, Technical University and Helmholtz Zentrum München, Augsburg, Germany.

^3^ Institute of Network Biology (INET), Helmholtz Center Munich, Ingolstädter Landstraße 1, 85764 Neuherberg, Germany

**Clustering of genes**

The Weighted gene correlation network analysis (WGCNA) was applied to the 236 RNA-seq synovial biopsy samples to assign genes into modules when they share a similar expression pattern along all conditions. From the total set of 56,292 genes, 11,692 genes remained after filtering (see Material and Methods), and were grouped into 16 modules (S12 Fig). Each module has an eigengene (so called module eigengene or ME), which is given by the first component of a PCA. Functional enrichments are in the Supplementary Archive tables/ , some remarkable functional enrichments are in Table A.

**S12 Fig**. **Gene clusters.** Genes were clustered into modules of co-expression and their module eigengenes (ME) normalized expressions are shown here for all conditions. In panel B the patients are split in male/female and diagnosis, in panel A the average value of male and female is shown. The color and the size of the dots depict the first principal component per module using the scaled expression of the respective genes of a module over all conditions. Parts of panel B should be seen with caution as some groups have a very small sample size (arthralgiaM and UAM). Pairwise significant differences of conditions within modules are in S5 Table. These are summarized in panel B as color-code: purple rows and columns are not significantly different to any condition in any module, gray cells are not significantly different to any condition within the particular module. Number of samples: 8 arthralgiaF, 2 arthralgiaM, 13 normalF, 14 normalM, 13 OAF, 9 OAM, 19 RAtripleDMARD, 5 UAF, 1 UAM, 33 earlyRAF, 24 earlyRAM, 73 establishedRAF and 22 establishedRAM.

**S5 Table. Pairwise significant differences of conditions within modules of the Weighted gene correlation network analysis (WGCNA).** The differences were tested with a Wilcoxon test comparing the eigengene values of the samples of two conditions. The 'significant?' column is on the Bonferroni-corrected p-value. Each combination is twice in the table (e.g. NormalF vs. earlyRAF and earlyRAF vs. NormalF are the same).

**Table A. Selected biological functions of modules.**

| **Module** | **Amount of genes** | **Functions**  **GO, KEGG, etc.** |
| --- | --- | --- |
| 2 | 1367 | cell motility; cell migration; cytoskeleton changes |
| 3 | 1247 | cell adhesion, aggregation |
| 5 | 1151 | mitochondrial changes |
| 7+8 | 637 | neutrophil and myeloid cell activation |
| 11 | 382 | activation of the complement system; FCGR activation;  FCERI -> MAPK and NF-kB activation |
| 13 | 277 | neutrophil aggregation |
| 15 | 158 | blood coagulation |

The genes of the modules were used as input for gene enrichment analysis.

**Overlaps in differentially expressed genes between clinical conditions**

A viewpoint on how much the different conditions are overlapping in the 236 RNA-seq synovial biopsy samples in terms of gene expression is given by the overlap of significantly differentially expressed genes. Up- and down-regulated genes were compared between normal to RA conditions along with their overlaps in S13 Fig. The total set of genes therein is the same for each node (this is e.g. the part of the circle labelled ‘earlyRA’) and is the union of all significantly differentially expressed genes in any comparison shown. In total, these are 10,167 genes for RA conditions (early RA, established RA and RAtripleDMARD; differentially expressed compared to normal condition as base state) and 9,878 genes for the combination early RA, arthralgia, OA and undifferentiated arthritis (differentially expressed compared to normal condition as base state). S13 Fig depicts that RAtripleDMARD has the fewest up-regulated genes, which agrees with Fig 1 where RAtripleDMARD is closer to the normal condition than early and late RA stage, and only few genes are both up- and down-regulated within different RA conditions when compared to normal. The same analysis is shown for other conditions than classified RA in S14 Fig. Besides the amount of all up- and down-regulated genes at one glance, arthralgia shares most of the down-regulated genes with early RA, but only few of the up-regulated genes. The gene sets of UA and arthralgia are less solid than the others as their sample sizes are lower (6 and 10 samples). All fold-change tables and gene sets are available in the Supplement.

In addition, the number of genes shared in the three non-RA conditions (UA, OA, arthralgia) is smaller than the number of genes shared among the RA-conditions. While among the RA conditions 69-84% of upregulated and 85-88% of downregulated genes are shared, the fractions are 41-85% upregulated and 6-98% downregulated genes when normal is compared to UA, OA and arthralgia (pairwise overlaps where the base for 100% is the smaller set; the extreme ratios are because of the small absolute values).

**S13 Fig. The amount and the overlaps of up- and down-regulated genes for different RA conditions.** The base condition is normal, so the label ‘earlyRA’ means normal compared with early RA. Up-regulated fractions are shown in green, down-regulated fractions are shown in red; gray are fractions of genes which are not significantly differentially expressed. The full set is the union of significantly differentially expression genes in all comparisons. The colors of the arc connections are dependent on what they are connecting. Number of samples: 57 earlyRA, 95 establishedRA, 27 normal and 19 RAtripleDMARD.

**S14 Fig. The amount and the overlaps of up- and down-regulated genes for early RA, OA, arthralgia and undifferentiated arthritis.** The base condition is normal, so the label ‘earlyRA’ means normal compared with early RA. Up-regulated fractions are shown in green, down-regulated fractions in red; in gray are fractions of genes which are not significantly differentially expressed. The full set is the union of significantly differentially expression genes in all comparisons in this Fig. The colors of the arc connections are dependent on what they are connecting. Number of samples: 10 arthralgia, 57 earlyRA, 27 normal, 22 OA and 6 UA.

**Comparisons with other studies**

For checking consistency with previously published studies, we compared our derived lists of significantly differentially expressed genes in the RNA-seq samples with the microarray studies of Liu et al., Niu et al., Teixeira et al. and Yoshida et at. [1-4]. Liu et al. and Yoshida et at. extracted the samples from synovial tissue, whereas Niu et al. and Teixeira et al. extracted their samples from peripheral blood (Peripheral Blood Mononuclear Cells - PBMCs).

As expected from using the more sensitive RNA-seq data, our lists of significantly regulated genes are larger than the lists of the previous microarray studies. An overview is shown in S15 Fig. Genes from previous studies were only taken into account if they are contained in the annotation used in this article. The relative intersection is defined as size of overlap divided by the smaller of the two sets. Significance is calculated with the hypergeometric test to determine whether overlaps are significantly more than expected. The relative overlap as well as its significance is always higher for the up-regulated genes than for the down-regulated genes. As expected, the overlap is higher in the same tissue (RA synovium in Set1 and Set5 in S15 Fig) than in distinct tissues (PBMCs in Set2 and Set3). The weak overlap in OA is likely due to the small amount of DEGs (188 in the RNA-seq data and 21 in the microarray data) and the highly varying gene expression, which is also visible at a high level view (see Fig 1).

**S15 Fig. Comparison of significantly differentially expressed genes.** The base state of genes is the normal condition, except for ‘OA->RA’ where it is OA (base state is the first one in ‘condition <one> compared to condition <two>’). The origins of the different sources or sets (the 'Set1' to 'Set5') are listed in Table B.

**Table B. Sources of the microarray studies compared with our analysis.**

| **Label** | **Publication title** | **Reference** |
| --- | --- | --- |
| Set1 | Identifying genes related with rheumatoid arthritis via system biology analysis | [1] |
| Set2 | Transcriptome analysis describing new immunity and defense genes in peripheral blood mononuclear cells of rheumatoid arthritis patients. | [3] |
| Set3 | The shared crosstalk of multiple pathways involved in the inflammation between rheumatoid arthritis and coronary artery disease based on a digital gene expression profile | [2] |
| Set4 | Identifying genes related with rheumatoid arthritis via system biology analysis | [1] |
| Set5 | Gene expression analysis of rheumatoid arthritis synovial lining regions by cDNA microarray combined with laser microdissection: up- regulation of inflammation-associated STAT1, IRF1, CXCL9, CXCL10, and CCL5 | [4] |

Altogether, the overlaps with previous studies are as large as expected, where the recent data sources pointing on many more genes, because of general improvements of the data and the genome annotation.

# References

1. Liu T, Lin X, Yu H. Identifying genes related with rheumatoid arthritis via system biology analysis. Gene. 2015;571(1):97-106. Epub 2015/06/29. doi: 10.1016/j.gene.2015.06.058. PubMed PMID: 26117171.

2. Niu X, Lu C, Xiao C, Zhang Z, Jiang M, He D, et al. The shared crosstalk of multiple pathways involved in the inflammation between rheumatoid arthritis and coronary artery disease based on a digital gene expression profile. PLoS One. 2014;9(12):e113659. Epub 2014/12/17. doi: 10.1371/journal.pone.0113659. PubMed PMID: 25514790; PubMed Central PMCID: PMCPMC4267808.

3. Teixeira VH, Olaso R, Martin-Magniette ML, Lasbleiz S, Jacq L, Oliveira CR, et al. Transcriptome analysis describing new immunity and defense genes in peripheral blood mononuclear cells of rheumatoid arthritis patients. PLoS One. 2009;4(8):e6803. Epub 2009/08/28. doi: 10.1371/journal.pone.0006803. PubMed PMID: 19710928; PubMed Central PMCID: PMCPMC2729373.

4. Yoshida S, Arakawa F, Higuchi F, Ishibashi Y, Goto M, Sugita Y, et al. Gene expression analysis of rheumatoid arthritis synovial lining regions by cDNA microarray combined with laser microdissection: up-regulation of inflammation-associated STAT1, IRF1, CXCL9, CXCL10, and CCL5. Scand J Rheumatol. 2012;41(3):170-9. Epub 2012/03/10. doi: 10.3109/03009742.2011.623137. PubMed PMID: 22401175; PubMed Central PMCID: PMCPMC3400100.
